# Tumor-Preferential Induction of Immune Responses and Epidermal Cell Death in Actinic Keratoses by Ingenol Mebutate

**DOI:** 10.1371/journal.pone.0160096

**Published:** 2016-09-09

**Authors:** Steffen Emmert, Holger A. Haenssle, John R. Zibert, Margarete Schön, Andreas Hald, Maria H. Hansen, Thomas Litman, Michael P. Schön

**Affiliations:** 1 Department of Dermatology, Venereology and Allergology, University Medical Center Göttingen, Göttingen, Germany; 2 LEO Pharma A/S, Ballerup, Denmark; Kinki Daigaku, JAPAN

## Abstract

The rapid and strong clinical efficacy of the first-in-class, ingenol mebutate, against actinic keratosis (AK) has resulted in its recent approval. We conducted the first comprehensive analysis of the cellular and molecular mode of action of topical ingenol mebutate 0.05% gel in both AK and uninvolved skin of 26 patients in a phase I, single-center, open-label, within-patient comparison. As early as 1 day after application, ingenol mebutate induced profound epidermal cell death, along with a strong infiltrate of CD4^+^ and CD8^+^ T-cells, neutrophils, and macrophages. Endothelial ICAM-1 activation became evident after 2 days. The reaction pattern was significantly more pronounced in AK compared with uninvolved skin, suggesting a tumor-preferential mode of action. Extensive molecular analyses and transcriptomic profiling of mRNAs and microRNAs demonstrated alterations in gene clusters functionally associated with epidermal development, inflammation, innate immunity, and response to wounding. Ingenol mebutate reveals a unique mode of action linking directly to anti-tumoral effects.

***Trial Registration*:** ClinicalTrials.gov NCT01387711

## Introduction

Actinic keratosis (AK) is common in fair-skinned people. In Europe, prevalence ranges from 11% to 25% in people aged ≥40 years [1–4]. AK lesions are early pre-malignant epithelial skin tumors induced by chronic exposure to UV irradiation and are characterized histologically by dysregulated keratinocyte proliferation and abnormal epithelial architecture [5, 6]. Substantial evidence suggests the presence of non-clinically visible AK adjacent to clinical lesions [7]. Based on histomorphological criteria, activation of oncogenic signaling pathways, and gene expression profiling, AK lesions represent *in situ* squamous-cell carcinomas (SCC) [5, 6, 8–12]. Indeed, contiguous AK is present in 27–97% of SCC lesions on sun-damaged skin [10, 13–16].

Ingenol mebutate, a macrocyclic diterpene ester, occurs naturally in the sap of *Euphorbia peplus* and has only recently been fully synthesized [17]. It has been successfully evaluated in clinical trials as topical field therapy against AK lesions on the face or scalp and the trunk or extremities [18–21]. The understanding of its mode-of-action is based solely on *in vitro*-studies and *in vivo*-studies in mice [22, 23]. Ingenol mebutate penetrates the skin with high concentrations in the epidermis and lower in the dermis. At high concentration (>100 μM), ingenol mebutate induces rapid and direct epithelia-restricted cell death [24–26], whereas at low concentrations (10–1,000 nM), it is a specific activator of protein kinase C (PKC) leading to cytokine and chemokine release and vascular endothelial activation as well as neutrophil-mediated oxidative burst [27–31]. One study suggested that ingenol mebutate acts through P-gp—mediated absorptive transport leading to dermal penetration and vascular damage which contributed to an anticancer activity of Ing3A *in vivo* [32]

In order to comprehensively elucidate the pharmacodynamics of ingenol mebutate in a clinical setting, we present the first cellular and molecular data in humans. Our extensive study comprises clinical, histological, and immunohistochemical as well as transcriptomic, mRNA, and microRNA (miRNA) profiling data of AK lesions. In addition, this is the first comparison of untreated and ingenol mebutate-treated AK lesions with non-involved, aged, but non-sun-damaged skin within the same patients.

## Results

**Fig 1:**
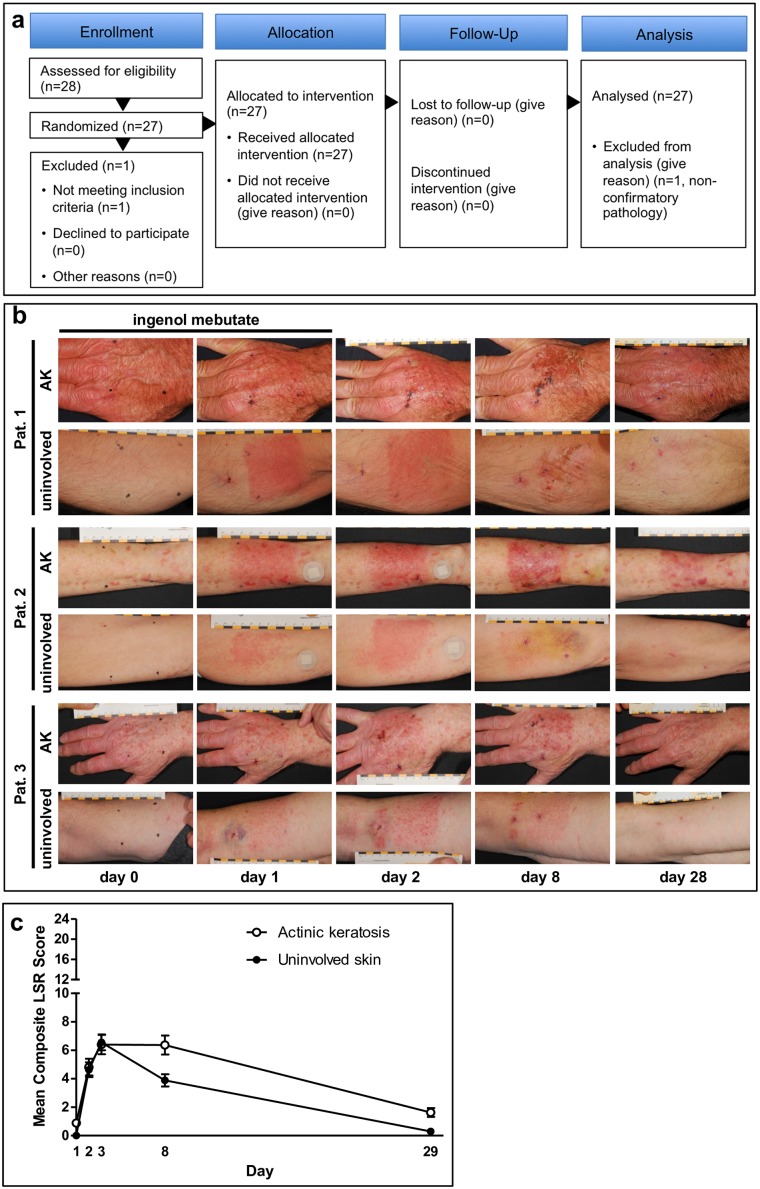
Inflammatory skin reactions induced by ingenol mebutate 0.05% gel are more pronounced in actinic keratosis (AK)-lesioned areas compared with uninvolved skin. **(A)** CONSORT study diagram. This was a phase I, single-center, open-label, within-patient comparison trial to explore the biological effects of ingenol mebutate gel applied once daily for 2 consecutive days in patients with actinic keratosis (AK) in a 25-cm^2^ area on the extremities and a 25-cm^2^ area of uninvolved-skin (US) on the inner upper arm. **(B)** Typical skin reactions during the course of the trial in AK treatment areas of three representative patients at the dorsum of the hand (upper panel) (*n* = 26) and uninvolved skin of the inner upper arm (lower panel) (*n* = 26) at day 0 (baseline), day 1 (after one treatment application), and day 2 (after two treatment applications) as well as during follow-up at days 8 and 29. **(B)** The composite local skin response (LSR) score is the sum of six individual LSR scores including erythema, flaking/scaling, crusting, swelling, vesiculation/pustulation, and erosion/ulceration, which range from 0 to 4, with higher numbers indicating more severe reactions. This was calculated at each study visit for each patient, with a theoretical maximum composite score of 24. Patients were assessed on days 0, 1, 2, 8, and 29. No unexpected signs of local or systemic toxicity were noted. Illustrated are averages of the LSR; error bars indicate standard error of the mean.

In total, 28 patients were enrolled and 27 were treated with ingenol mebutate 0.05% gel within a 25-cm^2^ field containing AK lesions on the dorsum of their hands or forearms, and another 25-cm^2^ area of uninvolved, normal-appearing, aged but non-sun-exposed skin on the inner aspect of the upper arm once daily on 2 consecutive days (Fig 1a; S1 Fig). The number of patients enrolled (*n* = 26 for each evaluated condition) ensured adequate power (0.8) to detect a significant effect size. Data were found to follow a normal distribution.

### Local skin responses and AK lesion elimination

In all patients, in both the AK and uninvolved skin, ingenol mebutate treatment induced predictable onset, peak, and resolution of local skin responses (LSR; Fig 1b and 1c) and was well tolerated, as previously reported in AK-fields [33], with a prolonged duration of the LSR intensity in AK treatment areas (day 8, *P* = 0.01; day 28, *P* = 0.001; Fig 1c), generally completely resolved 4 weeks after treatment initiation (Fig 1b). LSRs were dominated by erythema and, to a lesser extent, flaking/scaling, swelling, and vesiculation/pustulation.

### Treated versus uninvolved skin

**Fig 2:**
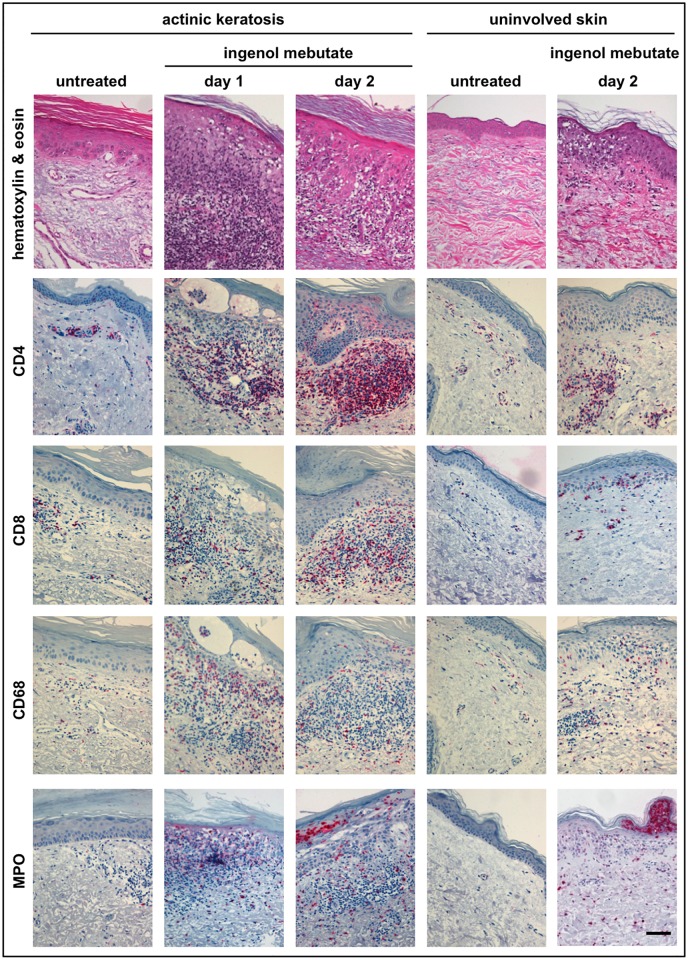
Ingenol mebutate 0.05% gel treatment causes rapid infiltration of T-cells, macrophages, and neutrophilic granulocytes. Biopsy specimens from all patients (*n* = 26) were subjected to histopathological evaluation. Skin tissues from the two treatment areas and all time points were assessed, and representative images are depicted. The panels depict hematoxylin & eosin staining as well as immunohistochemical staining for CD4^+^ T-lymphocytes, CD8^+^ T-lymphocytes, CD68^+^ macrophages/histiocytes, and myeloperoxidase (MPO^+^) neutrophils as indicated. The scale bar represents 100 μm.

**Fig 3:**
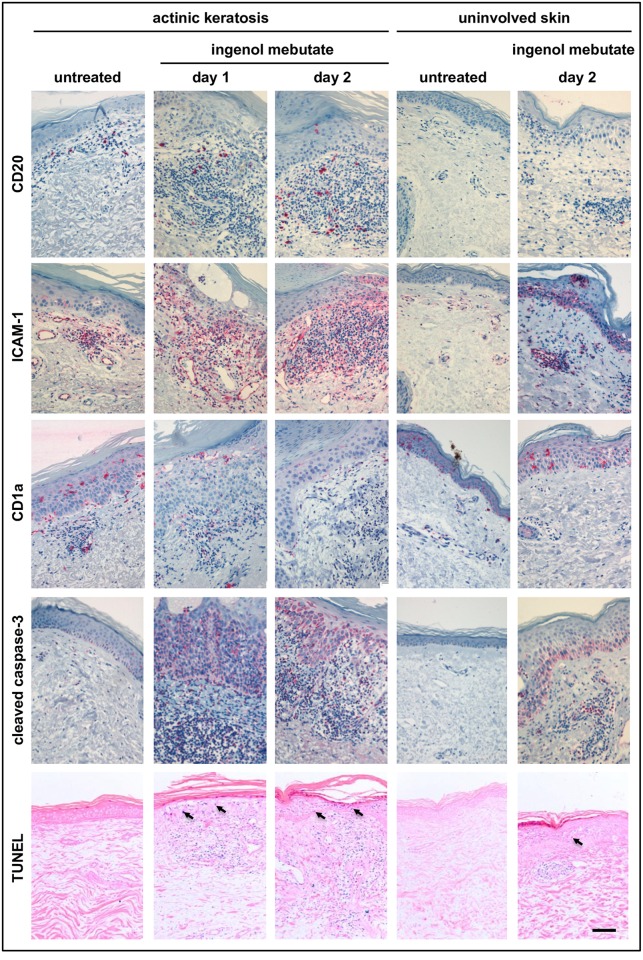
Ingenol mebutate 0.05% gel activates cutaneous blood vessels and induces epidermal cell death. Biopsy specimens from both treatment areas of all patients (*n* = 26) and all time points were assessed immunohistochemically for expression of CD20^+^ B-lymphocytes, ICAM-1 (CD54), and CD1a^+^ cells. Cleaved caspase 3 and TdT-mediated dUTP-biotin nick end labeling (TUNEL) reactivity indicating apoptotic responses were detected in five of these patients (arrows indicate examples of TUNEL positive epidermal cells). The figure depicts representative slides from all stainings as indicated. The scale bar represents 100 μm.

**Fig 4:**
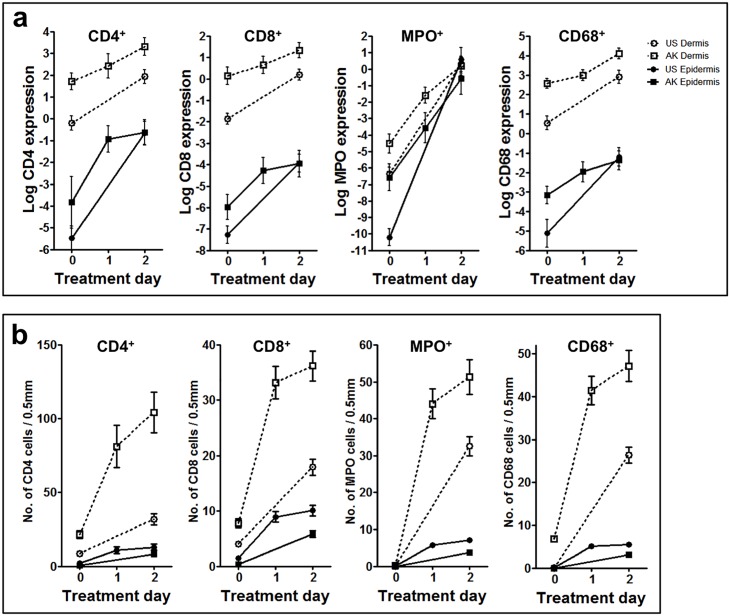
Cells of the adaptive and innate immune system increase strongly upon topical treatment with ingenol mebutate 0.05% gel. For detailed quantification, two complementary quantitative analyses were conducted to ensure high-quality measurements. Automated histomorphometric analyses **(A)** and manual counting of positive cells **(B)** were performed on all samples (actinic keratosis [AK] lesions at day 0, day 1, and day 2, and uninvolved skin (US) at day 0 and day 2 from all patients (*n* = 26). Depicted data represent averages +/- standard error of the mean. There was a statistically significant overall concordance with the two technologies, e.g. for CD4^+^ epidermis (r = 0.4) and dermis (r = 0.4) and for CD8^+^ epidermis (r = 0.5) and dermis (r = 0.7). The data for the computer-based histomorphometrical analysis were logarithmically transformed. Statistical significance was determined by analysis of variance for: CD4^+^ T-lymphocytes, epidermis/dermis AK0 versus AK2 (*P* = 0.0007/*P* = 0.0006), US0 versus US2 (*P* < 0.0001/*P* < 0.0001) and for US2 versus AK2 (not significant [NS]/*P* < 0.0001), CD8^+^ T-lymphocytes, epidermis/dermis AK0 versus AK2 (*P* = 0.0024/*P* = 0.0019), US0 versus US2 (*P* < 0.0001/*P* < 0.0001), and for US2 versus AK2 (NS/*P* = 0.0065), CD68^+^ macrophages/histiocytes, dermis (epidermis was NS) AK0 versus AK2 (*P* < 0.0001), US0 versus US2 (*P* < 0.0001), and for US2 versus AK2 (*P* = 0.0042), and MPO^+^ neutrophils, epidermis/dermis AK0 versus AK2 (*P* < 0.0001/*P* < 0.0001), US0 versus US2 (*P* < 0.0001/*P* < 0.0001), and for US2 versus AK2 (NS/NS). A full statistical overview is available in S1 Table.

To address the biological effects of ingenol mebutate in AK lesions and uninvolved skin, respectively, five biopsy specimens from each patient (from untreated AK lesions and uninvolved skin, AK lesions treated with ingenol mebutate for 1 or 2 days, and uninvolved skin treated with ingenol mebutate for 2 days) were assessed histopathologically and immunohistochemically (Figs 2 and 3). To ensure high-quality data, samples were subjected to three extensive, independent, and blinded analyses employing complementary methods: computer-based histomorphometric analyses, manually counting, and evaluation on a semiquantitative scoring scale from 0 to 3 of positive cells. The latter method had low sensitivity, though a adequate correlation with the two other methods was found (r = 0.4–0.7, *P*<0.05; Fig 4A and 4B).

**Fig 5:**
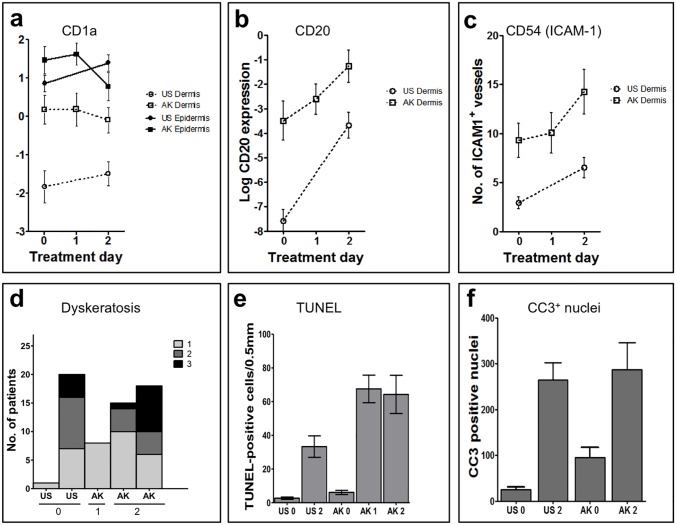
Topical ingenol mebutate 0.05% gel activates cutaneous blood vessels, attracts B-cells, and induces epidermal cell death. Automated histomorphometric analyses on all samples from all patients (*n* = 26) were performed to quantitate expression of CD1a^+^ dendritic cells **(A)**, CD20^+^ B-cells **(B)**, and CD54 (ICAM-1) expressing cutaneous blood vessels and inflammatory cells within the dermis **(C)**. Expression of both CD20 and CD54 increased rapidly and strongly upon treatment with ingenol mebutate, and actinic keratosis (AK) lesions showed generally higher expression levels compared with uninvolved skin. **(D)** Dyskeratotic (dead) keratinocytes within the epidermis of all samples (*n* = 26 patients; five biopsy specimens each) were assessed using a scoring system ranging from 0 (absent, not depicted), 1 (few dyskeratotic cells), 2 (several dead cells) to 3 (numerous necrotic cells). No necrosis was evident in the dermis. TdT-mediated dUTP-biotin nick end labeling (TUNEL)^+^ apoptotic cells **(E)** and cleaved caspase 3 (CC3)^+^ nuclei **(F)** were determined in biopsies from five patients. A full statistical overview is available in S1 Table.

Ingenol mebutate-induced immune responses were more pronounced in AK lesions compared with uninvolved skin. Although there were some between-patient differences, a marked focal leukocytic infiltrate accompanied by profound focal epidermal dyskeratosis and apoptotic cells was detected in both AK lesions and uninvolved skin (Figs 2–5). When the underlying immune cell subsets (granulocytes, macrophages, lymphocytes, Langerhans cells, mast cells) in each of the five biopsies from each patient were assessed, inflammatory cells, dominated by CD4^+^ and CD8^+^ T-lymphocytes as well as neutrophils (expressing myeloperoxidase, MPO) and macrophages (expressing CD68), were abundantly present within both dermis and epidermis as early as 1 day after treatment initiation (Fig 2). Likewise, the biopsies taken after 2 days of treatment showed a profound inflammatory response in both uninvolved skin and AK lesions (Fig 2). Detailed quantitation of each single cell type revealed a distinctly more pronounced ingenol mebutate-induced reaction pattern in AK lesions compared with uninvolved skin (Fig 4). As determined by immunohistochemistry, the density of CD4^+^ T-cells in the dermis was significantly higher in ingenol mebutate-treated AK lesions than in ingenol mebutate-treated uninvolved skin (difference 2.37-fold, *P*<0.0001; Fig 4). Albeit at somewhat lower overall numbers, similar patterns were determined with CD8^+^ T-cells (2.14-fold increase in AK lesions versus uninvolved skin, *P* = 0.0065; Fig 4), neutrophilic granulocytes (2-fold increase, *P*<0.0001; Fig 4), and macrophages (2.16-fold increase, *P* = 0.0042; Fig 4). Less abundant, but still markedly induced by ingenol mebutate with a preference in AK lesions, were dermal CD20^+^ B-cells (Figs 3 and 5). Epidermal Langerhans cells (expressing CD1a) did not show significant differences between ingenol mebutate-treated AK lesions and uninvolved skin. However, dermal CD1a^+^ cells were significantly more abundant in AK lesions compared with uninvolved skin after identical treatment (2.63-fold increase, *P* = 0.0006; Figs 3 and 5a). As detected by chloroacetate esterase staining, mast cells were not different in ingenol mebutate-treated AK and uninvolved skin.

The notion that ingenol mebutate induced a significantly more prolonged (Fig 1b) and intense inflammatory reaction pattern in AK-fields compared with uninvolved skin (Figs 2–5) was strongly supported by the concordance of the histomorphometric analyses and the “manual” evaluation, as depicted for CD4^+^ cells in epidermis (r = 0.4) and dermis (r = 0.4) and for CD8^+^ cells in epidermis (r = 0.5) and dermis (r = 0.7; Fig 4; S1A and S1B Table). Accompanying the inflammatory infiltrate, vascular endothelial cells were activated by ingenol mebutate, as detected by immunohistochemical staining of ICAM-1 (CD54). Again, the ingenol mebutate-induced ICAM-1 expression was clearly more pronounced in AK lesions compared with uninvolved skin (2.04-fold increase, *P* = 0.0139; Fig 5c).

### Cell death in AK lesions

Ingenol mebutate-induced cell death was restricted to the epidermis and occurred preferentially in AK lesions. While epidermal apoptosis and dyskeratosis (necrosis) as well as the inflammatory infiltrate were observed in all treated skin samples, these changes, again, were conspicuously more pronounced in AK lesions compared with uninvolved skin (Figs 4 and 5). Indeed, quantitative analysis of TdT-mediated dUTP-biotin nick end labeling (TUNEL) positive cells [34] in five consecutive patients (Figs 3 and 5E) revealed that the apoptotic response in treated AK lesions was significantly stronger compared with uninvolved skin (*P*<0.05). Furthermore, staining for activated (cleaved) caspase-3 in all 26 patients (Figs 3 and 5F) revealed an ingenol mebutate-induced effect. In addition, when expression of cleaved caspase-3 and TUNEL reactivity were compared in the skin samples, we found a convincing statistical correlation between the two independent parameters of apoptosis (Spearman correlation 0.8026, *P* = 0.0447).

Overall, AK lesions reacted significantly more strongly to ingenol mebutate compared with uninvolved skin.

### mRNA and miRNA expression

**Fig 6:**
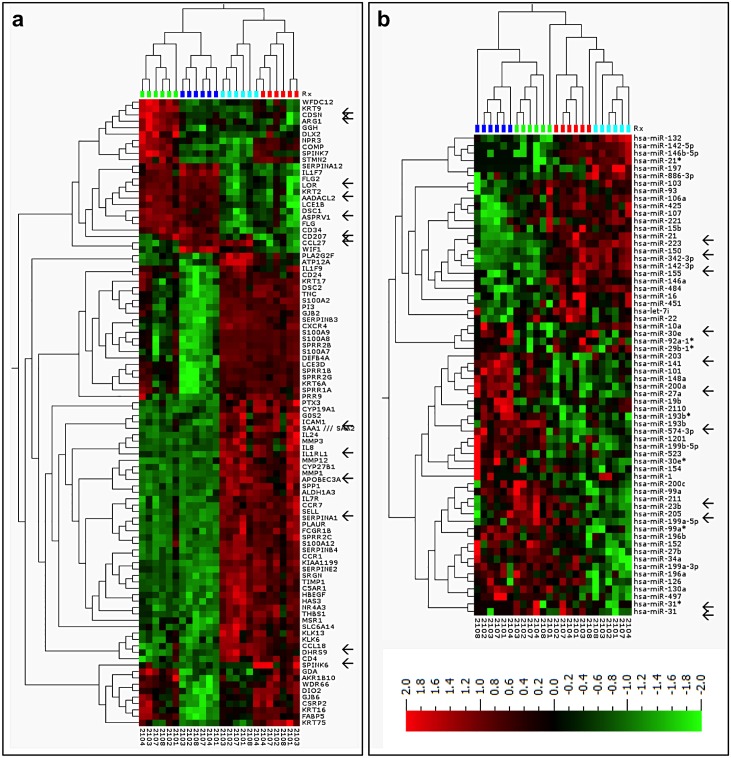
Topical treatment with ingenol mebutate 0.05% gel affects expression of gene clusters relevant for inflammatory and wound healing responses, and induces a characteristic microRNA (miRNA) pattern. The profiles illustrated are heat-maps and 2-way hierarchical clusterings of deregulated genes of **(A)** the 95 most variable mRNAs, and **(B)** the 64 most variable miRNAs across samples (*n* = 24) for the first six patients in the study. Differentially expressed genes and miRNAs were identified by pair-wise comparison of the following groups: actinic keratosis day 0 (AK0) versus uninvolved skin day 0 (US0), AK2 versus AK0, US2 versus US0, and AK2 versus US2. The expression analysis included variance filtering (VAR >0.2), statistical significance test by analysis of variance (*P* < 0.01), expression cut-off (>2-fold), and the false-discovery-rate was controlled by the Benjamini-Hochberg procedure (q < 0.05). Microarray data can be found in the GEO repository (GSE63107). Arrows indicate mRNA genes and miRNAs that were selected for validation by quantitative polymerase chain reaction. The colors above the heat map indicate: US0 (dark blue, *n* = 6), US2 (light blue, *n* = 6), AK0 (green, *n* = 6), and AK2 (red, *n* = 6) samples, respectively. The red and green shades on the heatmap represent up- and down-regulated genes, respectively.

The level of mRNA and miRNA expression in uninvolved skin and AK lesion biopsies before and after treatment with ingenol mebutate was assessed by a global microarray (mRNA) or QPCR-panel (miRNA) analysis in the first seven patients. Ingenol mebutate induced gene clusters for inflammatory, wound healing responses, and epidermal development. The global mRNA analysis (28,869 genes) showed, for the first time, that in untreated AK lesions (baseline) the gene expression profiles differed markedly from those of untreated uninvolved skin: 1,162 differentially expressed genes (DEG) were identified (variance >0.2, *P*<0.01) of which 972 were up- and 190 down-regulated in AK lesions (Fig 6A). furthermore, a functional annotation of the DEG indicated a skin-signature including keratinization, epidermis development and keratinocyte differentiation (Fig 6A). Interestingly, the key pathway emphasized in AK *versus* uninvolved skin was TGFβ1 (S2 Fig), consistent with the nature of AK being carcinoma *in situ*. TGFβ1 itself, however, was not changed as detected by mRNA expression.

Ingenol mebutate treatment affected the expression of numerous genes (cut-off >2-fold change, *P*<0.01). In uninvolved skin, 1,198 genes were up- and 318 down-regulated, whereas in AK lesions, 515 genes were up- and 346 down-regulated. Functional annotation of the up-regulated genes showed, that the main changes were inflammatory responses, response to wounding, and wound healing (Fig 6A, S3 Fig). The down-regulated genes revealed a skin-signature involved in keratinization and development of the epidermis (Fig 6a).

The global microarray data for 14 genes of interest (GOI) was validated by quantitative polymerase chain reaction (qPCR) and a statistically significant correlation was observed with the microarray data (*P* < 0.05). Further validation was done with the GOI in biopsies from all 26 patients. The level of five epidermis-specific genes (*CDSN*, *DSC1*, *FLG2*, *KRT2*, and *KRT9*) and of CD34 (angiogenesis-related) decreased after treatment with ingenol mebutate (*P*<0.05; S2 Table). Expression of MMP1 and four genes encoding for chemokines and adhesion proteins involved in recruiting immune cells to sites of inflammation, CXCL8, CCL18, ICAM-1, and SELL increased (*P*<0.05; Fig 6a; S2 Table).

### MicroRNA patterns in AK lesions and uninvolved skin

Micro-RNAs are small non-coding RNA molecules, which regulate the mRNA expression pattern through binding to the mRNA gene. Distinct microRNA patterns were induced by ingenol mebutate in AK lesions and uninvolved skin. Global miRNA qPCR-profiling (899 human miRNAs, *n* = 6) showed that in untreated AK lesions, 11 miRNAs differed from those of uninvolved skin, four being elevated and seven were diminished in AK lesions. Interestingly, comparing our data to recent miRNA-data in SCC [35], up-regulation of miR-21, miR-31, and down-regulation of miR-30e, miR-101 showed overlap between AK lesions only and SCC, representing the phenotype of AK being SCC.

After ingenol mebutate treatment, two miRNAs were up- and two down-regulated in AK lesions, whereas in uninvolved skin, 13 miRNAs were up- and 10 down-regulated (Fig 6b). Of those, eleven miRNAs of interest were validated by qPCR, and a statistically significant correlation was observed between the qPCR-profiling and qPCR-validation data for these miRNAs (median 0.7±0.26; *P*<0.05). In biopsies from all patients, at baseline miR-211, miR-31, miR-31* and miR-21 were up-regulated and miR-10a down-regulated in AK lesions compared with uninvolved skin. After treatment, in AK and in uninvolved skin, inflammatory associated miRNAs; miR-150 and miR-142-3p were up-regulated and the SCC oncogene miR-211 was down-regulated. In uninvolved skin, furthermore the inflammatory miRNAs; miR-31, and miR-21 were upregulated (S3 Table).

Overall, the mRNA and miRNA profiles did reflect an expression profile that both in AK lesions and uninvolved skin reflected inflammatory and healing signature and epidermal changes, towards normalization in AK, as measured by reduction in keratin expression. This suggests that the clinical LSR correspond (Fig 1) to the underlying molecular changes (Fig 6A and 6B, S3 Fig).

### *In vitro* validation of the multi-factorial pharmacodynamic profile observed in humans

Treating differentiated keratinocytes, proliferating keratinocytes and SCC cells with either low (150 nM) or high (250 μM) concentrations of IngMeb, it was evident that a higher TNFα and IL-8 release was detected in the supernatant when cells were treated with the lower concentration, and this was also clearly shown to be PKC-activation related (S4 Fig). Unexpectedly, when treating with the high concentrations of ingenol mebutate, the IL-8 and TNFα release was also strongly inhibited by a broad-spectrum PKC-inhibitor (S4 Fig). The TNFα release from SCC-cells were not significantly different both in untreated and ingenol mebutate treated cells.

## Discussion

The primary objective of the study was the first-in-man investigation of the biological and pharmacodynamic activity of ingenol mebutate, representing a novel class of anti-tumoral agents, in AK lesions (carcinoma *in situ* of the skin) and uninvolved skin.

Ingenol mebutate induced epidermal cell death as well as a strong inflammatory response in both uninvolved skin and AK lesions. Interestingly, when exact numbers of cells affected by ingenol mebutate were compared, a clear and statistically highly significant preference of AK lesions regarding numerous quantitative parameters (epidermal cell death, infiltration by several types of leukocytes) was established. Given that several inflammation-associated genes were expressed at elevated levels in untreated AK lesions compared with uninvolved skin, it is conceivable that this “pre-conditioning” of AK lesions contributed, at least in part, to the tumor-preferential activity of ingenol mebutate.

The 0.05% concentration of ingenol mebutate within the study medication reflects the amount of active pharmaceutical ingredient in the marketed product. A previous study has quantitated how much of the compound penetrates the skin [26] with high concentrations (up to 300 μM) being recovered in the stratum corneum and the epidermis, and lower concentrations (up to 1 μM) being recovered in the dermis. This reflects the proposed multi-modal mode-of-action.

Based on studies in rodents, both epidermal cell death and inflammation were expected [25, 36]. However, in contrast to mice, where ingenol mebutate field-directed treatment caused loss of the entire epidermis, which appeared to be followed by rapid re-epithelization from hair follicles [36], the cell death seen in this trial in humans affected almost exclusively the epidermis, leaving the basement membrane largely intact. This difference between mice and humans is likely due to the distinctly different skin structure and barrier function. Further, the strong cutaneous infiltration found within the papillary dermis, particularly of neutrophils, macrophages, and CD4/CD8-positive T-lymphocytes, contrasts with previous findings in mice, where in majority neutrophils [25] and induction of anticancer CD8 T-cell immunity has been reported after ingenol mebutate application [37]. The mechanistic basis for this unprecedented difference is currently unknown, and further highlights the necessity for human studies.

Our histomorphological finding that ingenol mebutate-induced cell death in humans was restricted to the epidermis now provides a plausible explanation for the findings from previous clinical trials showing that scarring is rarely, if at all, observed after treatment with ingenol mebutate [21]. In addition, scarring usually results from damage to the reticular dermis, and our results showing that inflammation was largely confined to the papillary dermis and the uppermost part of the reticular dermis with the lower dermis and the subcutaneous tissue unaffected, confirm this view. Furthermore, this finding may also explain that despite a potential development of clinically evident intense LSRs, all LSRs in this and in the previous clinical trials resolved without sequelae [21]. Evidence of improvements in skin texture 57 days post-ingenol mebutate treatment indicate certain positive aesthetic non-damaging dermal effects [20]. It is, therefore, anticipated that ingenol mebutate’s direct anti-neoplastic therapeutic activity is restricted to the immediate site of application and does not extend to deeper areas, thus minimizing unwanted side effects. Moreover, the multi-modal-mode-of-action being rapid induction of epidermal cell death and profound broad-spectra local immune responses by ingenol mebutate makes it unlikely that activation of resistance mechanisms will occur.

Apoptosis was induced by ingenol mebutate much more strongly in AK lesions than in uninvolved skin, and a strong association of TUNEL staining and cleaved-caspase-3 was found, confirming apoptosis with two different methods [38]. Since the atypical keratinocytes found in AK lesions arise from the basal cell layer [1], the ability to induce cell death in this compartment is likely to be important for the long-term clinical efficacy of ingenol mebutate on macroscopically visible AK lesions, as shown here, but also on subclinical non-visible AK lesions, explaining ingenol mebutate’s effect in the entire field treated. Furthermore, in the light of novel data showing that invasive SCC in most cases are overlaid by mild AK grades (KIN-I) [39], and the fact that ingenol mebutate induce cell death in the deeper compartment, may explain data that indicate reduced development of SCC in ingenol mebutate treated fields compared with lesion directed cryo-surgery in a field over a one year period [40]. Ingenol mebutate-induced cell death appears to be mediated through the PKCδ/MEK/ERK pathway [41].

The pronounced inflammation with preponderance in AK lesions after ingenol mebutate treatment may also contribute to the clinical efficacy. Neutrophils, which were markedly more abundant in intra-epidermal pustules of AK lesions after treatment, may contribute to cell death by releasing their reactive oxygen intermediates. In mouse models, neutrophils indeed contribute to anti-tumor responses and prevent relapse [29]. While CD4- and CD8-positive T-lymphocytes may contribute to immune responses against the transformed keratinocytes, the rapid onset of inflammation as early as 1 day after application suggests, that specific activation of naïve T-cells could not have taken place, at least not during the short observation period of our study. However, the fact that ingenol mebutate is a potent PKC activator [28, 30], and the fact that we did see significantly higher numbers of dendritic cells in the dermis in AKs, suggests that it activates cells of both the innate and adaptive immune system.

Furthermore, *in vitro* experiments indicated that the ingenol mebutate—induced chemokine release was dependent on the concentration used (150 nM vs. 250 μM) and probably PKC-activation even at high concentrations (250 μM), which confirms the role of ingenol mebutate in affecting the innate and adaptive immune system in a PKC-dependent fashion. This also suggests that the ingenol mebutate-caused direct cell death (which is accompanied by the release of cytokines and chemokines) is not the only effects that contributes to the inflammatory reaction clinically observed as a local skin reaction. Interestingly, the PKC-dependent TNFα secreted by proliferating keratinocytes might contribute to the killing of SCC cells.

The ingenol mebutate-induced up-regulation of ICAM-1 on vascular endothelium within the papillary dermis, a sign of activation and important for leukocyte extravasation, was much higher in AK lesions compared with uninvolved skin. Since ingenol mebutate can up-regulate ICAM-1 on human endothelial cells *in vitro* through specific activation of PKC [30], it is reasonable to assume that direct induction is at least part of its mode-of-action *in vivo*. In any case, up-regulated endothelial adhesion molecules may contribute to the influx of immune cells and, thus, to the overall anti-tumoral activity of ingenol mebutate. Neither damage nor hemorrhage were observed on or around dermal vessels, suggesting that in human skin ingenol mebutate doesn’t penetrate the dermis in toxic concentrations as reported previously [32].

Further support for an active immune response induction came from our gene expression profiling before and after ingenol mebutate treatment, which revealed an increase in the mRNA expression of four genes involved in recruiting immune cells to inflamed skin (CXCL8, CCL-18, ICAM-1, and SELL). Moreover, changes of miRNA expression following ingenol mebutate treatment are compatible with chronic inflammation [42–48], impaired epidermal differentiation [49, 50], or wound healing [51–53]. Thus, the overall mode of action appears to be trimodal: i) elicitation of a profound anti-tumoral immune response, ii) direct induction of epithelial cell death, and iii) stimulation of epithelial tissue regeneration necessary to replace demised tumor cells with normal keratinocytes.

In summary, we identified two basic and noteworthy reaction patterns of ingenol mebutate, induction of cell death and inflammation. Comparing AK and sun-shielded uninvolved skin, we identified a hitherto unreported outbalance of all assessed parameters in favor of AK lesions. We conclude that ingenol mebutate exerts a tumor-preferential activity driven by cell death and inflammation.

## Material and Methods

(Additional information available online as supplemental material)

### Study design

Ethics approval was granted by the Independent Ethics Committees (Bundesinstitut für Arzneimittel und Medizinprodukte Fachgebiet Klinische Prüfung/Inspektionen (Department of Clinical Trials/Inspections)) and the Institutional Ethics committee of the University Medical Center Göttingen. The clinical study protocol was conducted according to the Declaration of Helsinki (S1 and S2 Files). All patients received written and verbal information concerning the clinical trial and consented to their trial participation orally as well as in writing after a sufficient time to consider all relevant issues. A phase I, single-center, open-label, within-patient comparison trial to explore the biological effects of ingenol mebutate 0.05% gel, applied once daily on 2 consecutive days in patients with AK lesions on the upper extremity was performed (EudraCT Number 2011-001560-22). The trial was registered on the publicly available database www.clinicaltrials.gov under the identifier NCT01387711.

The trial population was planned to include 27 patients of ≥18 years of age with two to five clinically typical, visible, and discrete AK lesions within a contiguous 25-cm^2^ area (AK treatment area) on the upper extremity (dorsum of hands or forearm) and with one additional AK lesion located 1 to 5 cm from the AK treatment area. Further, a 25-cm^2^ area of uninvolved skin on the inner aspect of the upper arm was similarly treated with ingenol mebutate and biopsied. All eligible patients were to receive ingenol mebutate 0.05% gel on 2 consecutive days (days 1 and 2) to both the AK and uninvolved skin treatment areas (S1 Fig). Patients were recruited from February 1^st^ to March 30^th^, 2012, treated for two consecutive days, and followed for an additional 28 days.

Five 4-mm punch biopsies per patient were obtained. The one AK lesion outside the treatment area was biopsied at day 0 prior to the beginning of the treatment. Of the lesions within the treatment area, two were selected for biopsy during (day 1) and after (day 2) treatment. Biopsied sites were excluded from further treatment. The baseline biopsy of the uninvolved skin treatment area was taken within 1 to 5 cm away from the 25-cm^2^ treatment area. After 2 consecutive days of ingenol mebutate application, a second biopsy was taken within the selected treatment area on day 3. Two follow-up visits on days 8 and 29 were performed for clinical assessment of the treatment areas.

### Study assessments and data availability statement

The clinical skin responses were documented and assessed in a standardized manner.

To explore the biological effects following treatment with ingenol mebutate (primary study objective), the five biopsies per patient were subjected to standard histological examination and immunohistochemistry. All assessments were performed quantitatively in a blinded manner. For gene expression analyses, RNA was isolated from paraffin-embedded full thickness biopsies according to standard procedures and subjected to transcriptomics, mRNA, and miRNA analysis. Data were analyzed functionally and deposited to the GEO repository in accordance with MIAME guidelines (GSE63107).

### Statistical analysis

Study results were summarized into tabulations, listings, and figures where appropriate. There was no formal statistical hypothesis to be evaluated. SAS software, version 9.1.3, was applied for scores tabulated by visit and treatment area and paired analysis (number of patients with increased, unchanged, decreased score at day 3). Cell counts in histological sections were compared by student’s t-test, and *P*-values of <0.05 were considered statistically significant.

Histomorphometric data were log transformed in order to ensure a normal distribution prior to statistical analysis using a two-way analysis of variance for pairing all samples from a given patient. Where noted in the text, the statistical tests of the differences between samples of uninvolved skin (US) and AK lesions (AK) at day two were baseline-adjusted for differences between US0 and AK0.

## Supplementary Materials and Methods

### Additional information regarding the study design

The clinical study protocol received favorable opinion from the relevant Independent Ethics Committees (Bundesinstitut für Arzneimittel und Medizinprodukte Fachgebiet Klinische Prüfung/Inspektionen (Department of Clinical Trials/Inspections)) and was conducted according to the Declaration of Helsinki as adopted by the 18^th^ World Medical Assembly, 1964, and subsequent amendments as well as with the principles of Good Clinical Practice. All patients received written and verbal information concerning the clinical trial and consented to their trial participation orally as well as in writing after a sufficient time to consider all relevant issues. All patients agreed to allow photographs of the selected treatment areas to be taken and used in a publication.

Patients with lesions within 5 cm of an incompletely healed wound or infected skin area, and those with a history of skin conditions that might interfere with study evaluations, were excluded. In total, 28 patients were enrolled in our study and signed informed consent and 27 were treated with ingenol mebutate 0.05% gel within a 25-cm^2^ field containing AK lesions on the dorsum of their hands or forearms, and another 25-cm^2^ area of uninvolved, normal-appearing, aged but non-sun-exposed skin on the inner aspect of the upper arm once daily on 2 consecutive days. In order to obtain a precision corresponding to the half-width of the 95% confidence interval of 0.20 when the true response rate was 0.50 with more than 90% power, in total 27 subjects were required.

One patient was a screening failure and one patient was excluded from the analysis as the pathological diagnosis was stucco keratosis; in addition, one patient was excluded from the global RNA analysis due to inferior RNA quality.

### Additional information regarding study assessments

To compare the skin responses in uninvolved skin and AK lesions after treatment with ingenol mebutate gel 0.05% administered for 2 consecutive days (secondary study objective), photographs of the treatment areas were taken in a standardized manner at each visit, and the severity of the skin reaction was graded from 1 to 4 according to a 6-point local skin response (LSR) scale including erythema, flaking/scaling, crusting, swelling, vesiculation/pustulation, and erosion/ulceration.

Histological assessments were performed including cell death, inflammation/infiltration of leukocytes (all hematoxylin & eosin stain), standard immunohistochemistry methods markers for leukocyte subsets (CD4 [MT319], CD8 [DK25], CD20 [L26], CD68 [PGM1], CD1a [O10], myeloperoxidase [MPO-7]), and markers for vascular endothelium activation (ICAM-1 [6.5B5]) were assessed (all antibodies from Dako, Hamburg, Germany). Mast cells and neutrophilic granulocytes were visualized by chloroacetate esterase stainings. Apoptosis (days 0–3) was assessed using a modified terminal deoxy-nucleotidyl transferase-mediated dUTP nick end labeling (TUNEL) assay kit (DermaTACS; R&D Systems, Wiesbaden, Germany) and cleaved caspase 3 staining ([#9661] Cell Signaling Technology, Herlev, Denmark). For TUNEL staining [34], the tissue sections were digested with proteinase K (1 μg in 50 μL of DNase-free H_2_O), and the activity of endogenous peroxidase was quenched using 3% H_2_O_2_ in methanol. Incubation with terminal deoxynucleotidyl transferase (TdT) and the thymidine analog bromodesoxyuridine (BrdU) detected DNA fragmentation in individual apoptotic cells. The reaction was stopped by EDTA at a final concentration of 10 mM, and the cells were washed three times in phosphate-buffered saline. Incorporated BrdU was detected by a specific and sensitive biotin-conjugated anti-BrdU antibody in combination with a streptavidin—peroxidase complex. To quantitatively evaluate exact numbers of inflammatory cells within the skin, the entire sections were counted (10x or 20x magnification), and average cell counts per 0.5-mm skin were calculated for each tissue sample.

For histomorphometric analyses, all sections were scanned using a 20x magnification lens (Hamamatsu, Ballerup, Denmark) and image analyses were carried out using Visiomorph or Tissuemorph software (Visiopharm, Hørsholm, Denmark). Regions of interest covering the dermal and epidermal compartments were manually defined. The presence of CD1a, CD4, CD8, CD20, CD68, and myeloperoxidase-positive cells were evaluated by quantifying the total area occupied by the respective cell type followed by normalizing to the size of the biopsy. In principle, the cumulative stained areas were determined by creation of a multidimensional pseudocolor space emphasizing the characteristics of various picture elements. The pseudocolor space was analyzed next using a Bayesian-based classification method followed by extensive post-processing procedures optimized for each individual staining. The analyses were carried out by a person blinded to type of tissue and treatment.

The number of ICAM1^+^ vessels was determined using a fully automated approach based on classification of all vessel-like structures and ICAM1^+^ elements. Vessels surrounded by and in direct contact with ICAM1^+^ elements were defined as being an ICAM1^+^ vessel.

Cleaved caspase 3 (CC3)-positive cells were counted using Tissuemorph software (Visiopharm, Hørsholm, Denmark). The number of CC3^+^ cells was normalized to the total number of nuclei located in the basal layer of the epidermis, which was defined as ranging from the dermal-epidermal junction and 40 μm into the epidermis.

For gene expression analyses, the following chips were utilized: Affymetrix; GeneChip^®^ Human Gene U133 plus 2.0 microarray (14,500 human genes; Medical Prognosis Institute, Hoersholm, Denmark), miRCURY LNA^™^ Universal RT microRNA quantitative polymerase chain reaction (qPCR) profiling (742 human microRNAs and six reference genes; Exiqon A/S, Vedbaek, Denmark). Quantitative real-time PCR was used to validate findings (22 mRNAs and 22 miRNAs; AROS Applied Biotechnology AS, Science Park Skejby, Aarhus, Denmark). The expression analysis included variance filtering (VAR >0.2), statistical significance test by analysis of variance (*P* < 0.01), expression cut-off (>2-fold), and the false-discovery-rate was controlled by the Benjamini-Hochberg procedure (q < 0.05), to determine the differentially expressed genes (DEG) between sample conditions. For visualization, principal component analysis (PCA) heat maps and 2-way clustering were performed.

Functional analyses of microarray data were carried out to identify enriched annotations within disease and drug-induced biomarkers using the “*Database for Annotation*, *Visualization and Integrated Discovery”*, the “*Gene Ontology enRIchment anaLysis and visuaLizAtion tool”*, and “*Ingenuity Pathway Analysis*”.

### In vitro validation experiments

Proliferating HeKa, differentiated HeKa, (primary human keratinocytes, adult, HeKa, Invitrogen)) and HSC- 5 cells (human squamous cell carcinoma (Health Science Research Resources Bank, Japan) were treated with ingenol mebutate at high (250 μM) or low (150 nM) concentrations in the presence or absence of bisindolylmaleimide-1 (bis-1), an inhibitor of novel and classical PKC isoforms or DMSO (control).

HeKa were derived from three different adult donors and were grown in serum-free EpiLife medium with HKGS supplement (Invitrogen) containing 60 μM calcium and were used between passages 2 and 5. BSA (0.5%) was added to the medium during seeding of keratinocytes into multi-well plates on the day before the experiment in order to accustom cells to the presence of this protein. The HSC-5 were grown in Iscove's modified Dulbecco's medium. All media contained 10% fetal bovine serum and antibiotics. Cells were passaged after non-enzymatic detachment of the monolayers at 80–90% confluence. All cells were maintained in a humidified incubator at 37°C, 5% CO_2_. The Meso Scale Discovery immunoassay kit (Rockville, Maryland, USA) was used to detect IL-8 and TNFα. All experiments were carried out in triplicate.

## Supporting Information

S1 Fig Patient visits and procedures. Illustrated is a schematic overview of the trial design. The study included 7 visits per patient with procedures as indicated.
(PDF)

S2 Fig Actinic keratoses demonstrate up-regulation of genes in the tumor growth factor-β network. An up-stream network analysis of tumor growth factor (TGF**β**1) involved genes. Red shades indicate up-regulation of mRNAs, and green shades indicate down-regulation of mRNAs in actinic keratosis (AK) versus uninvolved-skin (US) at baseline.
(PDF)

S3 Fig Biological functions pathway analysis following ingenol mebutate gel treatment. The bar chart displays the most significant bio functions when comparing actinic keratosis (AK)2/AK0 (**A**) and uninvolved-skin (US)2/US0 (**B**). The significant values were calculated by Fisher’s exact test and indicate the probability of a given biological function. The higher the bars the more significant the respective function is. Functions are listed from most to least statistically significant. The orange horizontal line shows the cut-off for statistical significance (*P* < 0.05).
(PDF)

S4 Fig *In vitro* experiment of IL8 and TNFα release in proliferaring and differentiated keratinocytes, and SCC cells following ingenol mebutate gel treatment. The bars represents SEM. (**A**) the measured TNFα release (**B**) the measured IL-8 relaese.
(TIFF)

S1 File Clinical study protocol. (PDF)

S2 File TREND statement checklist. (DOCX)

S1 Table Statistical data of histomorphometric analyses. The analyses were carried out on logarithmically transformed data (and transformed back again, so the interpretations of the coefficients are multiplying factors of the original scale rather than differences on the log scale). **Day 0, actinic keratosis (AK) versus uninvolved-skin (US)**: two-sided analysis of variance with the factors patient and type (AK/NS). **AK or US, day 2 versus day 0**: two-sided analysis of variance with factors patient and day. **Day 2, AK versus NS**: two-sided analysis of variance with factors patient type (AK/NS).
(PDF)

S2 Table Validation of selected immune and epidermal related mRNAs in 26 patients. Untreated actinic keratosis (AK) lesions (AK0), AK-lesions treated with ingenol mebutate gel (IngMeb) for 1 (AK1) or 2 days (AK2), respectively, as well as uninvolved-skin without treatment (US0) or after 2 days of treatment with IngMeb (US2) were analyzed. The expression levels were normalized to minimally variable mRNAs GUSB and B2M. Dark green indicates a decrease in expression level of >2 ΔΔCt values comparing the median value (quantitative polymerase chain reaction [Q-PCR] data). Light green indicates a decrease in expression level of >1 ΔΔCt value comparing the median (qPCR data). Grey indicates differences in the median Ct values <1 ΔΔCt value. Dark red indicates an increase in expression level of >2 ΔΔCt values comparing the median (qPCR data). Orange indicates an increase in expression level of >1 ΔΔCt value comparing the median (qPCR data). Significance was tested with non-parametric Wilcoxon matched pair test. *P<0.05; **P < 0.01; ***P < 0.001.
(PDF)

S3 Table Validation of selected immune and epidermal function-related microRNAs (miRNAs) in 26 patients. The analyses were performed with untreated actinic keratosis (AK) lesions (AK0), AK-lesions treated with ingenol mebutate gel (IngMeb) for 1 (AK1) or 2 days (AK2), respectively, as well as with uninvolved-skin without treatment (US0) or after 2 days of treatment with IngMeb (US2). One minimally variable miRNA, namely *miR-99b*, was used for normalization. The median expression change (DCt) for each pair-wise comparison (AK0 versus US0, US2 versus US0, AK1 versus AK2, and AK2 versus AK0) is shown as well as its significance level (*P < 0.05; **P < 0.01; ***P < 0.001). Significance was tested with the non-parametric Wilcoxon matched pair test. The color coding indicates log2-fold up- (red >2 DCt, orange >1 DCt) or down- (dark green <-2 DCt) regulation. Grey shading indicates less than 2-fold (|DCt|<1) regulation.
(PDF)
