# Supplementary figures and images for: Tumor-Preferential Induction of Immune Responses and Epidermal Cell Death in Actinic Keratoses by Ingenol Mebutate
Source: PLoS One. 2016 Sep 9;11(9):e0160096. doi: 10.1371/journal.pone.0160096 (PMC5017628; doi:10.1371/journal.pone.0160096)

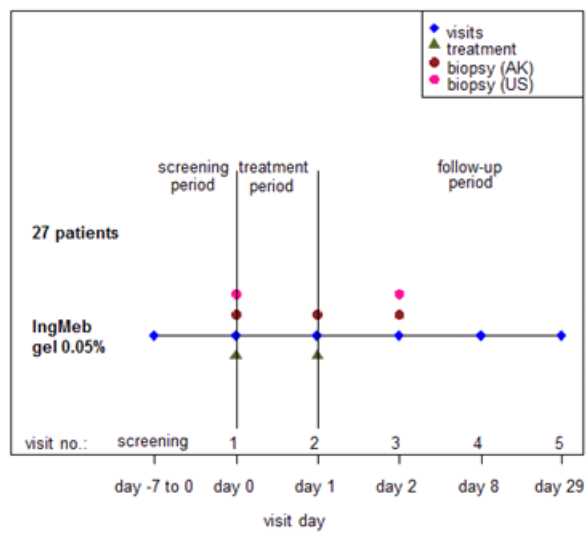

Supplement: S1 Fig — Illustrated is a schematic overview of the trial design. The study included 7 visits per patient with procedures as indicated. (PDF) [file pone.0160096.s001.pdf]

A

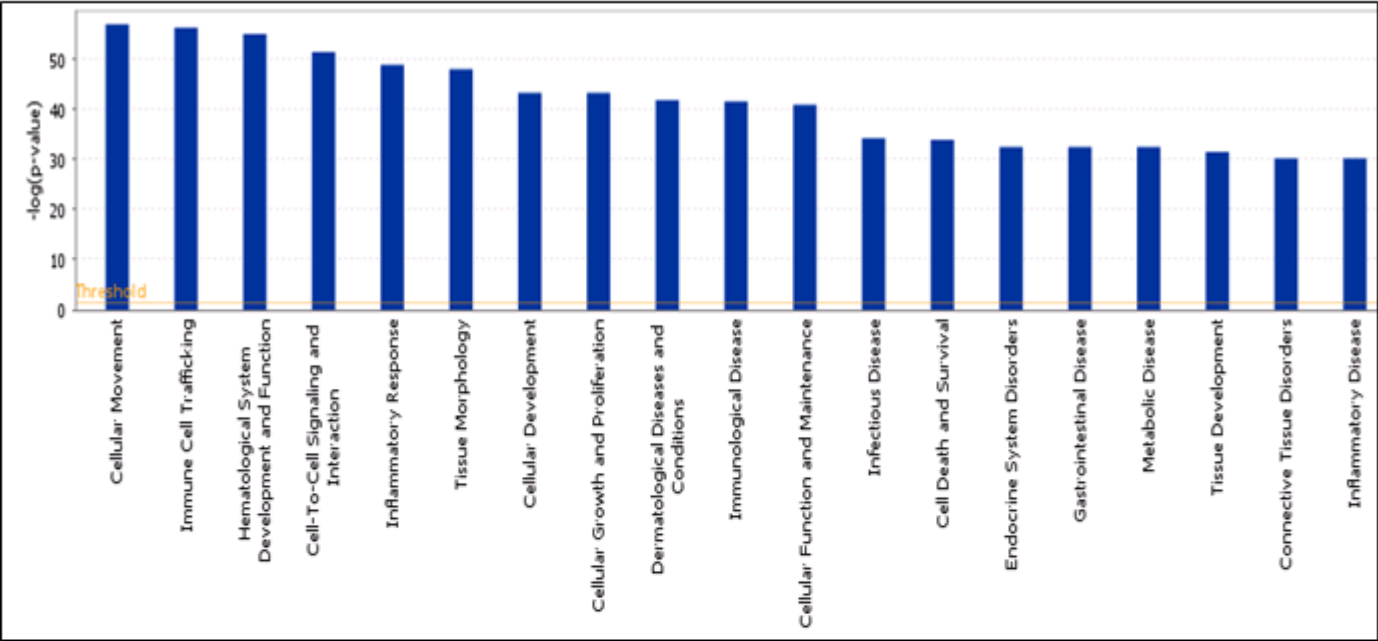

B

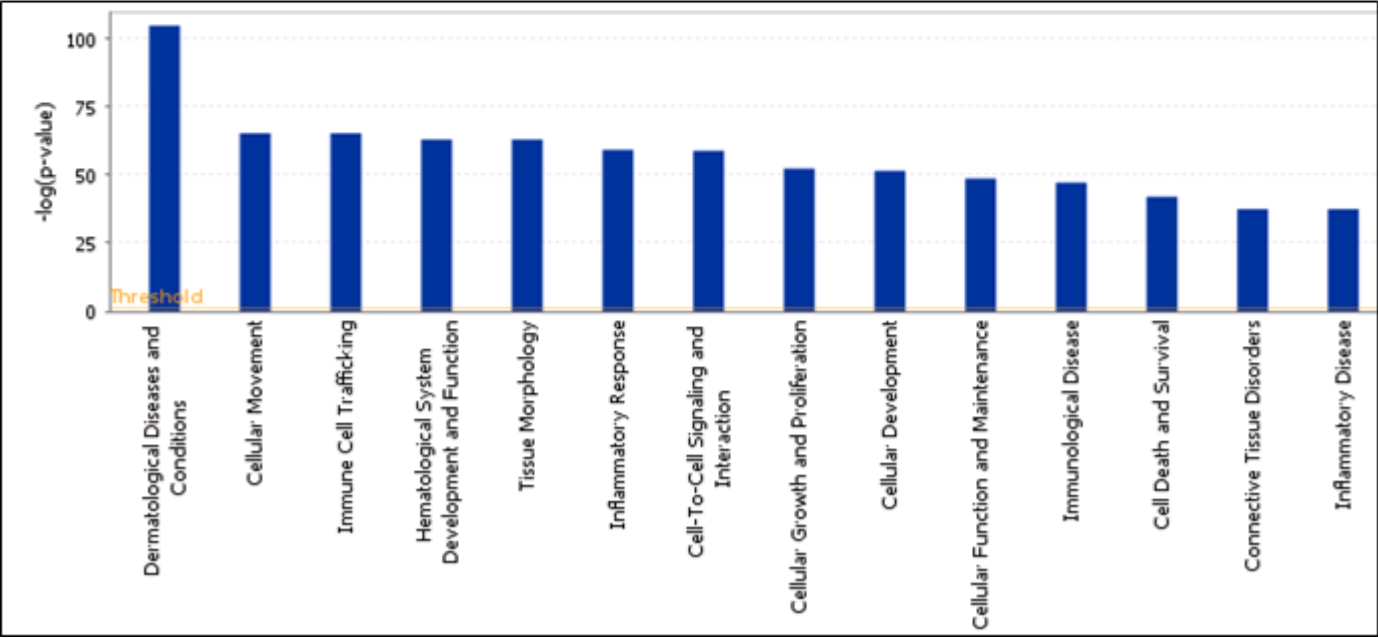

Supplement: S3 Fig — The bar chart displays the most significant bio functions when comparing actinic keratosis (AK)2/AK0 (A) and uninvolved-skin (US)2/US0 (B). The significant values were calculated by Fisher’s exact test and indicate the probability of a given biological function. The higher the bars the more significant the respective function is. Functions are listed from most to least statistically significant. The orange horizontal line shows the cut-off for statistical significance (P < 0.05). (PDF) [file pone.0160096.s003.pdf]

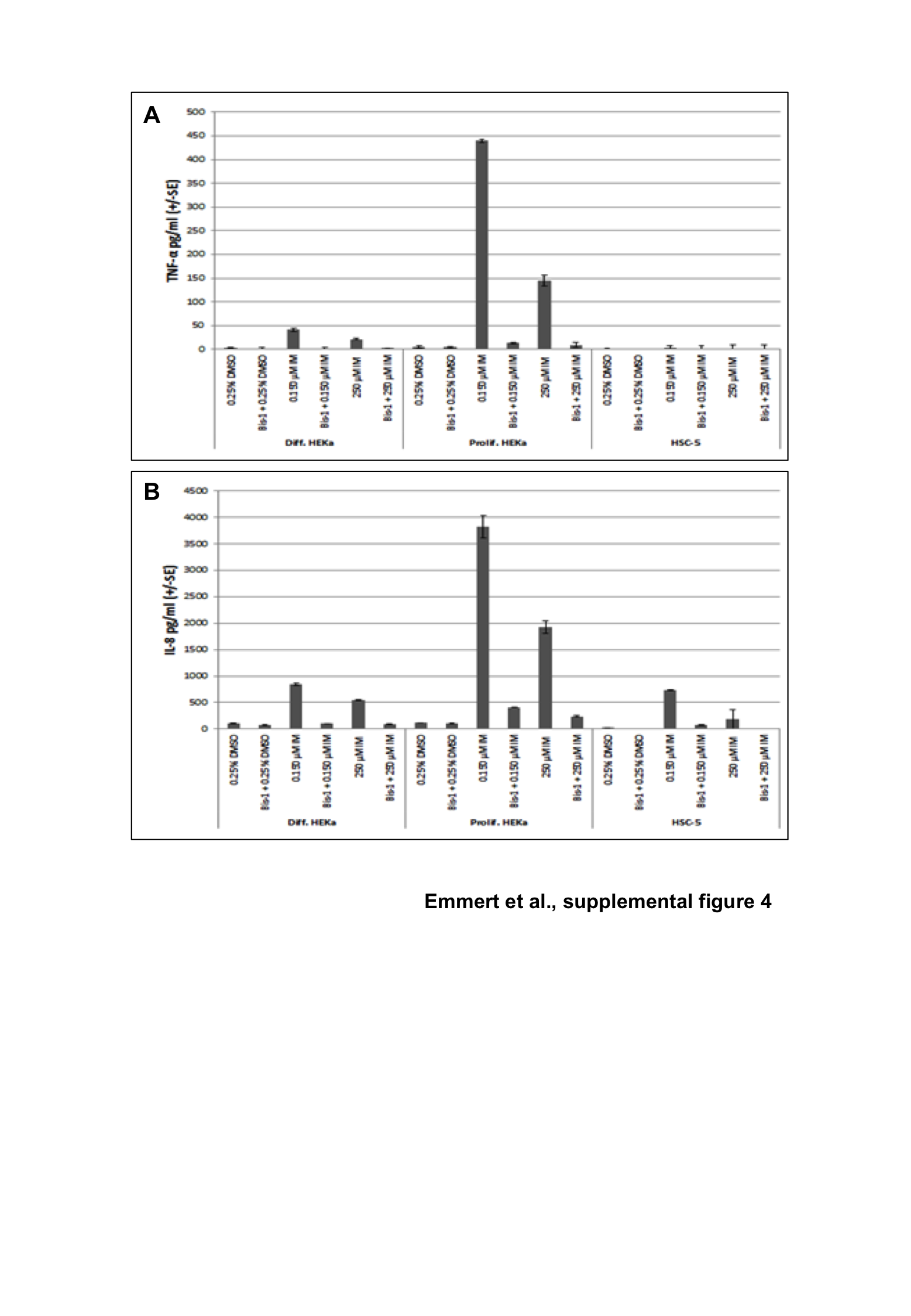

Supplement: S4 Fig — The bars represents SEM. (A) the measured TNFα release (B) the measured IL-8 relaese. (TIFF) [file pone.0160096.s004.tiff]
