# Supplementary material for: Tumor-Preferential Induction of Immune Responses and Epidermal Cell Death in Actinic Keratoses by Ingenol Mebutate
Source: PLoS One. 2016 Sep 9;11(9):e0160096. doi: 10.1371/journal.pone.0160096 (PMC5017628; doi:10.1371/journal.pone.0160096)
Supplement: S2 Fig — An up-stream network analysis of tumor growth factor (TGFβ1) involved genes. Red shades indicate up-regulation of mRNAs, and green shades indicate down-regulation of mRNAs in actinic keratosis (AK) versus uninvolved-skin (US) at baseline. (PDF) [file pone.0160096.s002.pdf]

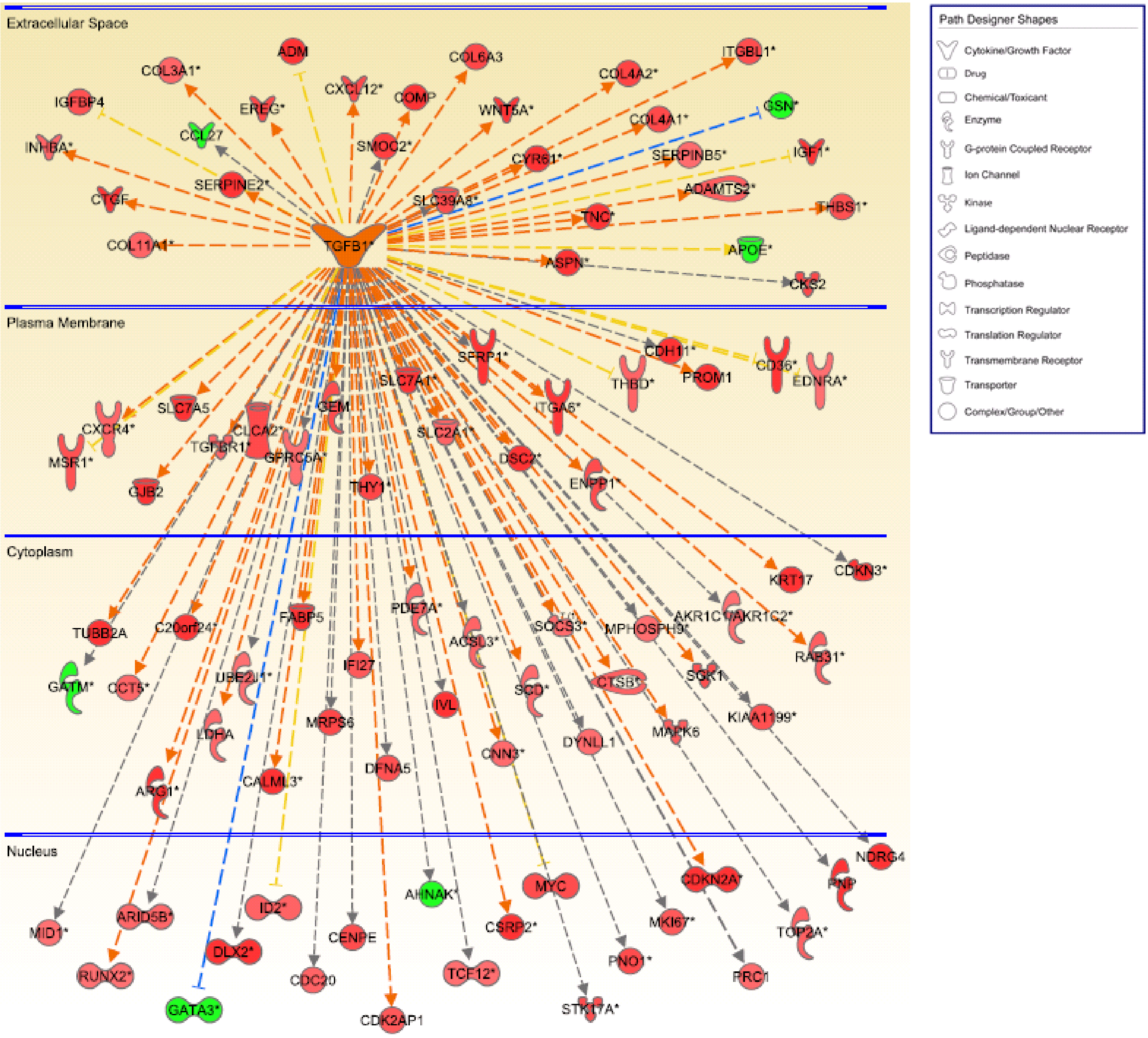

Extracellular Space
Plasma Membrane
Cytoplasm
Nucleus
TGFB1*
ADM
COL3A1*
COL6A3
ITGBL1*
CXCL12*
COMP
COL4A2*
IGFBP4
EREG*
WNT5A*
GSN*
CCL27
SMOC2*
COL4A1*
INHBA*
CYR61*
SERPINB5*
IGF1*
SERPINE2*
ADAMTS2*
SLC39A8*
CTGF
THBS1*
TNC*
COL11A1*
APOE*
ASPN*
CKS2
CDH11*
SFRP1*
CD36*
PROM1
THBD*
EDNRA*
SLC7A1*
SLC7A5
GEM
ITGA6*
CXCR4*
CLCA2*
SLC2A1*
TGFBR1*
GPRC5A*
DSC2*
MSR1*
ENPP1*
GJB2
THY1*
KRT17
CDKN3*
TUBB2A
C20orf24*
FABP5
PDE7A*
AKR1C1/AKR1C2*
SOCS3*
MPHOSPH9*
ACSL3*
RAB31*
GATM*
CCT5*
UBE2J1*
IFI27
SCD*
CTSB*
SGK1
IVL
LDHA
MRPS6
KIAA1199*
DYNLL1
MAPK6
CNN3*
DFNA5
ARG1*
CALML3*
NDRG4
PNP
CDKN2A*
MYC
AHNAK*
ID2*
ARID5B*
CSRP2*
MKI67*
TOP2A*
MID1*
CENPE
DLX2*
PNO1*
PRC1
RUNX2*
CDC20
TCF12*
GATA3*
STK17A*
CDK2AP1
Path Designer Shapes
Cytokine/Growth Factor
Drug
Chemical/Toxicant
Enzyme
G-protein Coupled Receptor
Ion Channel
Kinase
Ligand-dependent Nuclear Receptor
Peptidase
Phosphatase
Transcription Regulator
Translation Regulator
Transmembrane Receptor
Transporter
Complex/Group/Other
