# Supplementary material for: Tumor-Preferential Induction of Immune Responses and Epidermal Cell Death in Actinic Keratoses by Ingenol Mebutate
Source: PLoS One. 2016 Sep 9;11(9):e0160096. doi: 10.1371/journal.pone.0160096 (PMC5017628; doi:10.1371/journal.pone.0160096)
Supplement: S1 File — (PDF) [file pone.0160096.s005.pdf]

CONFIDENTIAL

Clinical Study Protocol

PEP005 Gel – Biological Effects in Actinic Keratosis assessed by histology

A phase I, single-centre, open label, within-subject comparison trial to explore the biological effects of PEP005 (ingenol mebutate) Gel, 0.05%, applied once daily for 2 consecutive days in patients with actinic keratosis on the upper extremity

ICH GCP statement: *The clinical trial will be conducted in compliance with the Clinical Study Protocol, GCP and the applicable regulatory requirement(s).*

**LEO Pharma A/S**	**Protocol Code Number:**	**LP0041-02**
**International Clinical Development**	**Date:**	**25May2011**
	**Version:**	**1.0**
	**EudraCT Number:**	**2011-001560-22**

L E O

THIS DOCUMENT CONTAINS TRADE SECRETS, OR COMMERCIAL OR FINANCIAL INFORMATION, PRIVILEGED OR CONFIDENTIAL, DELIVERED IN CONFIDENCE AND RELIANCE THAT SUCH INFORMATION WILL NOT BE COPIED OR MADE AVAILABLE TO ANY THIRD PARTY WITHOUT THE WRITTEN CONSENT OF LEO PHARMA A/S - LEO PHARMACEUTICAL PRODUCTS LTD. A/S

1 CLINICAL STUDY PROTOCOL APPROVAL/ACKNOWLEDGE

1.1 APPROVAL STATEMENT LEO PHARMA A/S

On behalf of LEO Pharma A/S, only the Head of International Clinical Development, and the Head of Biostatistics and Data Management, are authorised to approve the Clinical Study Protocol and Consolidated Clinical Study Protocol(s) comprising any subsequent amendment(s).

The following persons have approved this Clinical Study Protocol using electronic signatures as presented on the last page of this document:

Claus Bay
Head of Biostatistics and Data Management

Per Sprøgel
Head of International Clinical Development

1.2 APPROVAL STATEMENT INTERNATIONAL CO-ORDINATING INVESTIGATOR

It is the responsibility of the International Co-ordinating Investigator to approve the Clinical Study Protocol and Consolidated Clinical Study Protocol(s) comprising any subsequent amendment(s).

The following person has approved this Clinical Study Protocol by manually signing the International Co-ordinating Investigator Clinical Study Protocol Approval Form adjoined as a separate page to this document:

Prof. Dr. Michael P. Schön
International Co-ordinating Investigator

1.3 ACKNOWLEDGE STATEMENT INVESTIGATOR(S)

Each participating investigator must agree to the approved Clinical Study Protocol and Consolidated Clinical Study Protocol(s) comprising any subsequent amendment(s) by signing the Investigator (Consolidated) Clinical Study Protocol Agreement Form.

L E O
THIS DOCUMENT CONTAINS TRADE SECRETS, OR COMMERCIAL OR FINANCIAL INFORMATION, PRIVILEGED OR CONFIDENTIAL, DELIVERED IN CONFIDENCE AND RELIANCE THAT SUCH INFORMATION WILL NOT BE COPIED OR MADE AVAILABLE TO ANY THIRD PARTY WITHOUT THE WRITTEN CONSENT OF LEO PHARMA A/S - LEO PHARMACEUTICAL PRODUCTS LTD. A/S

2 PROTOCOL STATEMENT

2.1 COMPLIANCE WITH GOOD CLINICAL PRACTICE

This Clinical Study Protocol is designed to comply with the guideline produced by the International Conference on Harmonisation (ICH) on the topic Good Clinical Practice (GCP) as well as other relevant guidelines issued by ICH, primarily the efficacy guidelines.

3 PROTOCOL SYNOPSIS

**Name of finished/ investigational product:**	PEP005 Gel, 0.05%
**Name of active substance:**	Ingenol mebutate
**Title of trial/ protocol code number:**	Biological Effects in Actinic Keratosis assessed by histology / LP0041-02
**Co-ordinating investigator(s):**	Prof. Dr. Michael P. Schön
**Estimated number of trial sites and distribution:**	Single centre in Germany
**Trial period:**	First patient first visit: July 2011<br>Last patient last visit: February 2012
**Main objective(s):**	To explore the biological effects following treatment with PEP005 Gel, 0.05% administered for two consecutive days
**Methodology:**	This is a Phase I, single-centre, open label, within-subject comparison trial to explore the biological effects of PEP005 (ingenol mebutate) Gel, 0.05%, applied once daily for 2 consecutive days in patients with actinic keratosis on the upper extremity. For the purposes of this study the selected treatment areas for each patient will be as defined below:<br><br>1. "AK Treatment Area": A contiguous area of 25 cm2 of skin on the upper extremity (including the dorsum manus) that contains 2 to 5 Actinic Keratosis (AK) lesions. Additionally there must be at

THIS DOCUMENT CONTAINS TRADE SECRETS, OR COMMERCIAL OR FINANCIAL INFORMATION, PRIVILEGED OR CONFIDENTIAL, DELIVERED IN CONFIDENCE AND RELIANCE THAT SUCH INFORMATION WILL NOT BE COPIED OR MADE AVAILABLE TO ANY THIRD PARTY WITHOUT THE WRITTEN CONSENT OF LEO PHARMA A/S - LEO PHARMACEUTICAL PRODUCTS LTD. A/S

least one AK lesion located within 1 to 5 cm outside of the selected AK Treatment Area.

2. “Normal Skin Treatment Area”: A contiguous area of 25 cm2 of normal skin that has no or only minimal sun-damage from the inner upper arm. Note: the two selected treatment areas can be on the same or different arms.

All eligible subjects will receive PEP005 Gel, 0.05%, on each treatment area on Days 1 and 2. Study medication application will be (sub)investigator applied. Biopsies from the two selected treatment areas will be conducted at visits 1, 2, and 3.
Subsequent follow up visits will be made on Day 3, Day 8 and Day 29.

Number of subjects to be enrolled: A total of 27 patients will be enrolled into this study

Main criteria for inclusion:

1. Male or female patients at least 18 years of age
2. Patients with 2 to 5 clinically typical, visible and discrete AK lesions within a contiguous 25 cm^2 area (AK Treatment Area) on the upper extremity; with one additional AK lesion located 1 to 5 cm from the AK Treatment Area
3. Patients with a 25 cm^2 area of normal skin (Normal Skin Treatment Area) on the inner upper arm suitable for treatment and biopsy
4. Female subjects must be of either:
   - Non-childbearing potential[1], post-menopausal, or have a confirmed clinical history of sterility (e.g. the subject is without a uterus) or,
   - Childbearing potential, provided there is a confirmed negative urine pregnancy test pri-

[1] Female subjects are considered of childbearing potential unless they have been hysterectomised or have undergone tubal ligation or have been post-menopausal for at least one year prior to screening visit

L E O
THIS DOCUMENT CONTAINS TRADE SECRETS, OR COMMERCIAL OR FINANCIAL INFORMATION, PRIVILEGED OR CONFIDENTIAL, DELIVERED IN CONFIDENCE AND RELIANCE THAT SUCH INFORMATION WILL NOT BE COPIED OR MADE AVAILABLE TO ANY THIRD PARTY WITHOUT THE WRITTEN CONSENT OF LEO PHARMA A/S - LEO PHARMACEUTICAL PRODUCTS LTD. A/S

or to exposure, to rule out pregnancy.

5. Female subjects of childbearing potential must be willing to consent to using high effective methods of **contraception (Pearl index < 1%)** at study entry and for the duration of the trial participation. Adequate methods of contraception are defined as
   - abstinence (when this is in line with the preferred and usual lifestyle of the subject)
   - vasectomised partner (partner should be the sole partner for the subject)
   - an intrauterine device
   - double barrier method defined as two distinct methods (either two actual barrier methods or one actual barrier method and one hormonal method)
   - hormonal contraceptive (oral hormonal birth control, estrogenic vaginal ring, percutaneous contraceptive patches, implants and injectables) for at least one menstrual cycle prior to enrolment (Visit 1)
6. Ability to follow study instructions and likely to complete all study requirements
7. Obtained written informed consent prior to any study-related procedures
8. Agreement from the patient to allow photographs of the selected treatment area to be taken and used as part of the study data package

Main criteria for exclusion:

1. Location of the selected treatment areas:
   - Within 5 cm of an incompletely healed wound or infected area of the skin (excl. study related biopsies)
   - Within 10 cm of a suspected basal cell carcinoma (BCC) or squamous cell carcinoma (SCC)
2. History or evidence of skin conditions other than the study indication that would interfere with evaluation

L E O
THIS DOCUMENT CONTAINS TRADE SECRETS, OR COMMERCIAL OR FINANCIAL INFORMATION, PRIVILEGED OR CONFIDENTIAL, DELIVERED IN CONFIDENCE AND RELIANCE THAT SUCH INFORMATION WILL NOT BE COPIED OR MADE AVAILABLE TO ANY THIRD PARTY WITHOUT THE WRITTEN CONSENT OF LEO PHARMA A/S - LEO PHARMACEUTICAL PRODUCTS LTD. A/S

of the study medication (e.g. eczema, unstable psoriasis, xeroderma pigmentosum).

3. Prior treatment with PEP005 Gel
4. Treatment area lesions that have an atypical clinical appearance (e.g. hypertrophic, hyperkeratotic, recalcitrant disease [had cryosurgery on two previous occasions] and/or cutaneous horns).
5. Known sensitivity or allergy to any of the ingredients of PEP005 Gel (e.g. citric acid)
6. Clinical diagnosis/history or evidence of any medical condition that would expose a subject to an undue risk of a significant AE or interfere with assessments of safety during the course of the study, as determined by Investigator clinical judgment.
7. Anticipated need for in-patient hospitalisation or in-patient surgery during the study period. Note that cosmetic/therapeutic procedures are not excluded if they fall outside of the criteria detailed in the Prohibited Therapies or Medications (listed below).
8. Anticipated excessive or prolonged exposure to ultraviolet light (e.g. sunlight) or use of tanning beds for the duration of the study
9. Current participation in any other interventional clinical trial
10. Subjects who have received treatment with any **non-marketed drug product** (i.e. an agent which has not yet been made available for clinical use following registration) within the last two months.
11. Subject known or, in the opinion of the investigator, is **unlikely to comply** with the Clinical Study Protocol (e.g. alcoholism, drug dependency or psychotic state).
12. Females who are **pregnant**, of child-bearing potential and wishing to become pregnant during the trial, or are **breast feeding**.
13. Females of child-bearing potential with **positive**

THIS DOCUMENT CONTAINS TRADE SECRETS, OR COMMERCIAL OR FINANCIAL INFORMATION, PRIVILEGED OR CONFIDENTIAL, DELIVERED IN CONFIDENCE AND RELIANCE THAT SUCH INFORMATION WILL NOT BE COPIED OR MADE AVAILABLE TO ANY THIRD PARTY WITHOUT THE WRITTEN CONSENT OF LEO PHARMA A/S - LEO PHARMACEUTICAL PRODUCTS LTD. A/S

pregnancy test at [screening or visit 1].

14. Previous enrolment in this clinical trial.

Prohibited Therapies and/or Medications: **within 2 weeks prior to the Screening visit**

15. Cosmetic or therapeutic procedures (e.g. use of liquid nitrogen, surgical excision, curettage, dermabrasion, medium or greater depth chemical peel, laser resurfacing): within 2 cm of the selected treatment areas and within 2 cm of the selected AK lesion outside the treatment area
16. Use of acid-containing therapeutic products (e.g. salicylic acid or fruit acids, such as alpha and beta hydroxy acids and glycolic acids), topical retinoids or light chemical peels: within 2 cm of the selected treatment areas and within 2 cm of the selected AK lesion outside the treatment area
17. Use of topical salves (non-medicated/non-irritant lotion/cream are acceptable) or topical steroids: within 2 cm of the selected treatment areas and within 2 cm of the selected AK lesion outside the treatment area; artificial tanners: within 5 cm of the selected treatment areas and within 5 cm of the selected AK lesion outside the treatment area

Prohibited Therapies and/or Medications: **within 4 weeks prior to** the Screening visit

18. Treatment with immunomodulators (e.g. azathioprine), cytotoxic drugs (e.g. cyclophosphamide, vinblastine, chlorambucil, methotrexate, podophyllin, camptothecin) or interferon/interferon inducers.
19. Treatment with systemic medications that suppress the immune system (e.g. cyclosporine, prednisone, methotrexate, alefacept, infliximab).
20. Treatment/therapy with UVB

L E O

THIS DOCUMENT CONTAINS TRADE SECRETS, OR COMMERCIAL OR FINANCIAL INFORMATION, PRIVILEGED OR CONFIDENTIAL, DELIVERED IN CONFIDENCE AND RELIANCE THAT SUCH INFORMATION WILL NOT BE COPIED OR MADE AVAILABLE TO ANY THIRD PARTY WITHOUT THE WRITTEN CONSENT OF LEO PHARMA A/S - LEO PHARMACEUTICAL PRODUCTS LTD. A/S

	Prohibited Therapies and/or Medications: **within 8 weeks prior to the Screening visit**<br>21. Treatment with 5-FU, imiquimod, diclofenac, or photodynamic therapy: within 2 cm of the selected treatment areas and within 2 cm of the selected AK lesion outside the treatment area<br><br>Prohibited Therapies and/or Medications: **within 6 months prior to the Screening visit**<br>22. Use of systemic retinoids (e.g. isotretinoin, acitretin, bexarotene)
**Investigational product:**	PEP005 (ingenol mebutate) Gel, 0.05%, supplied in a kit of four single use, unit tubes for topical application.
**Reference product:**	No reference product is used in this protocol.
**Duration of treatment:**	All eligible subjects will receive PEP005 Gel, 0.05%, on two consecutive Days (Day 1 and Day 2) to both the AK Treatment Area and Normal Skin Treatment Area. The expected duration of subject participation is up to 36 days (including 7 days screening prior to first exposure, 2 days of exposure, and an additional 27 days follow up).
**Assessments:**	No study related procedures will be conducted prior to informed consent being obtained. At the screening visit subject demography, medical history, concomitant diagnosis/concomitant medication will be recorded. Clinical Assessments to be conducted for this study are listed below.<br>• Local Skin Response (LSR): LSRs of both selected treatment areas will be assessed at all visits using the LSR Grading Scale provided (Appendix V). The presence/absence and grading of the following responses will be recorded; erythema, flaking/scaling, crusting, swelling, vesiculation/postulation and erosion/ulceration. Skin responses other than these, should be recorded as

L E O

THIS DOCUMENT CONTAINS TRADE SECRETS, OR COMMERCIAL OR FINANCIAL INFORMATION, PRIVILEGED OR CONFIDENTIAL, DELIVERED IN CONFIDENCE AND RELIANCE THAT SUCH INFORMATION WILL NOT BE COPIED OR MADE AVAILABLE TO ANY THIRD PARTY WITHOUT THE WRITTEN CONSENT OF LEO PHARMA A/S - LEO PHARMACEUTICAL PRODUCTS LTD. A/S

AEs accordingly.

- Physical Examination will be conducted at the Screening and Visit 1 only.
- Photography: Photographs of the two selected treatment areas will be taken at all visits.
- Vital Signs including blood pressure (diastolic and systolic), heart rate and body temperature will be measured at all visits.
- Urine Pregnancy Test will be conducted at the screening, Visits 1 and 4 for female of child bearing potential.

Histological Assessments will be conducted on biopsy samples. Biopsies to be taken for this study are listed below.

- Visit 1 (Day 1): 4 mm punch biopsies from one AK lesion and from Normal skin (1 to 5 cm outside the selected treatment areas) prior to any study medication application (baseline biopsy)
- Visit 2 (Day 2): 4 mm punch biopsy from one AK lesion within the selected AK Treatment Area prior to Day 2 study medication application
- Visit 3 (Day 3): 4 mm punch biopsies from one AK lesion and from Normal skin (within the selected treatment areas)

Efficacy evaluation: Not applicable

Primary end point/ response criterion: The response criterion is:

- Degree of skin infiltration of leukocytes in haematoxylin & eosin-stained sections assessed on a scale from 0 to 3 in the AK biopsies from baseline, Day 2, and Day 3
- Degree of necrosis of the epidermis and dermis in haematoxylin & eosin-stained sections assessed as binary outcome (present/absent) in the AK biopsies from baseline, Day 2, and Day 3

L E O

THIS DOCUMENT CONTAINS TRADE SECRETS, OR COMMERCIAL OR FINANCIAL INFORMATION, PRIVILEGED OR CONFIDENTIAL, DELIVERED IN CONFIDENCE AND RELIANCE THAT SUCH INFORMATION WILL NOT BE COPIED OR MADE AVAILABLE TO ANY THIRD PARTY WITHOUT THE WRITTEN CONSENT OF LEO PHARMA A/S - LEO PHARMACEUTICAL PRODUCTS LTD. A/S

Secondary end point/ response criterion:

1. Degree of skin infiltration of leukocytes in haematoxylin & eosin-stained sections assessed on a scale from 0 to 3 in the normal skin biopsies from day 3 compared to baseline and to AK lesion biopsies
2. Degree of necrosis of the epidermis and dermis in haematoxylin & eosin-stained sections assessed as binary outcome (present/absent) in the normal skin biopsies from day 3 compared to baseline and to AK lesion biopsies
3. Degree of haemorrhage assessed by histology (binary outcome present/absent) in the Normal skin and AK lesion biopsies at all time points
4. Characterisation of immune cells such as T and B lymphocytes, polymorphonuclear cells, mast cells and antigen presenting cells in the Normal skin and AK lesion biopsies
5. Degree of apoptosis in the Normal skin and AK lesion biopsies
6. Degree of vascular endothelium activation in the Normal skin and AK lesion biopsies
7. Changes in mRNA and miRNA expression in the Normal skin and AK lesion biopsies
8. Expression of drug transporters in the Normal skin and AK lesion biopsies (P-gP)

Safety evaluation:

Reported adverse events and their seriousness, intensity and causality will be assessed for the safety evaluation.

Evaluation of vital signs, reasons for withdrawal and withdrawal rate as well as Local Skin Response (LSR) will also be recorded.

L E O
THIS DOCUMENT CONTAINS TRADE SECRETS, OR COMMERCIAL OR FINANCIAL INFORMATION, PRIVILEGED OR CONFIDENTIAL, DELIVERED IN CONFIDENCE AND RELIANCE THAT SUCH INFORMATION WILL NOT BE COPIED OR MADE AVAILABLE TO ANY THIRD PARTY WITHOUT THE WRITTEN CONSENT OF LEO PHARMA A/S - LEO PHARMACEUTICAL PRODUCTS LTD. A/S

Statistical methods:

Prior to database lock, a final detailed statistical analysis plan (SAP) will be available. Study results will be summarised into tabulations, listings and figures where appropriate.

Histological summaries will be based on the Intent-to-Treat (ITT) population, which is defined as all patients that have received at least one dose of study medication and have a post-baseline assessment.

Safety analyses will be based on the safety population, which is defined as all patients that have received at least one dose of study medication and have at least one post-baseline safety assessment.

Statistical Hypothesis

Twenty-seven patients should be enrolled into this study to obtain an adequate precision. There is no formal statistical hypothesis to be evaluated.

The primary objective is to explore the biological effects of PEP005 in the skin. The degree of skin infiltration of leukocytes and the degree of necrosis of the epidermis and dermis will be presented in the AK biopsies at Day 1, 2 and 3 graphically and in tabular format.

The secondary objective is to compare the skin response between normal and AK treatment areas within subjects. Data will be presented graphically and as cross-tables comparing the distribution of the endpoints assessed at the normal skin and AK treated areas.

L E O

THIS DOCUMENT CONTAINS TRADE SECRETS, OR COMMERCIAL OR FINANCIAL INFORMATION, PRIVILEGED OR CONFIDENTIAL, DELIVERED IN CONFIDENCE AND RELIANCE THAT SUCH INFORMATION WILL NOT BE COPIED OR MADE AVAILABLE TO ANY THIRD PARTY WITHOUT THE WRITTEN CONSENT OF LEO PHARMA A/S - LEO PHARMACEUTICAL PRODUCTS LTD. A/S

3.1 SCHEDULE/CHART OF TRIAL PROCEDURES

	Screening	Visit 1	Visit 2	Visit 3	Visit 4	Visit 5
Day	-7 to 1	1	2	3	8 Follow up	29<br>End of Trial
**Visit window**					+/- 3 days	+/- 3 days
Informed consent	X					
Subject demographics	X					
In-/exclusion criteria	X	X				
Urine Pregnancy test[1)]	X	X			X	
Concomitant diagnoses	X					
Concomitant medication	X	X	X	X	X	X
Medical history	X	X				
Physical examination	X	X				
Biopsy		X	X[2)]	X		
Vital signs (BP, heart rate in supine position, oral body temperature)	X	X	X	X	X	X
LSR	X	X	X	X	X	X
Administration of trial medication by physician		X	X[3)]			
Photographs	X	X	X	X	X	X
Adverse Event(s)		X	X	X	X	X
End of Trial Form						X[4)]

1) For female of child-bearing potential a urine pregnancy test must be performed at the screening visit
2) Only on AK lesion within AK Treatment Area
3) Study medication application to be applied within 24 +/- 2 hours where possible. If subjects prematurely withdrawn from study medication every attempt should be made to have the subject attend all follow up visits.
4) If the subject is not enrolled the End of Trial form in eCRF should be completed

L E O
THIS DOCUMENT CONTAINS TRADE SECRETS, OR COMMERCIAL OR FINANCIAL INFORMATION, PRIVILEGED OR CONFIDENTIAL, DELIVERED IN CONFIDENCE AND RELIANCE THAT SUCH INFORMATION WILL NOT BE COPIED OR MADE AVAILABLE TO ANY THIRD PARTY WITHOUT THE WRITTEN CONSENT OF LEO PHARMA A/S - LEO PHARMACEUTICAL PRODUCTS LTD. A/S

TABLE OF CONTENTS

1 CLINICAL STUDY PROTOCOL APPROVAL/ACKNOWLEDGE 2
1.1 APPROVAL STATEMENT LEO PHARMA A/S 2
1.2 APPROVAL STATEMENT INTERNATIONAL CO-ORDINATING INVESTIGATOR 2
1.3 ACKNOWLEDGE STATEMENT INVESTIGATOR(S) 2
2 PROTOCOL STATEMENT 3
2.1 COMPLIANCE WITH GOOD CLINICAL PRACTICE 3
3 PROTOCOL SYNOPSIS 3
3.1 SCHEDULE/CHART OF TRIAL PROCEDURES 12
TABLE OF CONTENTS 13
4 LIST OF ABBREVIATIONS AND DEFINITION OF TERMS 17
4.1 LIST OF ABBREVIATIONS 17
4.2 DEFINITION OF TERMS 18
5 ETHICS 21
5.1 INDEPENDENT ETHICS COMMITTEES (IECS) OR INSTITUTIONAL REVIEW BOARDS (IRBS) 21
5.2 ETHICAL CONDUCT OF THE TRIAL 21
5.3 ETHICAL CONSIDERATION STATEMENT 21
5.4 SUBJECT INFORMATION AND INFORMED CONSENT 22
5.5 HANDLING OF PERSONAL DATA 23
6 TRIAL ADMINISTRATIVE STRUCTURE 23
6.1 SPONSOR 23
6.2 LEO PHARMA A/S AFFILIATES AND CRO(S) 23
6.2.1 LEO Pharma A/S affiliate(s) 23
6.2.2 Contract Research Organisation(s) (CRO(s)) 23
6.3 LEO PHARMA A/S PERSONNEL 24
6.3.1 International Clinical Trial Manager (ICTM) 24
6.3.2 National Clinical Trial Manager (NCTM) 24
6.3.3 Sponsor's Medical Expert 24
6.3.4 Safety Scientist, Global Pharmacovigilance 24
6.3.5 Study Statistician 24
6.3.6 Study Data Manager 24
6.3.7 Pre-clinical Specialist 24

L E O

THIS DOCUMENT CONTAINS TRADE SECRETS, OR COMMERCIAL OR FINANCIAL INFORMATION, PRIVILEGED OR CONFIDENTIAL, DELIVERED IN CONFIDENCE AND RELIANCE THAT SUCH INFORMATION WILL NOT BE COPIED OR MADE AVAILABLE TO ANY THIRD PARTY WITHOUT THE WRITTEN CONSENT OF LEO PHARMA A/S - LEO PHARMACEUTICAL PRODUCTS LTD. A/S

6.4 PROTOCOL AUTHOR(S) 25
6.5 INVESTIGATORS AND TRIAL COMMITTEES 26
6.5.1 International Co-ordinating Investigator 26
6.5.2 National Co-ordinating Investigators 26
6.5.3 Investigators 26
6.5.4 Trial Committees 26
6.6 AGREEMENTS 27
7 INSURANCE 27
8 INTRODUCTION AND RATIONALE 27
8.1 ACTINIC KERATOSIS 27
8.2 INVESTIGATIONAL PRODUCT DESCRIPTION 28
8.3 TRIAL RATIONALE 28
8.3.1 Necrotic Cell Death 29
8.3.2 Immune Modulation 29
9 TRIAL OBJECTIVES 31
9.1 PRIMARY OBJECTIVE 31
9.1.1 Primary Outcome Measures 31
9.2 SECONDARY OBJECTIVES 31
9.2.1 Secondary Outcome Measures 31
10 INVESTIGATIONAL PLAN 32
10.1 TRIAL DESIGN 32
10.1.1 Overall Design 32
10.1.2 Individual Periods 33
10.1.2.1 Screening Period (Day -7 to Day 1) 33
10.1.2.2 Treatment Period (Day 1, Day 2) 34
10.1.2.3 Follow up Period: Day 3, Day 8, Day 29 35
10.1.3 Overall Chart 36
10.2 TIME SCHEDULE 37
10.3 NUMBER OF SUBJECTS/SAMPLE SIZE 37
10.4 CRITERIA FOR SUBJECT SELECTION (IN- AND EXCLUSION) 37
10.4.1 Inclusion Criteria 37
10.4.2 Exclusion Criteria 38
10.4.3 Subject Screening Log 40
10.4.4 Subject Registration 40
10.5 WITHDRAWAL CRITERIA 40
10.6 INVESTIGATIONAL PRODUCTS 41

L E O
THIS DOCUMENT CONTAINS TRADE SECRETS, OR COMMERCIAL OR FINANCIAL INFORMATION, PRIVILEGED OR CONFIDENTIAL, DELIVERED IN CONFIDENCE AND RELIANCE THAT SUCH INFORMATION WILL NOT BE COPIED OR MADE AVAILABLE TO ANY THIRD PARTY WITHOUT THE WRITTEN CONSENT OF LEO PHARMA A/S - LEO PHARMACEUTICAL PRODUCTS LTD. A/S

10.6.1 Packaging of Investigational Products 42
10.6.2 Storage of Investigational Products 42
10.6.3 Administration of Investigational Products 42
10.6.4 Precautions/Overdosage 43
10.6.5 Treatment Assignment 44
10.6.5.1 Randomisation Code List 45
10.6.5.2 Subject Identification List 45
10.6.6 Blinding of the Trial 45
10.6.7 Breaking the Randomisation Code 45
10.6.8 Drug Accountability and Compliance Checks 45
10.6.8.1 Sponsor-Investigator Drug Accountability 45
10.6.8.2 Investigator-Subject Drug Accountability 46
10.6.8.3 End of Trial Drug Accountability 46
10.6.8.4 Treatment Compliance 46
10.6.9 Prior and Concomitant Treatment 46
10.6.9.1 Prior to the Trial 46
10.6.9.2 During the Trial 46
10.7 TRIAL PROCEDURES 48
10.7.1 Schedule of Trial Procedures 48
10.7.2 Subject Eligibility 49
10.7.3 Clinical Assessment 49
10.7.3.1 Investigator Assessments 49
10.7.3.2 Subject assessments 51
10.7.3.3 Imaging Assessments 51
10.7.4 Laboratory Assessments 51
10.7.4.1 Central Analysis 51
10.7.4.2 Local Analysis 53
10.7.5 Adverse Events 53
10.7.5.1 Reporting of Adverse Events 54
10.7.5.2 Other events to be reported 57
10.7.6 Serious adverse events 57
10.7.6.1 Reporting of Serious Adverse Events 57
10.7.7 Source Data 58
10.8 EFFICACY EVALUATION 59
10.9 SAFETY EVALUATION 60
10.9.1 Evaluation of (Serious) Adverse Events 60

L E O

THIS DOCUMENT CONTAINS TRADE SECRETS, OR COMMERCIAL OR FINANCIAL INFORMATION, PRIVILEGED OR CONFIDENTIAL, DELIVERED IN CONFIDENCE AND RELIANCE THAT SUCH INFORMATION WILL NOT BE COPIED OR MADE AVAILABLE TO ANY THIRD PARTY WITHOUT THE WRITTEN CONSENT OF LEO PHARMA A/S - LEO PHARMACEUTICAL PRODUCTS LTD. A/S

10.9.2 Evaluation of Laboratory Data ... 60
10.9.3 Evaluation of Other Observations ... 60
10.10 STATISTICAL ANALYSIS ... 61
10.11 TRIAL COMMITTEES ... 63
10.12 QUALITY ASSURANCE/AUDIT ... 63
10.12.1 Trial Monitoring ... 63
10.13 CASE REPORT FORM BOOKS AND DATA HANDLING ... 64
10.13.1 Case Report Forms (CRFs) ... 64
10.13.2 Data Handling ... 65
10.14 PROTOCOL AMENDMENTS ... 65
10.15 COMPLETION OF TRIAL ... 66
10.15.1 Trial Completion Procedures ... 66
10.15.2 Provision for Subject Care Following Trial Completion ... 66
10.15.3 Archiving of Trial Documents ... 66
11 USE OF INFORMATION ... 67
12 PUBLICATION ... 67
13 REFERENCES ... 69
14 LIST OF APPENDICES ... 73

L E O

THIS DOCUMENT CONTAINS TRADE SECRETS, OR COMMERCIAL OR FINANCIAL INFORMATION, PRIVILEGED OR CONFIDENTIAL, DELIVERED IN CONFIDENCE AND RELIANCE THAT SUCH INFORMATION WILL NOT BE COPIED OR MADE AVAILABLE TO ANY THIRD PARTY WITHOUT THE WRITTEN CONSENT OF LEO PHARMA A/S - LEO PHARMACEUTICAL PRODUCTS LTD. A/S

4 LIST OF ABBREVIATIONS AND DEFINITION OF TERMS

4.1 LIST OF ABBREVIATIONS

5-FU	5-Fluorouracil
ADR	Adverse Drug Reaction
AE	Adverse Event
AK	Actinic Keratosis
BCC	Basal Cell Carcinoma
BP	Blood Pressure
CD	Cluster of Differentiation
CMO	Contract Manufacturing Organisation
CRF	Case Report Form
CRO	Contract Research Organisation
eCRF	Electronic Case Report Form
EMA	European Medicines Agency
EU	European Union
FDA	Food and Drug Administration
GCP	Good Clinical Practice
GPV	Global Pharmacovigilance
HEENT	Head, Eye, Ear, Nose and Throat
ICH	International Conference on Harmonisation
ICTM	International Clinical Trial Manager
IEC	Independent Ethics Committee
IP	Investigational Product
IRB	Institutional Review Board
ITT	Intent-to-Treat
LSR	Local Skin Response
MedDRA	Medical Dictionary for Regulatory Activities
mi-RNA	Micro Ribonucleic Acid
mRNA	Messenger Ribonucleic Acid

L E O
THIS DOCUMENT CONTAINS TRADE SECRETS, OR COMMERCIAL OR FINANCIAL INFORMATION, PRIVILEGED OR CONFIDENTIAL, DELIVERED IN CONFIDENCE AND RELIANCE THAT SUCH INFORMATION WILL NOT BE COPIED OR MADE AVAILABLE TO ANY THIRD PARTY WITHOUT THE WRITTEN CONSENT OF LEO PHARMA A/S - LEO PHARMACEUTICAL PRODUCTS LTD. A/S

NCTM	National Clinical Trial Manager
NMSC	Non-Melanoma Skin Cancer
P-gP	P-glycoprotein
PCR	Polymerase Chain Reaction
PKC	Protein Kinase C
PUVA	Psoralen Ultra Violet A
RDC	Remote Data Capture
SAE	Serious Adverse Event
SAP	Statistical Analysis Plan
SCC	Squamous Cell Carcinoma
SDV	Source Data Verification
SmPC	Summary of Product Characteristics
SOC	System Organ Class
SOP	Standard Operating Procedure
SUSAR	Suspected Unexpected Serious Adverse Reaction
TEAE	Treatment Emergent Adverse Events
UVB	Ultra Violet B
WMA	World Medical Assembly

4.2 DEFINITION OF TERMS

Assessment
A (cluster of) characteristic(s) measured and/or recorded for a subject.

Certified Copy
A certified copy is a copy of original information that has been verified, as indicated by a dated signature, as an exact copy having all of the same attributes and information as the original (FDA Guidance for Industry, Computerized Systems Used in Clinical Investigations, May 2007).

L E O
THIS DOCUMENT CONTAINS TRADE SECRETS, OR COMMERCIAL OR FINANCIAL INFORMATION, PRIVILEGED OR CONFIDENTIAL, DELIVERED IN CONFIDENCE AND RELIANCE THAT SUCH INFORMATION WILL NOT BE COPIED OR MADE AVAILABLE TO ANY THIRD PARTY WITHOUT THE WRITTEN CONSENT OF LEO PHARMA A/S - LEO PHARMACEUTICAL PRODUCTS LTD. A/S

Concomitant Medication
Any medication taken by a subject during the clinical trial apart from the investigational product.

Enrolled Subject
A subject for whom informed consent has been obtained and a CRF number assigned.

Fraud
Fabrication of data, selective and undisclosed rejection of undesired results, substitution with fictitious data, deliberately incorrect use of statistical methods for the purposes of reaching other conclusions than those warranted by the data, misinterpretation of results and conclusions, plagiarism of results or entire articles from other researchers, misrepresentation of other researchers' results, unwarranted authorship, and misleading application for positions or funds.

International Clinical Trial Manager (ICTM)
The person appointed by LEO Pharma A/S to be the main international representative responsible for all aspects of a clinical trial as outlined in International Clinical Development SOPs.

Clinical trial agreement
A contract between on the one hand LEO Pharma A/S and/or a Contract Research Organisation (CRO) and on the other hand an investigator and/or the institution specifying the conditions for the co-operation in the clinical trial and the investigator's and/or the institution's responsibilities.

Investigator Staff Signature Form
A form used:

23. for the investigator to delegate trial related tasks/duties
24. for trial site staff to sign and date to accept delegation
25. for trial site staff to document signature and initials
26. for the investigator to authorise tasks/duties delegated.

L E O
THIS DOCUMENT CONTAINS TRADE SECRETS, OR COMMERCIAL OR FINANCIAL INFORMATION, PRIVILEGED OR CONFIDENTIAL, DELIVERED IN CONFIDENCE AND RELIANCE THAT SUCH INFORMATION WILL NOT BE COPIED OR MADE AVAILABLE TO ANY THIRD PARTY WITHOUT THE WRITTEN CONSENT OF LEO PHARMA A/S - LEO PHARMACEUTICAL PRODUCTS LTD. A/S

Investigator Trial File
The collection of trial documents required by International Clinical Development SOPs LEO Pharma A/S, ICH Guidelines and/or regulatory requirements to be on file at the trial site.

LEO Pharma A/S
LEO Pharma A/S refers to the sponsor of the clinical trial.

LEO Pharma A/S affiliate
An affiliated company of LEO Pharma A/S authorised to manage certain clinical trial related activities for LEO Pharma A/S.

Monitor
A person appointed by LEO Pharma A/S to carry out monitoring of a clinical trial.

National Clinical Trial Manager (NCTM)
The person appointed by LEO Pharma A/S to be the national representative responsible for all aspects of a clinical trial within a country as outlined in International Clinical Development SOPs.

Response Criterion
An assessment or a transformation of the assessment(s) described on a subject level, for which a statistical analysis is performed, i.e. a p-value or a confidence interval is stated, or for which tabulation serves as important supportive evidence of efficacy/safety.

Subject Identification List
A summary list kept by the investigator in the Investigator Trial File which records the names of all subjects enrolled and the date of enrolment in the trial at that trial site. The list includes each subject’s corresponding CRF Number to allow the investigator/institution to reveal the identity of any subject, if required.

Subject Screening Log
A document kept by the investigator which identifies patients/subjects who entered pre-trial screening.

L E O
THIS DOCUMENT CONTAINS TRADE SECRETS, OR COMMERCIAL OR FINANCIAL INFORMATION, PRIVILEGED OR CONFIDENTIAL, DELIVERED IN CONFIDENCE AND RELIANCE THAT SUCH INFORMATION WILL NOT BE COPIED OR MADE AVAILABLE TO ANY THIRD PARTY WITHOUT THE WRITTEN CONSENT OF LEO PHARMA A/S - LEO PHARMACEUTICAL PRODUCTS LTD. A/S

Subject Screening Log is synonymous with Patient Screening Log.

Subject Study Card
A card given to a subject by the trial site at the time trial medication is first dispensed to a subject, to identify that the subject is having treatment with an investigational product.

Writing committee
An appointed committee participating in the writing of a multi-centre publication.

5 ETHICS

5.1 INDEPENDENT ETHICS COMMITTEES (IECs) OR INSTITUTIONAL REVIEW BOARDS (IRBs)

The clinical trial must be approved by/receive favourable opinion from relevant Institutional Review Boards (IRBs)/ Independent Ethics Committees (IECs) prior to enrolment of subjects.

Any amendments to the approved clinical trial must likewise, as required, be approved by/receive favourable opinion from relevant IRBs/IECs prior to implementation.

The appropriate regulatory authority(ies) must be notified of/approve the clinical trial, as required.

5.2 ETHICAL CONDUCT OF THE TRIAL

This clinical trial will be conducted to conform to the principles of the World Medical Association (WMA), Declaration of Helsinki, Ethical Principles for Medical Research Involving Human Subjects, adopted by the 18th WMA General Assembly, Helsinki, Finland, June 1964, and last revised in Seoul in October 2008 by the WMA General Assembly (see Appendix II).

5.3 ETHICAL CONSIDERATION STATEMENT

Actinic Keratosis (AK) is a common skin condition which is linked to the development of squamous cell carcinoma (SCC) (1). Current treatment options for AK lesions consist of cryotherapy, photodynamic therapy, and topical products. Photodynamic therapy and cryotherapy can be painful, and patients are often left with hypopigmented spotting where

L E O

THIS DOCUMENT CONTAINS TRADE SECRETS, OR COMMERCIAL OR FINANCIAL INFORMATION, PRIVILEGED OR CONFIDENTIAL, DELIVERED IN CONFIDENCE AND RELIANCE THAT SUCH INFORMATION WILL NOT BE COPIED OR MADE AVAILABLE TO ANY THIRD PARTY WITHOUT THE WRITTEN CONSENT OF LEO PHARMA A/S - LEO PHARMACEUTICAL PRODUCTS LTD. A/S

cryotherapy is applied (2, 3). Curettage (with or without electrosurgery) and excisional surgery are alternatives to cryosurgery (4). Topical products include 5-fluorouracil (5-FU), diclofenac, and imiquimod, and are commonly used as field treatment for multiple lesions over larger skin areas (5, 6, 7, 8, 9, 10, 11, 12, 13). A new topical product, PEP005 Gel, which contains the active ingredient ingenol mebutate is being developed for the treatment of actinic keratosis. The duration of treatment with PEP005 Gel is 2 to 3 days which provides an advance for treatment compliance and patient convenience.

The safety of PEP005 Gel has been established across numerous clinical studies. Localised application site disorders (e.g., pruritus, pain, irritation) and local skin responses (LSRs), particularly erythema, flaking, and scaling are the main characteristics of the safety profile of PEP005 Gel. These local adverse events are transient, and typically resolve without sequelae within 2-4 weeks of application. Furthermore, these local events do not have a tendency to become infected and do not cause scarring. PEP005 Gel has no detectable systemic absorption at concentrations applied topically which are effective for complete clearance of AK lesions.

Subjects participating in the study will be under careful supervision of an experienced investigator during the entire course of the study. All the test procedures will be performed at the study centre. In case of a local skin response, the affected skin areas will be closely monitored to minimise the discomfort, if any, for the subject. If any unacceptable reactions are observed, further PEP005 Gel exposures will be withheld; however the subject will be followed for safety.

The information obtained in this study about the biological effects of ingenol mebutate will provide an important link to the biological effects observed in animal models. This understanding will be valuable for the further development of ingenol mebutate for the treatment of skin cancer as well as other indications, for optimising new formulations of ingenol mebutate and for the selection of second generation drugs with more improved efficacy and tolerability.

5.4 SUBJECT INFORMATION AND INFORMED CONSENT

All subjects shall receive written and verbal information concerning the clinical trial. This information will emphasise that participation in the clinical trial is voluntary and that the subject may withdraw from the clinical trial at any time and for any reason. All subjects will be given opportunity to ask questions and will be given sufficient time to consider before consenting.

LEO

THIS DOCUMENT CONTAINS TRADE SECRETS, OR COMMERCIAL OR FINANCIAL INFORMATION, PRIVILEGED OR CONFIDENTIAL, DELIVERED IN CONFIDENCE AND RELIANCE THAT SUCH INFORMATION WILL NOT BE COPIED OR MADE AVAILABLE TO ANY THIRD PARTY WITHOUT THE WRITTEN CONSENT OF LEO PHARMA A/S - LEO PHARMACEUTICAL PRODUCTS LTD. A/S

The subject's signed and dated informed consent to participate in the clinical trial must be obtained **prior** to any clinical trial related procedure being carried out.

A Study Subject Card will be given to a subject by the site personnel at the time of first drug exposure to identify that the subject is having treatment with an investigational product.

5.5 HANDLING OF PERSONAL DATA

Subjects, shall be asked to consent that their personal data are recorded, collected, processed and may be transferred to EU and non-EU countries, in accordance with any national legislation regulating privacy and data protection.

Personal data shall be handled and processed by all relevant parties involved in the clinical trial in accordance with any national legislation regulating privacy and data protection as well as in accordance with the general terms and conditions of the authorisation granted by the Danish Data Protection Agency to LEO Pharma A/S as set forth in the attached Appendix I. LEO Pharma A/S is considered data controller for this clinical trial.

6 TRIAL ADMINISTRATIVE STRUCTURE

6.1 SPONSOR

LEO Pharma A/S is the sponsor of the clinical trial.

6.2 LEO PHARMA A/S AFFILIATES AND CRO(S)

LEO Pharma AS has transferred certain clinical trial related activities to the LEO Pharma A/S affiliate(s) and/or to the CRO(s) relevant for the conduct of the clinical trial.

6.2.1 LEO Pharma A/S affiliate(s)

LEO Pharma GmbH, Frankfurter Straße 233, A3, D-63263 Neu-Isenburg, Germany

6.2.2 Contract Research Organisation(s) (CRO(s))

Almac Clinical Services, 4204 Technology Drive, Durham, North Carolina, United States will be responsible for drug storage, packaging, labelling, distribution and destruction of investigational product (IP) as agreed to in a Service Agreement/Contract.

L E O

THIS DOCUMENT CONTAINS TRADE SECRETS, OR COMMERCIAL OR FINANCIAL INFORMATION, PRIVILEGED OR CONFIDENTIAL, DELIVERED IN CONFIDENCE AND RELIANCE THAT SUCH INFORMATION WILL NOT BE COPIED OR MADE AVAILABLE TO ANY THIRD PARTY WITHOUT THE WRITTEN CONSENT OF LEO PHARMA A/S - LEO PHARMACEUTICAL PRODUCTS LTD. A/S

6.3 LEO PHARMA A/S PERSONNEL

6.3.1 International Clinical Trial Manager (ICTM)

Julia Delfs, Dipl. oec. troph., International Clinical Trial Manager, Clinical Operations, LEO Pharma GmbH, Frankfurter Straße 233, A3, D-63263 Neu-Isenburg, Germany, Tel.: +49 6102 201 113, Fax: +49 6102 201 100, e-mail: julia.delfs@leo-pharma.com

6.3.2 National Clinical Trial Manager (NCTM)

Kathrin Riepe, National Clinical Trial Manager, Clinical Operations, LEO Pharma GmbH, Frankfurter Straße 233, A3, D-63263 Neu-Isenburg, Germany, Tel.: +49 6102 201 102, Fax: +49 6102 201 100, e-mail: kathrin.riepe@leo-pharma.com

6.3.3 Sponsor's Medical Expert

Torsten Skov, Senior Medical Advisor, LEO Pharma A/S, Industriparken 55, 2750 Ballerup, Denmark, Tel.: +45 72 26 39 90, Fax: +45 72 26 33 21, Mobile: +45 20 73 62 94, e-mail: torsten.skov@leo-pharma.com

6.3.4 Safety Scientist, Global Pharmacovigilance

Bjarke Naver, MSc PhD, Principal PV Scientist, LEO Pharma A/S, Industriparken 55, 2750 Ballerup, Denmark, Tel.: +45 44 94 58 88, Fax: +45 72 26 33 21, e-mail: bjarke.naver@leo-pharma.com

6.3.5 Study Statistician

Kim Mark Knudsen, PhD Stat, Senior Biostatistician, LEO Pharma A/S, Industriparken 55, 2750 Ballerup, Denmark, Tel.: +45 72 26 30 99, Fax: +45 72 26 33 21, e-mail: kim.mark-knudsen@leo-pharma.com

6.3.6 Study Data Manager

Ulla Wiberg, Clinical Data Manager, LEO Pharma A/S, Industriparken 55, 2750 Ballerup, Denmark, Tel.: +45 72 26 31 00, Fax: +45 72 26 33 21, e-mail: ulla.wiberg@leo-pharma.com

6.3.7 Pre-clinical Specialist

Kresten Skak, Senior Scientist, LEO Pharma A/S, Industriparken 55, 2750 Ballerup, Denmark, Tel.: +45 72 26 25 93, Fax: +45 72 26 33 21, e-mail: kresten.skak@leo-pharma.com

L E O
THIS DOCUMENT CONTAINS TRADE SECRETS, OR COMMERCIAL OR FINANCIAL INFORMATION, PRIVILEGED OR CONFIDENTIAL, DELIVERED IN CONFIDENCE AND RELIANCE THAT SUCH INFORMATION WILL NOT BE COPIED OR MADE AVAILABLE TO ANY THIRD PARTY WITHOUT THE WRITTEN CONSENT OF LEO PHARMA A/S - LEO PHARMACEUTICAL PRODUCTS LTD. A/S

6.4 PROTOCOL AUTHOR(S)

Jacqueline Morley, Clinical Research Team Leader, Clinical Operations, LEO Pharma Australia, Level 3, Tower 1, 25 Montpelier Rd, Bowen Hills, QLD 4006 Australia, Tel.: +61 7 2350 1244, Fax: +61 7 3250 1299, e-mail: jacqueline.morley@leo-pharma.com

Julia Delfs, Dipl. oec. troph., International Clinical Trial Manager, Clinical Operations, LEO Pharma GmbH, Frankfurter Straße 233, A3, D-63263 Neu-Isenburg, Germany, Tel.: +49 6102 201 113, Fax: +49 6102 201 100, e-mail: julia.delfs@leo-pharma.com

Kim Mark Knudsen, PhD Stat, Senior Biostatistician, LEO Pharma A/S, Industriparken 55, 2750 Ballerup, Denmark, Tel.: +45 72 26 30 99, Fax: +45 72 26 33 21, e-mail: kim.mark-knudsen@leo-pharma.com

Torsten Skov, Senior Medical Advisor, LEO Pharma A/S, Industriparken 55, 2750 Ballerup, Denmark, Tel.: +45 72 26 39 90, Fax: +45 72 26 33 21, Mobile: +45 20 73 62 94,
e-mail: torsten.skov@leo-pharma.com

Bjarke Naver, MSc PhD, Principal PV Scientist, LEO Pharma A/S, Industriparken 55, 2750 Ballerup, Denmark, Tel.: +45 44 94 58 88, Fax: +45 72 26 33 21, e-mail: bjarke.naver@leo-pharma.com

Kresten Skak, Senior Scientist, LEO Pharma A/S, Industriparken 55, 2750 Ballerup, Denmark, Tel.: +45 72 26 25 93, Fax: +45 72 26 33 21, e-mail: kresten.skak@leo-pharma.com

Prof. Dr. Michael P. Schön, Universitätsmedizin Göttingen, Georg-August-Universität, Abteilung Dermatologie, Venerologie und Allergologie, Von-Siebold-Straße 3, D-37075 Göttingen, Germany, e-mail: michael.schoen@med.uni-goettingen.de

LEO

THIS DOCUMENT CONTAINS TRADE SECRETS, OR COMMERCIAL OR FINANCIAL INFORMATION, PRIVILEGED OR CONFIDENTIAL, DELIVERED IN CONFIDENCE AND RELIANCE THAT SUCH INFORMATION WILL NOT BE COPIED OR MADE AVAILABLE TO ANY THIRD PARTY WITHOUT THE WRITTEN CONSENT OF LEO PHARMA A/S - LEO PHARMACEUTICAL PRODUCTS LTD. A/S

6.5 INVESTIGATORS AND TRIAL COMMITTEES

6.5.1 International Co-ordinating Investigator

The International Co-ordinating Investigator is responsible for approval of the (Consolidated) Clinical Study Protocol, Clinical Study Protocol Addendum(s), (Consolidated) CRF and the Clinical Study Report on behalf of all trial investigators, and as agreed to in an International Co-ordinating Investigator Agreement.

Prof. Dr. Michael P. Schön
Universitätsmedizin Göttingen
Georg-August-Universität
Abteilung Dermatologie, Venerologie und Allergologie
Von-Siebold-Straße 3
D-37075 Göttingen
Germany
michael.schoen@med.uni-goettingen.de

6.5.2 National Co-ordinating Investigators

The National Co-ordinating Investigators are responsible for national issues relating to the clinical trial as agreed to in a National Co-ordinating Investigator Agreement.
The contact details of participating National Co-ordinating Investigator(s) are provided outside the protocol in the "National Clinical Trial Applications".

Not applicable, single site only.

6.5.3 Investigators

Each participating investigator is responsible for all aspects of the clinical trial conduct at his/her trial site as agreed to in a clinical trial agreement.
The contact details of each participating investigator(s) are provided outside the protocol in the "National Clinical Trial Applications".

Not applicable, single site only.

6.5.4 Trial Committees

Not applicable.

L E O
THIS DOCUMENT CONTAINS TRADE SECRETS, OR COMMERCIAL OR FINANCIAL INFORMATION, PRIVILEGED OR CONFIDENTIAL, DELIVERED IN CONFIDENCE AND RELIANCE THAT SUCH INFORMATION WILL NOT BE COPIED OR MADE AVAILABLE TO ANY THIRD PARTY WITHOUT THE WRITTEN CONSENT OF LEO PHARMA A/S - LEO PHARMACEUTICAL PRODUCTS LTD. A/S

6.6 AGREEMENTS

Before the initiation of any clinical trial related activities by the investigators/clinical trial committee(s)/LEO Pharma A/S affiliate(s)/CRO(s) listed above, the relevant parties must have entered into a written agreement regulating those activities.

7 INSURANCE

LEO Pharma A/S has taken out relevant insurances covering the subjects in the present clinical trial in accordance with applicable laws and regulations.

8 INTRODUCTION AND RATIONALE

The study is a phase I, single-centre, open label, within-subject comparison trial to explore the biological effects of PEP005 (ingenol mebutate) Gel, 0.05%, applied once daily for 2 consecutive days in patients with Actinic Keratosis (AK) on the upper extremity. The investigational product under investigation is in phase 3 of the clinical development program. This study will be conducted in accordance with applicable national regulatory requirements.

8.1 ACTINIC KERATOSIS

Actinic Keratosis is a common skin condition visible as thickened, cornified, scaly lesions and characterised histological by atypical epithelial proliferation (14). Actinic keratoses usually develop on areas that are frequently exposed to the sun (e.g., face, lips, ears, scalp, neck, forearms, and back of the hands). Patients with AK often express embarrassment, worry, and irritation related to the change in appearance of their skin and unsightly nature of the lesions (15, 16). In addition to the emotional strain, AK lesions can be painful and easily traumatized causing bleeding (17, 18, 19). In Europe the prevalence rate is from 11-25% for people aged 40 or older (20, 21). There is increasing evidence that AK represents SCC in situ in its earliest stages (14, 22, 23). Histological evidence shows that contiguous AK is present in 97% of SCC lesions on sun-damaged skin (23). Actinic keratosis is linked epidemiologically to development of SCC (1), and both conditions share specific gene expression (24). If left untreated, AK may progress to SCC, with significant morbidity and death (22).

Current treatment options for AK lesions consist of cryotherapy, photodynamic therapy, and topical products. Photodynamic therapy and cryotherapy can be painful, and patients are often left with hypopigmented spotting where cryotherapy is applied (2, 3). Curettage (with or

THIS DOCUMENT CONTAINS TRADE SECRETS, OR COMMERCIAL OR FINANCIAL INFORMATION, PRIVILEGED OR CONFIDENTIAL, DELIVERED IN CONFIDENCE AND RELIANCE THAT SUCH INFORMATION WILL NOT BE COPIED OR MADE AVAILABLE TO ANY THIRD PARTY WITHOUT THE WRITTEN CONSENT OF LEO PHARMA A/S - LEO PHARMACEUTICAL PRODUCTS LTD. A/S

without electrosurgery) and excisional surgery are alternatives to cryosurgery (4). Topical products include 5-FU, diclofenac, and imiquimod, and are commonly used as field treatment for multiple lesions over larger skin areas. Fluorouracil is approved in the US under the brand names of Efudex®, Fluoroplex®, and Carac® (5, 6, 7) and in the EU under the brand name of Efudix®. (8) Diclofenac is approved in the US and EU under the brand name of Solaraze® (9, 10) Imiquimod in approved in the US under the brand names of Aldara® (11) and Zyclara® (12) and in the EU under the brand name of Aldara® (13). Photodynamic therapy, Carac® (7), Aldara® (11) and Zyclara® (12) are indicated for treatment of AK lesions on the face and scalp only.

8.2 INVESTIGATIONAL PRODUCT DESCRIPTION

Ingenol mebutate is the active compound in the sap from *Euphorbia peplus L.* (*E. peplus*). The sap from *E. peplus* has a long history of community use for the topical treatment of various skin conditions, including actinic keratosis (AK). Ingenol mebutate, in a gel formulation referred to as PEP005 (ingenol mebutate) Gel or PEP005 Gel, has been evaluated in clinical trials as field therapy for the topical treatment of AK and photo damaged skin and as lesion-specific treatment for seborrhoeic keratosis and non-melanoma skin cancer (NMSC).

The formulation of PEP005 Gel contains ingenol mebutate as the active ingredient and benzyl alcohol, hydroxethyl cellulose, isopropyl alcohol, citric acid, sodium citrate, and purified water as inactive ingredients. This gel formulation does not contain any inactive ingredients that would result in acute toxicity to the skin and is water soluble.

Safety and efficacy data have been obtained from studies designed to support the use of PEP005 Gel as a lesion-specific treatment for NMSC and as field therapy for AK. Over 2400 patients and healthy volunteers have participated in the PEP005 Gel clinical program.

For further information regarding the safety and efficacy profile of PEP005 Gel please refer to Investigators Brochure LEO PEP005 Field Therapy Edition no. 1.

8.3 TRIAL RATIONALE

The current knowledge on the mechanism of action of ingenol mebutate is based on in vitro studies in human cell lines and in vivo studies in mice models (25, 32). In vitro, high concentrations of ingenol mebutate (>100 µM) leads to direct cell death (primary necrosis), whereas

L E O
THIS DOCUMENT CONTAINS TRADE SECRETS, OR COMMERCIAL OR FINANCIAL INFORMATION, PRIVILEGED OR CONFIDENTIAL, DELIVERED IN CONFIDENCE AND RELIANCE THAT SUCH INFORMATION WILL NOT BE COPIED OR MADE AVAILABLE TO ANY THIRD PARTY WITHOUT THE WRITTEN CONSENT OF LEO PHARMA A/S - LEO PHARMACEUTICAL PRODUCTS LTD. A/S

at low concentrations (10 – 1000 nM) ingenol mebutate is a potent activator of protein kinase C (PKC) leading to cytokine and chemokine release and vascular endothelial activation. In vivo, an infiltration of leukocytes and in particular neutrophils into the application site has been observed after topical application in mice, and haemorrhage in the dermis is prominent. Interestingly, clinical observations in humans treated with ingenol mebutate suggest local necrosis and inflammatory reaction but no haemorrhage. These findings have not been investigated by histological studies and are only based on visual evaluation of the subjects.

8.3.1 Necrotic Cell Death

Both in vitro and in vivo studies have shown that ingenol mebutate can induce necrotic cell death (25). Ingenol mebutate (100–200 μM) rapidly induced necrotic cell death in several human and murine tumour cell lines in vitro, including melanoma and SCC. Cell death appeared to be due to necrosis, rather than apoptosis, based on electron microscopic and other experimental evidence. Mitochondrial swelling was the first visually observable change in cellular morphology (within hours); however, plasma membrane perturbation has been experimentally observed within 30 minutes of exposure. Ultimately, ingenol mebutate-treated cells undergo an irreversible loss of mitochondrial membrane potential, which leads to necrotic cell death. Although some changes suggestive of apoptotic cell death have been observed, several key characteristics of apoptosis (e.g. margination of chromatin, DNA laddering) were not evident.

8.3.2 Immune Modulation

In vitro, ingenol mebutate has been shown to stimulate the production of numerous pro-inflammatory cytokines (e.g., interleukin-8, interleukin-1β, tumour necrosis factor-α, interleukin-2 and interleukin-6) from peripheral blood mononuclear cells, neutrophils, fibroblasts, keratinocytes and melanoma cells (32). Importantly, topical ingenol mebutate treatment in mice at the skin site harbouring syngeneic tumours induced a similar set of cytokines (e.g., macrophage inflammatory protein 2, tumour necrosis factor-α, interleukin-1β) in local tissue (32). These factors may have been induced directly by ingenol mebutate by its ability to activate PKC in keratinocytes, tumour cells and vascular endothelial cells or indirectly as a consequence of tissue necrosis induced by ingenol mebutate (26, 27, 28). Thus, direct and indirect cellular activation by ingenol mebutate results in chemotaxis of neutrophils to the treated site. Most significantly, ingenol mebutate has been shown to induce E-selectin and intracellular adhesion molecule 1 expression in endothelial cells via PKC activation, ultimately leading to the adhesion and extravasation of neutrophils through the vessel walls and into the surrounding tissue (29).

LEO

THIS DOCUMENT CONTAINS TRADE SECRETS, OR COMMERCIAL OR FINANCIAL INFORMATION, PRIVILEGED OR CONFIDENTIAL, DELIVERED IN CONFIDENCE AND RELIANCE THAT SUCH INFORMATION WILL NOT BE COPIED OR MADE AVAILABLE TO ANY THIRD PARTY WITHOUT THE WRITTEN CONSENT OF LEO PHARMA A/S - LEO PHARMACEUTICAL PRODUCTS LTD. A/S

In addition to the role of ingenol mebutate in the activation of PKC and stimulation of cell adhesion and pro-inflammatory molecules, several key in vivo studies have also clearly shown the importance of the immune system in the anti-tumour activity of ingenol mebutate (32). Topical ingenol mebutate application to murine tumours resulted in long-term tumour regression and clearance. However, in neutrophil-depleted mice, tumour relapse rates were significantly increased following topical treatment (32). As observed in wild type mice, tumour burden was reduced as a result of ingenol mebutate-induced cell death (necrosis); however, the tumours relapsed after 20-40 days, indicating that a small percentage of tumour cells had escaped the direct necrotic effect of ingenol mebutate.

The primary objective of the present study is to explore the biological effects following treatment with PEP005 Gel, 0.05% administered for two consecutive days. The focus will be on mechanisms and biological processes which can be assessed on biopsies from the application site, mainly with standard histological methods and immunohistochemistry. The main hypotheses of the study are that

- Necrosis and/or apoptosis will be observed on Day 2 and Day 3
- Infiltration of leukocytes will be observed on Day 2 and Day 3 with infiltration of lymphocytes and other mononuclear cells, polymorphonuclear cells and mast cells
- Vascular endothelial cells will be activated and up-regulate expression of adhesion molecules
- Hemorrhage in the dermis will not be induced (in contrast to mice)

The secondary objective is to compare the skin response in Normal skin and AK lesions after treatment with PEP005 gel 0.05% administered for two consecutive days. Based on case reports it is hypothesised that the skin reaction will be stronger in AK lesions compared to Normal skin, and this hypothesis will be explored here.

The information obtained in this study about the biological effects of ingenol mebutate will provide an important link to the biological effects observed in animal models. This understanding will be valuable for the further development of ingenol mebutate for the treatment of skin cancer as well as other indications, for optimizing new formulations of ingenol mebutate and for the selection of second generation drugs with even better efficacy and tolerability.

Design

THIS DOCUMENT CONTAINS TRADE SECRETS, OR COMMERCIAL OR FINANCIAL INFORMATION, PRIVILEGED OR CONFIDENTIAL, DELIVERED IN CONFIDENCE AND RELIANCE THAT SUCH INFORMATION WILL NOT BE COPIED OR MADE AVAILABLE TO ANY THIRD PARTY WITHOUT THE WRITTEN CONSENT OF LEO PHARMA A/S - LEO PHARMACEUTICAL PRODUCTS LTD. A/S

To elucidate the above mentioned hypotheses of the study a design has been devised in which patients with AK will be treated with PEP005 Gel with the same concentration and schedule as has been proven effective in the Phase 3 program. Biopsies taken on the treatment days will allow the identification of biological processes associated with the efficacy of the product. In order for the necessary biopsies to be associated with as little discomfort as possible, the upper extremity has been preferred over the alternative of studying lesions on the face and scalp.

9 TRIAL OBJECTIVES

9.1 PRIMARY OBJECTIVE

To explore the biological effects following treatment with PEP005 Gel, 0.05% administered for two consecutive days.

9.1.1 Primary Outcome Measures

1. Degree of skin infiltration of leukocytes in haematoxylin & eosin-stained sections assessed on a scale from 0 to 3 in the AK biopsies from Day 1, Day 2, and Day 3
2. Degree of necrosis of the epidermis and dermis in haematoxylin & eosin-stained sections assessed as binary outcome (present/absent) in the AK biopsies from Day 1, Day 2, and Day 3

9.2 SECONDARY OBJECTIVES

To compare the skin response in Normal skin and AK lesions after treatment with PEP005 gel 0.05% administered for two consecutive days.

9.2.1 Secondary Outcome Measures

1. Degree of skin infiltration of leukocytes in haematoxylin & eosin-stained sections assessed on a scale from 0 to 3 in the normal skin biopsies from day 3 compared to baseline and to AK lesion biopsies
2. Degree of necrosis of the epidermis and dermis in haematoxylin & eosin-stained sections assessed as binary outcome (present/absent) in the normal skin biopsies from day 3 compared to baseline and to AK lesion biopsies
3. Degree of haemorrhage assessed by histology (binary outcome present/absent) in the Normal skin and AK lesion biopsies at all time points

LEO

THIS DOCUMENT CONTAINS TRADE SECRETS, OR COMMERCIAL OR FINANCIAL INFORMATION, PRIVILEGED OR CONFIDENTIAL, DELIVERED IN CONFIDENCE AND RELIANCE THAT SUCH INFORMATION WILL NOT BE COPIED OR MADE AVAILABLE TO ANY THIRD PARTY WITHOUT THE WRITTEN CONSENT OF LEO PHARMA A/S - LEO PHARMACEUTICAL PRODUCTS LTD. A/S

4. Characterisation of immune cells such as T and B lymphocytes, polymorphonuclear cells, mast cells and antigen presenting cells in the Normal skin and AK lesion biopsies
5. Degree of apoptosis in the Normal skin and AK lesion biopsies
6. Degree of vascular endothelium activation in the Normal skin and AK lesion biopsies
7. Changes in mRNA and miRNA expression in the Normal skin and AK lesion biopsies
8. Expression of drug transporters in the Normal skin and AK lesion biopsies (P-gP)

10 INVESTIGATIONAL PLAN

10.1 TRIAL DESIGN

10.1.1 Overall Design

A Phase I, single-centre, open label, within-subject comparison trial to explore the biological effects of PEP005 (ingenol mebutate) Gel, 0.05%, applied once daily for 2 consecutive days in patients with AK on the upper extremity.

27 subjects will be enrolled into this study. The subject will attend 6 visits in total:

- Screening visit (Day -7 to Day 1)
- Visit 1 (Day 1, biopsy, first treatment day)
- Visit 2 (Day 2, biopsy, second treatment day)
- Visit 3 (Day 3, biopsy)
- Visit 4 (Day 8 +/- 3 days, Follow up)
- Visit 5 (Day 29 +/- 3 days, End of trial)

Note: Screening Visit / Day 1 can be on same day. The Investigational Product (IP) will be (sub)investigator applied at the site on Visits 1 and 2.

All eligible patients will receive PEP005 Gel, 0.05%, on two consecutive Days (Day 1 and Day 2) to both the AK Treatment Area and Normal Skin Treatment Area as defined below:

1. “AK Treatment Area”: A contiguous area of 25 cm^2 of skin on the upper extremity (incl. dorsum manus) that contains 2 to 5 AK lesions. Additionally there must be at least one AK lesion located within 1 to 5 cm outside of the selected AK Treatment Area. The AK Treatment Area and the AK lesion outside the area will be identified on the transparency sheet at the screening visit.

THIS DOCUMENT CONTAINS TRADE SECRETS, OR COMMERCIAL OR FINANCIAL INFORMATION, PRIVILEGED OR CONFIDENTIAL, DELIVERED IN CONFIDENCE AND RELIANCE THAT SUCH INFORMATION WILL NOT BE COPIED OR MADE AVAILABLE TO ANY THIRD PARTY WITHOUT THE WRITTEN CONSENT OF LEO PHARMA A/S - LEO PHARMACEUTICAL PRODUCTS LTD. A/S

2. "Normal Skin Treatment Area": A contiguous area of 25 cm^2 of normal skin that has no or only minimal sun-damage from the inner upper arm. The Normal Skin Treatment Area will be identified on a transparency sheet at the screening visit.

As mentioned above, the AK Treatment Area will be identified in such a way that there is at least one AK lesion outside the area at a distance of 1 to 5 cm from the margin of the AK Treatment Area (this AK lesion outside the AK Treatment Area will be biopsied at Day 1).

Of the lesions within the AK Treatment Area, 2 will be selected for biopsy during and after treatment. One of them will be biopsied at Day 2, the other one at Day 3. Biopsied sites will be excluded (following the biopsy being performed) from treatment (i.e. the site of the Day 2 biopsy will be treated on Day 1 but not on Day 2). The distance between the area treated with PEP005 Gel on Day 2 and the Day 2 biopsy site has to be approx. 1 cm.

For the Normal Skin Treatment Area the baseline biopsy will be taken within 1 to 5 cm away from the selected treatment area. After two days of PEP005 gel, 0.05%, treatment (Visit 3), a second biopsy will be taken within the selected treatment area (the location of this biopsy will be according to investigators discretion but must be within the treatment area).

10.1.2 Individual Periods

10.1.2.1 Screening Period (Day -7 to Day 1)

Subjects will undergo a study specific screening visit (Day -7 to -1) prior to enrolment (this visit can also be conducted on the same day as Visit 1). Subjects will sign the study-specific informed consent form in the presence of the (sub)investigator prior to any screening/baseline procedures taking place. The following information and screening assessments will be recorded: demographics (date of birth, sex, race, ethnic origin), Fitzpatrick skin type, concomitant medication, concomitant diagnoses, and confirmation of inclusion/exclusion criteria. In addition, relevant medical history as per investigator evaluation will be recorded. Vital signs, physical examination, and for female subjects of child bearing potential a urine pregnancy test, will be performed at the screening visit and repeated at Visit 1 prior to enrolment. The two selected treatment areas as defined below will

LEO
THIS DOCUMENT CONTAINS TRADE SECRETS, OR COMMERCIAL OR FINANCIAL INFORMATION, PRIVILEGED OR CONFIDENTIAL, DELIVERED IN CONFIDENCE AND RELIANCE THAT SUCH INFORMATION WILL NOT BE COPIED OR MADE AVAILABLE TO ANY THIRD PARTY WITHOUT THE WRITTEN CONSENT OF LEO PHARMA A/S - LEO PHARMACEUTICAL PRODUCTS LTD. A/S

be identified and photographs taken. Local Skin Response (LSR) within the two selected treatment areas will be recorded also.

1. "AK Treatment Area": A contiguous area of 25 cm^2 of skin on the upper extremity (incl. dorsum manus) that contains 2 to 5 AK lesions. Additionally there must be at least one AK lesion located within 1 to 5 cm outside of the selected AK Treatment Area. The AK Treatment Area and the AK lesion outside the area will be identified on the transparency sheet at the screening visit.

2. "Normal Skin Treatment Area": A contiguous area of 25 cm^2 of normal skin that has no or only minimal sun-damage from the inner upper arm. The Normal Skin Treatment Area will be identified on a transparency sheet at the screening visit.

Identification and documentation of the selected treatment areas is outlined in Appendix III. The location of the selected treatment area as well as biopsy locations will be verified by referencing the transparency on which it was mapped and the Screening photographs.

10.1.2.2 Treatment Period (Day 1, Day 2)

Day 1 (Visit 1)

On arrival at the site at Visit 1 (Day 1, baseline [this process may occur on the same day as the Screening if appropriate]), the subject will undergo the re-check assessments to confirm their eligibility to participate in the trial (inclusion/exclusion criteria, concomitant medication, medical history). Vital signs, physical examination, and for female subjects of child bearing potential a urine pregnancy test, will be performed. A CRF book number will be allocated to all patients for whom informed consent was obtained. Subjects who are not enrolled in the trial will have an End of Trial Form completed.

At Visit 1, the selected treatment areas identified at the screening visit will be marked as outlined in Appendix III.

Prior to study medication application on Visit 1, baseline biopsies (4 mm punch) will be taken from one AK lesion and from a normal skin area (1 to 5 cm outside the selected treatment areas). These biopsy sites are to be marked on the transparencies.

LEO

THIS DOCUMENT CONTAINS TRADE SECRETS, OR COMMERCIAL OR FINANCIAL INFORMATION, PRIVILEGED OR CONFIDENTIAL, DELIVERED IN CONFIDENCE AND RELIANCE THAT SUCH INFORMATION WILL NOT BE COPIED OR MADE AVAILABLE TO ANY THIRD PARTY WITHOUT THE WRITTEN CONSENT OF LEO PHARMA A/S - LEO PHARMACEUTICAL PRODUCTS LTD. A/S

The selected treatment areas will be assessed by a (sub)investigator before study medication application for skin adverse events (AEs) and LSRs. The assessment of LSRs and AEs should be performed by the same (sub)investigator for each subject throughout the trial period, where possible. Photographs of both treatment areas will also be taken prior to study medication application. For study medication application procedures please refer to section 10.6.3.

Day 2 (Visit 2)

Vital signs will be performed as well as concomitant medication recorded. The selected treatment areas will be assessed by a (sub)investigator before study medication application and biopsy for skin adverse events (AEs) and LSRs. Photographs of both treatment areas will also be taken prior to study medication application.

A single biopsy will be taken from one AK lesion within the selected AK Treatment Area prior to study medication application at Visit 2. This biopsy is to be recorded on the AK Treatment Area transparency. For more information regarding assessments refer to section 10.7, Trial Procedures.

10.1.2.3 Follow up Period: Day 3, Day 8, Day 29

After completion of the Treatment Period, each subject will return to the site 24 hours after the last study medication Visit 3 (Day 3), Visit 4 (Day 8 +/- 3 days) and again on Visit 5 (Day 29 +/- 3 days).

Day 3 (Visit 3)

Vital signs will be performed as well as concomitant medication recorded. The selected treatment areas will be assessed by a (sub)investigator for skin adverse events (AEs) and LSRs. Photographs of both treatment areas will also be taken.

Biopsies from one AK lesion and from Normal skin within the selected treatment areas will be performed. These biopsies are to be recorded on the transparencies accordingly.

Day 8 (Visit 4)

Vital signs will be performed as well as concomitant medication recorded. The selected treatment areas will be assessed by a (sub)investigator for skin adverse events (AEs) and LSRs. Photographs of both treatment areas will also be taken. If the subject is a woman of child-bearing potential, a urine pregnancy test will also be done at Visit 4.

Day 29 (Visit 5)

Vital signs will be performed as well as concomitant medication recorded. The selected treatment areas will be assessed by a (sub)investigator for skin adverse events

L E O
THIS DOCUMENT CONTAINS TRADE SECRETS, OR COMMERCIAL OR FINANCIAL INFORMATION, PRIVILEGED OR CONFIDENTIAL, DELIVERED IN CONFIDENCE AND RELIANCE THAT SUCH INFORMATION WILL NOT BE COPIED OR MADE AVAILABLE TO ANY THIRD PARTY WITHOUT THE WRITTEN CONSENT OF LEO PHARMA A/S - LEO PHARMACEUTICAL PRODUCTS LTD. A/S

(AEs) and LSRs. Photographs of both treatment areas will also be taken. An End of Trial Form will also be completed.

Once a subject has completed the clinical trial, the investigator should follow-up for outcome on all non-Serious Adverse Events classified as possibly/probably related to the investigational product or not assessable until resolution or the follow-up assessment, whichever comes first.

A diagram of the study design is provided in section 10.1.3.

10.1.3 Overall Chart

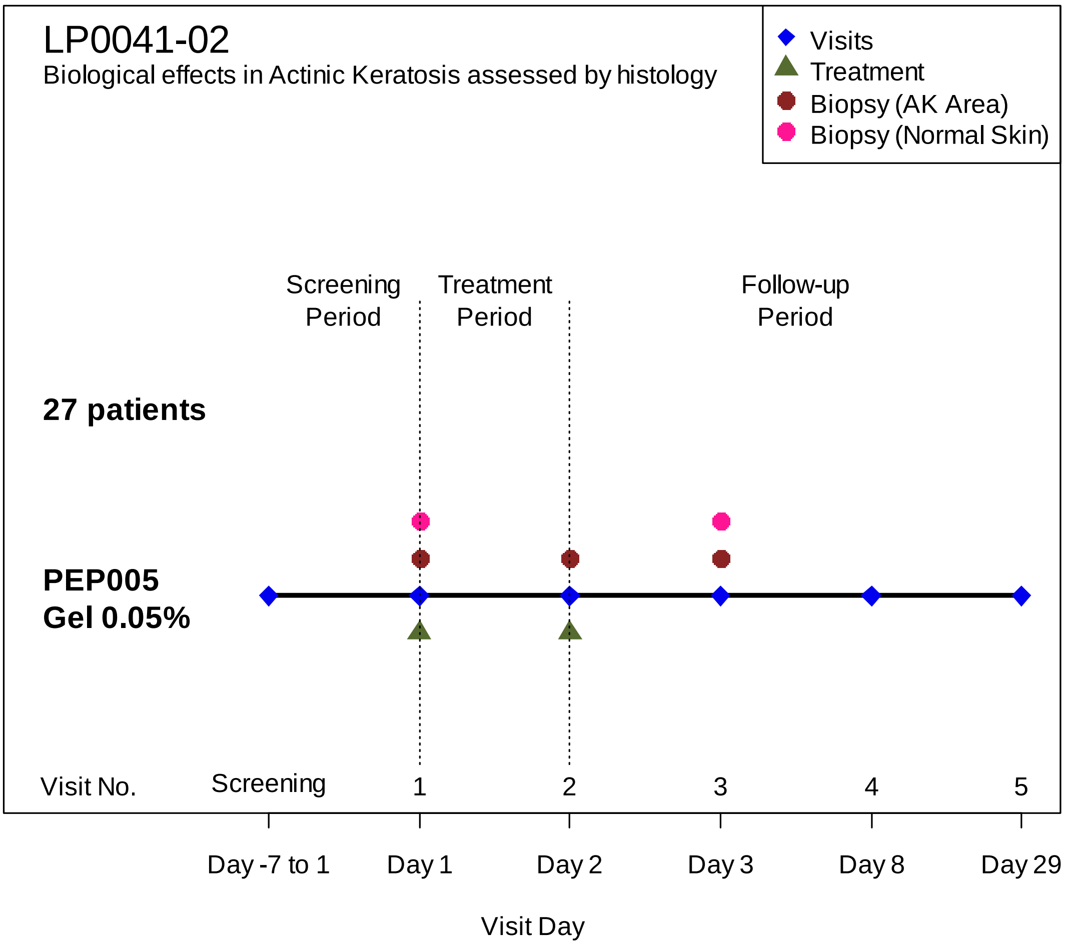

THIS DOCUMENT CONTAINS TRADE SECRETS, OR COMMERCIAL OR FINANCIAL INFORMATION, PRIVILEGED OR CONFIDENTIAL, DELIVERED IN CONFIDENCE AND RELIANCE THAT SUCH INFORMATION WILL NOT BE COPIED OR MADE AVAILABLE TO ANY THIRD PARTY WITHOUT THE WRITTEN CONSENT OF LEO PHARMA A/S - LEO PHARMACEUTICAL PRODUCTS LTD. A/S

10.2 TIME SCHEDULE

Planned date of enrolment of first subject: July 2011
Planned date of enrolment of last subject: January 2012
Planned date of completion of last subject: February 2012

10.3 NUMBER OF SUBJECTS/SAMPLE SIZE

In order to obtain a precision corresponding to the half-width of the 95% confidence interval of 0.20 when the true response rate is 0.50 with more than 90% power, 27 subjects are required.

10.4 CRITERIA FOR SUBJECT SELECTION (IN- AND EXCLUSION)

Following receipt of verbal and written information about the clinical trial, the subject must provide **signed and dated informed consent** before any trial related activity is carried out, including any screening activities.

Any implementation of national requirements/law for the subject's participation in the clinical trial will be ensured and will be described in submission documentation to authorities/ethics committees, as applicable.

10.4.1 Inclusion Criteria

1. Male or female patients at least 18 years of age
2. Patients with 2 to 5 clinically typical, visible and discrete AK lesions within a contiguous 25 cm^2 area (AK Treatment Area) on the upper extremity; with one additional AK lesion located 1 to 5 cm from the AK Treatment Area
3. Patients with a 25 cm^2 area of normal skin (Normal Skin Treatment Area) on the upper inner arm suitable for treatment and biopsy
4. Female subjects must be of either:
    - Non-childbearing potential, post-menopausal, or have a confirmed clinical history of sterility (e.g. the subject is without a uterus) or,
    - Childbearing potential, provided there is a confirmed negative urine pregnancy test prior to exposure, to rule out pregnancy.
5. Female subjects of childbearing potential[2] must be willing to consent to using high effective methods of **contraception (Pearl index < 1%)** at study entry and for the duration of the trial participation. Adequate methods of contraception are defined as

[2] Female subjects are considered of childbearing potential unless they have been hysterectomised or have undergone tubal ligation or have been post-menopausal for at least one year prior to screening visit

L E O
THIS DOCUMENT CONTAINS TRADE SECRETS, OR COMMERCIAL OR FINANCIAL INFORMATION, PRIVILEGED OR CONFIDENTIAL, DELIVERED IN CONFIDENCE AND RELIANCE THAT SUCH INFORMATION WILL NOT BE COPIED OR MADE AVAILABLE TO ANY THIRD PARTY WITHOUT THE WRITTEN CONSENT OF LEO PHARMA A/S - LEO PHARMACEUTICAL PRODUCTS LTD. A/S

- abstinence (when this is in line with the preferred and usual lifestyle of the subject)
- vasectomised partner (partner should be the sole partner for the subject)
- an intrauterine device
- double barrier method defined as two distinct methods (either two actual barrier methods or one actual barrier method and one hormonal method)
- hormonal contraceptive (oral hormonal birth control, estrogenic vaginal ring, percutaneous contraceptive patches, implants and injectables) for at least one menstrual cycle prior to enrolment (Visit 1)

6. Ability to follow study instructions and likely to complete all study requirements
7. Obtained written informed consent prior to any study-related procedures
8. Agreement from the patient to allow photographs of the selected treatment area to be taken and used as part of the study data package

10.4.2 Exclusion Criteria

1. Location of the selected treatment areas:
   - Within 5 cm of an incompletely healed wound or infected area of the skin (excl. study related biopsies)
   - Within 10 cm of a suspected basal cell carcinoma (BCC) or squamous cell carcinoma (SCC)
2. History or evidence of skin conditions other than the study indication that would interfere with evaluation of the study medication (e.g. eczema, unstable psoriasis, xeroderma pigmentosum).
3. Prior treatment with PEP005 Gel
4. Treatment area lesions that have an atypical clinical appearance (e.g. hypertrophic, hyperkeratotic, recalcitrant disease [had cryosurgery on two previous occasions] and/or cutaneous horns).
5. Known sensitivity or allergy to any of the ingredients of PEP005 Gel (e.g. citric acid)
6. Clinical diagnosis/history or evidence of any medical condition that would expose a subject to an undue risk of a significant AE or interfere with assessments of safety during the course of the study, as determined by Investigator clinical judgment.
7. Anticipated need for in-patient hospitalisation or in-patient surgery during the study period. Note that cosmetic/therapeutic procedures are not excluded if they fall outside of the criteria detailed in the Prohibited Therapies or Medications (listed below).
8. Anticipated excessive or prolonged exposure to ultraviolet light (e.g. sunlight) or use of tanning beds for the duration of the study.
9. Current participation in any other interventional clinical trial.

L E O

THIS DOCUMENT CONTAINS TRADE SECRETS, OR COMMERCIAL OR FINANCIAL INFORMATION, PRIVILEGED OR CONFIDENTIAL, DELIVERED IN CONFIDENCE AND RELIANCE THAT SUCH INFORMATION WILL NOT BE COPIED OR MADE AVAILABLE TO ANY THIRD PARTY WITHOUT THE WRITTEN CONSENT OF LEO PHARMA A/S - LEO PHARMACEUTICAL PRODUCTS LTD. A/S

10. Subjects who have received treatment with any **non-marketed drug product** (i.e. an agent which has not yet been made available for clinical use following registration) within the last two months.
11. Subject known or, in the opinion of the investigator, is **unlikely to comply** with the Clinical Study Protocol (e.g. alcoholism, drug dependency or psychotic state).
12. Females who are **pregnant**, of child-bearing potential and wishing to become pregnant during the trial, or are **breast feeding**.
13. Females of child-bearing potential with **positive pregnancy test** at [screening or visit 1].
14. Previous enrolment in this clinical trial.

Prohibited Therapies and/or Medications: **within 2 weeks prior to the Screening visit**

15. Cosmetic or therapeutic procedures (e.g. use of liquid nitrogen, surgical excision, curettage, dermabrasion, medium or greater depth chemical peel, laser resurfacing): within 2 cm of the selected treatment areas and within 2 cm of the selected AK lesion outside the treatment area
16. Use of acid-containing therapeutic products (e.g. salicylic acid or fruit acids, such as alpha and beta hydroxy acids and glycolic acids), topical retinoids or light chemical peels: within 2 cm of the selected treatment areas and within 2 cm of the selected AK lesion outside the treatment area
17. Use of topical salves (non-medicated/non-irritant lotion/cream are acceptable) or topical steroids: within 2 cm of the selected treatment areas and within 2 cm of the selected AK lesion outside the treatment area; artificial tanners: within 5 cm of the selected treatment areas and within 5 cm of the selected AK lesion outside the treatment area

Prohibited Therapies and/or Medications: **within 4 weeks prior to** the Screening visit

18. Treatment with immunomodulators (e.g. azathioprine), cytotoxic drugs (e.g. cyclophosphamide, vinblastine, chlorambucil, methotrexate, podophyllin, camptothecin) or interferon/interferon inducers.
19. Treatment with systemic medications that suppress the immune system (e.g. cyclosporine, prednisone, methotrexate, alefacept, infliximab).
20. Treatment/therapy with UVB

Prohibited Therapies and/or Medications: **within 8 weeks prior to the Screening visit**

21. Treatment with 5-FU, imiquimod, diclofenac, or photodynamic therapy: within 2 cm of the selected treatment areas and within 2 cm of the selected AK lesion outside the treatment area

L E O
THIS DOCUMENT CONTAINS TRADE SECRETS, OR COMMERCIAL OR FINANCIAL INFORMATION, PRIVILEGED OR CONFIDENTIAL, DELIVERED IN CONFIDENCE AND RELIANCE THAT SUCH INFORMATION WILL NOT BE COPIED OR MADE AVAILABLE TO ANY THIRD PARTY WITHOUT THE WRITTEN CONSENT OF LEO PHARMA A/S - LEO PHARMACEUTICAL PRODUCTS LTD. A/S

Prohibited Therapies and/or Medications: **within 6 months prior to the Screening visit**

22. Use of systemic retinoids (e.g. isotretinoin, acitretin, bexarotene)

10.4.3 Subject Screening Log

A subject screening log will be maintained by the site for this study. All subjects who sign an informed consent will be included on the screening log. Date of screening, date of birth, sex and reason for non-inclusion, as applicable, should be stated on the log.

10.4.4 Subject Registration

At the screening visit each subject will be assigned the next (ascending) CRF book number available at the trial site. The CRF book number is a unique subject identifier used throughout the trial, in lieu of the subject's name.

10.5 WITHDRAWAL CRITERIA

Subjects **may** be withdrawn from the study for any of the following reasons:

1. *Voluntary withdrawal*: subjects will be free to withdraw from the clinical trial at any time and for any reason.
2. *Unacceptable adverse events*: any adverse event that the investigator or the subject considers unacceptable.
3. *Exclusion criteria*: any exclusion criteria which emerge/become apparent during the subject's participation in the clinical trial.
4. *Death*
5. *Lost to Follow Up*
6. *Other reasons*: other reasons than stated above which requires the subject to (be) withdraw(n) should be specified.

If the subject has been exposed to PEP005 Gel, the subject should remain in the trial and attend the follow up visits at Day 8 and 29, if possible.

Subjects **must** be withdrawn from the treatment for any of the following reasons:

1. if they *become pregnant* (additional follow up is required until delivery)
2. *Unacceptable adverse events*: any adverse event that the investigator or the subject considers unacceptable.

L E O
THIS DOCUMENT CONTAINS TRADE SECRETS, OR COMMERCIAL OR FINANCIAL INFORMATION, PRIVILEGED OR CONFIDENTIAL, DELIVERED IN CONFIDENCE AND RELIANCE THAT SUCH INFORMATION WILL NOT BE COPIED OR MADE AVAILABLE TO ANY THIRD PARTY WITHOUT THE WRITTEN CONSENT OF LEO PHARMA A/S - LEO PHARMACEUTICAL PRODUCTS LTD. A/S

3. *Voluntary withdrawal*: subjects will be free to withdraw from the treatment at any time and for any reason.
4. *Other reasons*: other reasons than stated above which requires the subject to (be) withdraw(n) should be specified.

Subjects who are discovered, after enrolment, not to have fulfilled all in/exclusion criteria at time of enrolment, should be withdrawn from treatment unless the investigator, based on clinical and ethical evaluation, finds withdrawal inappropriate. Such deviation(s) from the (Consolidated) Clinical Study Protocol must be reported to LEO Pharma A/S (and IEC/IRB, as appropriate) and recorded in the Clinical Study Report.

Reason(s) for withdrawal will be recorded in the CRF.

10.6 INVESTIGATIONAL PRODUCTS

Finished product (brand) name (if available)/name investigational product	PEP005 Gel, 0.05% w/w
Formulation	Gel
Active ingredient name/concentration	Ingenol mebutate
Excipients	Isopropyl alcohol USP<br>Hydroxyethyl cellulose NF<br>Benzyl alcohol NF<br>Citric acid monohydrate USP<br>Sodium citrate dihydrate USP<br>Purified water USP
Pack size(s)	0.47g
Manufacturer's name of bulk medication (IP)	DPT Laboratories, Ltd.
Certifier's name of bulk medication (IP)	LEO Pharma A/S
Supplier's name	Almac Clinical Services, Inc.
Manufacturer's name of subject treatment packages	Almac Clinical Services, Inc.
Certifier's name of subject treatment packages	LEO Pharma A/S

L E O
THIS DOCUMENT CONTAINS TRADE SECRETS, OR COMMERCIAL OR FINANCIAL INFORMATION, PRIVILEGED OR CONFIDENTIAL, DELIVERED IN CONFIDENCE AND RELIANCE THAT SUCH INFORMATION WILL NOT BE COPIED OR MADE AVAILABLE TO ANY THIRD PARTY WITHOUT THE WRITTEN CONSENT OF LEO PHARMA A/S - LEO PHARMACEUTICAL PRODUCTS LTD. A/S

10.6.1 Packaging of Investigational Products

Investigational product will be supplied in single use, unit dose tubes.

PEP005 Gel 0.05% kit: 4 unit-dose tubes. Two tubes will be labelled with red labels for application to the selected AK Treatment Area (1 tube for Day 1 and 1 tube for Day 2) and two labels will be labelled with white labels for application to the selected Normal Treatment Area (1 tube for Day 1 and 1 tube for Day 2).
The medication kit and unit doses will be identified by a label in compliance with national laws and regulations. Primary and secondary packaging materials will be individually labelled.

10.6.2 Storage of Investigational Products

PEP005 Gel will be stored in a refrigerator 2ºC – 8ºC and should be stored in a safe and secure place inaccessible for children. Refrigerator temperature at the site must be recorded hourly and monitored daily.

10.6.3 Administration of Investigational Products

Route of administration	Topical
Dosing range	One unit-dose tube
Dosing frequency	Once daily exposure for 2 consecutive days. Two treatment areas.
Daily maximum	Two unit-dose tube
Time of day for dosing	For each subject, the exposures should be performed at the same time (+/- 2 hours) of the day.
Relation of time of dosing to dietary intake	Not applicable

Study medication must be applied by a (sub)investigator with dermatologic expertise at the study site on Visits 1 and 2. Dosing of study medication on Visit 2 will be based on the (sub)investigator's medical judgment of subject tolerance to the treatment (e.g., incidence of LSRs and AEs). If a subject does not receive the second dose of study medication, he/she should remain in the trial and attend the observation visits at Day 3, 8 and 29, if possible.

Study Medication Dispensing Procedures

LEO
THIS DOCUMENT CONTAINS TRADE SECRETS, OR COMMERCIAL OR FINANCIAL INFORMATION, PRIVILEGED OR CONFIDENTIAL, DELIVERED IN CONFIDENCE AND RELIANCE THAT SUCH INFORMATION WILL NOT BE COPIED OR MADE AVAILABLE TO ANY THIRD PARTY WITHOUT THE WRITTEN CONSENT OF LEO PHARMA A/S - LEO PHARMACEUTICAL PRODUCTS LTD. A/S

The following guidelines should be followed when preparing for application of study medication at the site:

1. Ensure that all evaluations have been completed prior to the application of the study medication.
2. Ensure the selected treatment areas have been defined by the (sub)investigator and marked on the transparencies and the skin before application of the study medication. Note: PEP005 Gel should not be applied in intertriginous areas.
3. Ensure the (sub)investigator uses a protective glove when applying study medication. Contact with skin other than the selected treatment area or inhalation of the study medication must be avoided.
4. Ensure that the subject is instructed with post-treatment guidelines and provided with a Subject Safety and Study Gel Instructions Sheet, as detailed in Appendix IV.

Study Medication Treatment Procedures

Study medication must be applied to the selected treatment areas by the (sub)investigator at the site according to the following procedure:

1. Remove the study medication kit from the refrigerator.
2. Remove the two unit dose tubes labelled “Visit 1” or “Visit 2”, as appropriate. Squeeze the entire contents of one unit-dose tube onto a glove protected finger (the red labelled tube is to be applied to the AK Treatment Area and the white labelled tube is to be applied the Normal Skin Treatment Area).
3. Spread the study medication evenly over the marked selected treatment areas.
4. Recap the tubes and place them back into the study medication kit. The study medication kit should be placed immediately back into the refrigerator.
5. Allow the study medication to dry for at least 15 minutes following application.

Note: The AK Treatment Area will be modified slightly at the Day 2 visit to ensure that the biopsied area is not re-treated. For more information regarding this please refer to Appendix III.

10.6.4 Precautions/Overdosage

Despite the IP being physician applied, the site must ensure that the subject is instructed with post treatment guidelines following dosing on Day 1. These guidelines will be defined in a Subject Safety and Study Gel Instructions Sheet which must be distributed to each subject along with a Study Subject Card to identify that the subject is participating in a clinical trial.

L E O

THIS DOCUMENT CONTAINS TRADE SECRETS, OR COMMERCIAL OR FINANCIAL INFORMATION, PRIVILEGED OR CONFIDENTIAL, DELIVERED IN CONFIDENCE AND RELIANCE THAT SUCH INFORMATION WILL NOT BE COPIED OR MADE AVAILABLE TO ANY THIRD PARTY WITHOUT THE WRITTEN CONSENT OF LEO PHARMA A/S - LEO PHARMACEUTICAL PRODUCTS LTD. A/S

10.6.4.1 Skin Exposure

Due to the possible irritant properties of PEP005 Gel, contact with areas of skin outside the selected treatment areas should be avoided. In cases of inadvertent skin contact with investigational product, including non-participants, the area must be washed immediately with copious volumes of soapy water (alkaline soap will assist in neutralising the ingenol mebutate).

10.6.4.2 Ocular Exposure

The risk for accidental ocular exposure with PEP005 Gel should be minimized. If accidental exposure occurs, the eye should be irrigated immediately and extensively. Any individual (subject or otherwise) must be referred immediately to an ophthalmologist for assessment following ocular exposure. All treatments are to be administered at the discretion of an ophthalmologist and/or the investigator. Topical cycloplegics and antibiotics are recommended; eye pads or anti-inflammatory eye drops may be considered. The subject should be followed closely in the first few days after the exposure to check for secondary infection and to assess visual acuity. Subjects should be warned that vision might worsen before improvement occurs.

Ocular exposure to PEP005 Gel is likely to cause irritation and precautions should be taken to avoid such accidental exposure. Any suspected exposure should be documented and brought to the attention of the Monitor. If ocular exposure does occur, the management strategies stated above to treat exposure are recommended.

10.6.4.3 Other Exposure

If PEP005 Gel is inhaled, assist the exposed person to fresh air. If it is swallowed, assist the exposed person with irrigating the mouth out with copious amounts of water – if the person is conscious. In both instances, immediate medical attention should be sought.

10.6.4.4 Hypersensitivity

Use of PEP005 Gel is contraindicated in subjects with a known hypersensitivity to any ingredient in the formulation (e.g., citric acid). No severe hypersensitivity reactions have been observed to date.

10.6.5 Treatment Assignment

Subjects who have been found to comply with all the protocol's inclusion and exclusion criteria will be assigned a study medication kit by site personnel from the block of drug

L E O
THIS DOCUMENT CONTAINS TRADE SECRETS, OR COMMERCIAL OR FINANCIAL INFORMATION, PRIVILEGED OR CONFIDENTIAL, DELIVERED IN CONFIDENCE AND RELIANCE THAT SUCH INFORMATION WILL NOT BE COPIED OR MADE AVAILABLE TO ANY THIRD PARTY WITHOUT THE WRITTEN CONSENT OF LEO PHARMA A/S - LEO PHARMACEUTICAL PRODUCTS LTD. A/S

provided to the site. A subject should be assigned the next (ascending) study medication kit number available at the trial site.

10.6.5.1 Randomisation Code List

Not applicable.

10.6.5.2 Subject Identification List

The investigator will maintain a list of all subjects included in the trial at the trial site including each subject's identity, date of enrolment and corresponding subject identification number, so that any subject may be identified if required for any reason. The list is kept by the investigator and will not be copied to LEO Pharma A/S.

10.6.6 Blinding of the Trial

Not applicable.

10.6.7 Breaking the Randomisation Code

Not applicable.

10.6.8 Drug Accountability and Compliance Checks

The Principle investigator is fully responsible for the investigational products at the trial site. Dispensing of investigational products may be delegated to, e.g. a hospital pharmacy as locally applicable.

The person responsible for dispensing the investigational products will be responsible for maintaining adequate control of the investigational products and for documenting all transactions with them. Investigational products must be stored in a safe and secure place, and proper dispensing arrangements must be made.

10.6.8.1 Sponsor-Investigator Drug Accountability

All investigational products supplied by the Contract Manufacturing Organisation (CMO) on behalf of LEO Pharma A/S will be returned to the CMO and be fully accounted for by the monitor with the help of the person responsible for dispensing the investigational products. Accountability will be documented by use of drug accountability forms.

Investigational products may be returned from the trial site either to the CMO directly or via the LEO Pharma A/S affiliate.

L E O

THIS DOCUMENT CONTAINS TRADE SECRETS, OR COMMERCIAL OR FINANCIAL INFORMATION, PRIVILEGED OR CONFIDENTIAL, DELIVERED IN CONFIDENCE AND RELIANCE THAT SUCH INFORMATION WILL NOT BE COPIED OR MADE AVAILABLE TO ANY THIRD PARTY WITHOUT THE WRITTEN CONSENT OF LEO PHARMA A/S - LEO PHARMACEUTICAL PRODUCTS LTD. A/S

10.6.8.2 Investigator-Subject Drug Accountability

An inventory (Individual Drug Accountability Form) will be kept of all investigational product given to each subject in the trial. This inventory must be available for inspection during the monitoring visits and will be checked by the monitor to ensure correct dispensing of investigational product.

10.6.8.3 End of Trial Drug Accountability

The used unit-dose tubes will be stored at the site until completion of accountability procedures and returned to the CMO for overall IP reconciliation and destruction after the trial is completed.

10.6.8.4 Treatment Compliance

Each dose of investigational product will be applied by the (sub)investigator using a gloved finger. This will be recorded on the CRF accordingly.

10.6.9 Prior and Concomitant Treatment

10.6.9.1 Prior to the Trial

Use of concomitant treatment should be recorded in the subject's medical record and the CRF (treatment/drug name, dose, indication and dates of start and stop). Please refer to section 10.4.2, Exclusion Criteria for prohibited medications prior to patient enrolment into the trial.

Use of non-marketed/other investigational products within in prior two months and during the trial is not permitted.

10.6.9.2 During the Trial

Prohibited concomitant treatment and procedures during the study are detailed below.
Use of concomitant treatment should be recorded in the subject's medical record and the CRF (treatment/drug name, dose, indication and dates of start and stop).

**Prohibited Treatment**	**Location**	**Exclusion Period Restrictions**
Cosmetic procedures (e.g.: use of liquid nitrogen, surgical extension, curettage, dermabrasion. Medium or great depth chemical heal, laser resurfacing	within 10 cm of the selected treatment areas	Anytime during the study

LEO

THIS DOCUMENT CONTAINS TRADE SECRETS, OR COMMERCIAL OR FINANCIAL INFORMATION, PRIVILEGED OR CONFIDENTIAL, DELIVERED IN CONFIDENCE AND RELIANCE THAT SUCH INFORMATION WILL NOT BE COPIED OR MADE AVAILABLE TO ANY THIRD PARTY WITHOUT THE WRITTEN CONSENT OF LEO PHARMA A/S - LEO PHARMACEUTICAL PRODUCTS LTD. A/S

Acid- containing therapeutic procedures (e.g., salicylic acid or fruit acid, such as α and β hydroxy acids and glycerol acids, topical retinoid or light chemical peels	Within 2 cm of the selected treatment areas	Anytime during the trial
Medicated/therapeutic topical salves (non-mediation/non-irritant salves are acceptable)	Within 2 cm of the selected treatment areas	Anytime during the study
Any medications or treatments that might influence the intended effects or mask the side effects of treatment, such as topical corticosteroids	Within 2 cm of the selected treatment areas	Anytime during the study
Artificial tanners	Within 5 cm of the selected treatment areas	Anytime during the study
Psoralen plus UVA (PUVA) or the use if UVB therapy	anywhere	Anytime during the study
5-FU, imiquimod. Diclofenac or photodynamic therapy	Anywhere	Anytime during the study
Immuno-modulators (e.g., azathioprine), cytotoxic drugs (e.g., cyclophosphamide, vinblastine, chlorambucil, methotrexate, podophyllin, camptothecin) or interferon/interferon includes	Excluded	Anytime during the study
Medications that suppress the immune system (e.g., cyclosporine, methotrexate, podophyllin, alefacept, infliximab)	Excluded	Anytime during the study
Systemic retinoids (e.g., isotretinoin, acitretin, bexarotene)	Excluded	Anytime during the study
Excessive or prolonged exposure to the ultraviolet light (e.g., sunlight, tanning booths)	Excluded	Anytime during the study
During the study, subjects must not receive any other investigational drugs, agents or any chemotherapy for cancer treatment or any medications or treatments that might influence the intended effects or mask the side effects of study medication.	Excluded	Anytime during the study

L E O

THIS DOCUMENT CONTAINS TRADE SECRETS, OR COMMERCIAL OR FINANCIAL INFORMATION, PRIVILEGED OR CONFIDENTIAL, DELIVERED IN CONFIDENCE AND RELIANCE THAT SUCH INFORMATION WILL NOT BE COPIED OR MADE AVAILABLE TO ANY THIRD PARTY WITHOUT THE WRITTEN CONSENT OF LEO PHARMA A/S - LEO PHARMACEUTICAL PRODUCTS LTD. A/S

Elective surgical procedures		defer until Day 15 after first treatment, if possible

10.7 TRIAL PROCEDURES

10.7.1 Schedule of Trial Procedures

	Screening	Visit 1	Visit 2	Visit 3	Visit 4	Visit 5
Day	-7 to 1	1	2	3	8 Follow up	29 End of Trial
**Visit window**					+/- 3 days	+/- 3 days
Informed consent	X					
Subject demographics	X					
In-/exclusion criteria	X	X				
Urine Pregnancy test[1)]	X	X			X	
Concomitant diagnoses	X					
Concomitant medication	X	X	X	X	X	X
Medical history	X	X				
Physical examination	X	X				
Biopsy		X	X[2)]	X		
Vital signs (BP, heart rate in supine position, oral body temperature)	X	X	X	X	X	X
LSR	X	X	X	X	X	X
Administration of trial medication by physician		X	X[3)]			
Photographs	X	X	X	X	X	X
Adverse Event(s)		X	X	X	X	X
End of Trial Form						X[4)]
1) For female of child-bearing potential a urine pregnancy test must be performed at the screening visit<br>2) Only on AK lesion within AK Treatment Area<br>3) Study medication application to be applied within 24 +/- 2 hours where possible. If subjects						

LEO

THIS DOCUMENT CONTAINS TRADE SECRETS, OR COMMERCIAL OR FINANCIAL INFORMATION, PRIVILEGED OR CONFIDENTIAL, DELIVERED IN CONFIDENCE AND RELIANCE THAT SUCH INFORMATION WILL NOT BE COPIED OR MADE AVAILABLE TO ANY THIRD PARTY WITHOUT THE WRITTEN CONSENT OF LEO PHARMA A/S - LEO PHARMACEUTICAL PRODUCTS LTD. A/S

	prematurely withdrawn from study medication every attempt should be made to have the subject attend all follow up visits.	
4)	If the subject is not enrolled the End of Trial form in eCRF should be completed	

10.7.2 Subject Eligibility

Subject's eligibility for the clinical trial will be checked according to the inclusion and exclusion criteria at the screening visit and Visit 1. Investigator assessments will be done according to section 10.7.3. Urine test will be performed according to section 10.7.4.2.

Relevant medical history (including first date of AK diagnosis) and concomitant diagnoses will be recorded at the screening visit and will be confirmed at Visit 1.

10.7.3 Clinical Assessment

All assessments for the subject must be performed by a (sub)investigator with dermatological expertise.

10.7.3.1 Investigator Assessments

The (sub)investigator will make the following clinical assessments:
Baseline Characteristics
Subjects' demographic details (date of birth, sex, race, ethnic origin) will be recorded. The subject will self-report their ethnicity (Hispanic or Latino, not Hispanic or Latino), and race (American Indian or Alaska Native; Asian, Black or African American; Native Hawaiian or Other Pacific Islander; White or Caucasian, Other).

Skin type of the subjects will be assessed and recorded according to the following classification:

Fitzpatrick Skin Types

- I Always burns easily, never tans
- II Always burns easily, tans minimally
- III Burns moderately, tans gradually (light brown)
- IV Burns minimally, always tans well (moderate brown)
- V Rarely burns, tans very well (moderate brown)
- VI Never burns, deeply pigmented

L E O
THIS DOCUMENT CONTAINS TRADE SECRETS, OR COMMERCIAL OR FINANCIAL INFORMATION, PRIVILEGED OR CONFIDENTIAL, DELIVERED IN CONFIDENCE AND RELIANCE THAT SUCH INFORMATION WILL NOT BE COPIED OR MADE AVAILABLE TO ANY THIRD PARTY WITHOUT THE WRITTEN CONSENT OF LEO PHARMA A/S - LEO PHARMACEUTICAL PRODUCTS LTD. A/S

Vital signs (blood pressure (diastolic and systolic), heart rate in supine position, oral body temperature) and concomitant medication will be recorded throughout the study.

Before the measurement of vital signs, the subject must rest for at least 5 minutes. All measurements should be performed using the same position (i.e. sitting or lying) and method throughout the trial for each individual subject (e.g. body temperature measured using an ear thermometer throughout the trial).

Identification and documentation of the selected treatment areas will be conducted at the screening visit and is outlined in Appendix III. The location of the selected treatment area will be verified by referencing the transparency on which it was mapped and the Screening photographs.

Local Skin Responses

The same (sub)investigator performing the Screening and Day 1 assessments should attempt to make all subsequent study assessments for each individual subject and across enrolled subjects.

The selected treatment area will be assessed for LSRs at each subsequent study visit. The presence/absence and grade of the following LSRs will be recorded:

- erythema
- flaking/scaling
- crusting
- swelling
- vesiculation/pustulation
- erosion/ulceration

Specific definitions for each LSR category can be found in the Local Skin Response Grading Scale in Appendix IV. Skin responses other than those identified and listed above, should be recorded appropriately as AEs. Any changes to the dosage schedule of the study medication or any corrective therapy as a result of a LSR must be recorded.

Physical Examination

A complete physical examination will include a full body systematic physical exam, as outlined below;

- General appearance
- Head, Eye, Ear, Nose and Throat Exam (HEENT)
- Respiratory

L E O
THIS DOCUMENT CONTAINS TRADE SECRETS, OR COMMERCIAL OR FINANCIAL INFORMATION, PRIVILEGED OR CONFIDENTIAL, DELIVERED IN CONFIDENCE AND RELIANCE THAT SUCH INFORMATION WILL NOT BE COPIED OR MADE AVAILABLE TO ANY THIRD PARTY WITHOUT THE WRITTEN CONSENT OF LEO PHARMA A/S - LEO PHARMACEUTICAL PRODUCTS LTD. A/S

- Cardiovascular
- Abdominal
- Neurological
- Musculoskeletal
- Lymph Nodes (including regional lymph node palpations)
- Skin
- Other

10.7.3.2 Subject assessments

Not applicable.

10.7.3.3 Imaging Assessments

Colour photographs of the treated areas (AK Treatment Area and Normal Skin Treatment Area) will be taken for all subjects on all visit days. The photographs are to be taken prior to study medication application at Day 1 and Day 2, and prior to the biopsy on Day 3. Photographs will be used for visual documentation of the treated area and corresponding LSRs. In addition, these photographs may be used to support the (sub)investigator's assessment of the treated area. Printed copies of the photographs must be included as part of individual subject source documentation. The site will provide the photographic equipment and site photograph will be in charge of taking the pictures in accordance to the site routine procedures.

10.7.4 Laboratory Assessments

10.7.4.1 Central Analysis

Histological Assessments: Biopsy

- Visit 1 (Day 1): 4 mm punch biopsies from one AK lesion and from Normal skin (1 to 5 cm outside the selected treatment areas) prior to any study medication application (baseline biopsy) will be taken.
- Visit 2 (Day 2): 4 mm punch biopsy from one AK lesion within the selected AK Treatment Area prior to Day 2 study medication application will be taken.
- Visit 3 (Day 3): 4 mm punch biopsies from one AK lesion and from Normal skin (within the selected treatment areas) will be taken.

The biopsy area will be cleaned with an appropriate disinfectant considering the eczematous nature of the subject’s skin. The skin will be numbed using an anesthetic injection with a

LEO

THIS DOCUMENT CONTAINS TRADE SECRETS, OR COMMERCIAL OR FINANCIAL INFORMATION, PRIVILEGED OR CONFIDENTIAL, DELIVERED IN CONFIDENCE AND RELIANCE THAT SUCH INFORMATION WILL NOT BE COPIED OR MADE AVAILABLE TO ANY THIRD PARTY WITHOUT THE WRITTEN CONSENT OF LEO PHARMA A/S - LEO PHARMACEUTICAL PRODUCTS LTD. A/S

vasoconstrictor, e.g. adrenaline. Biopsies will be taken using a 4-mm disposable punch. If possible, the punch should contain tissue from the subcutis. The biopsy specimen will be removed from the punch very gently using forceps. Care should be taken not to squeeze the biopsy, The biopsy is transferred immediately into a plastic tube of approximately 10 mL containing 8 mL 10% neutral buffered formalin (formalin [40% formaldehyde] diluted 10 times in phosphate buffered saline, ph 7.4). It should be assured that the biopsy is submerged completely in the fixative. Specimens will be labeled with the study number (LP0041-02), subject (CRF) number and visit/skin type code (using the blinded list). Hemostasis will be secured by pressure with gauze and stitching. The stitched biopsy site will be left uncovered or sealed with steristrip® as required. The formalin fixed tissue will be shipped to the site local lab to be assessed by the designated Investigator. The biopsies will be paraffin embedded, sectioned and the tissue section analyzed by immunohistochemistry. The sections will be stained with monoclonal antibodies.
The assessment on the biopsies includes (scoring in parentheses):

- Necrosis (+/-), haemorrhage (+/-) and inflammation (0-3), all H&E stain
- Apoptosis (0-3)
- Markers for leukocyte subsets. This will as a minimum include immunohistochemical staining for CD4, CD8, CD20, CD68, CD1a, and chloroacetateesterase (marker for neutrophils + mast cells)
- Markers for vascular endothelium activation, CD31
- Transcriptomics, mRNA and miRNA analysis. The transcriptomics analysis will use the following chips for analysis: Affymetrix; GeneChip® Human Gene 1.0 ST Array (28,869 human genes) and Exiqon miRCURY LNA™ microRNA Array, 6th gen - hsa (899 human microRNAs). Quantitative real time PCR will be used to validate findings (up till 20 mRNAs and 20 miRNAs)
- Classification of the AK lesions according the KIN I-III grading (i.e., I - dysplasia within the lower third of the epidermis; II - dysplasia within the lower two thirds of the epidermis; III - dysplasia throughout the epidermis)
- Classification of the AK lesions according to the different histological types (i.e. hypertrophic, acantholytic, bowenoid, lichenoid, atrophic) where appropriate.

The analysis will be performed by an investigator in batches after 8, 9 and 10 subjects are completed. All samples will be de-identified, i.e. the subject and visit day are not known when the analysis is performed. Once complete, the samples will be shipped to LEO Pharma, Denmark for a second round of analysis. Once this analysis is complete, the samples will be returned to the site for storage as per local requirements.

LEO
THIS DOCUMENT CONTAINS TRADE SECRETS, OR COMMERCIAL OR FINANCIAL INFORMATION, PRIVILEGED OR CONFIDENTIAL, DELIVERED IN CONFIDENCE AND RELIANCE THAT SUCH INFORMATION WILL NOT BE COPIED OR MADE AVAILABLE TO ANY THIRD PARTY WITHOUT THE WRITTEN CONSENT OF LEO PHARMA A/S - LEO PHARMACEUTICAL PRODUCTS LTD. A/S

10.7.4.2 Local Analysis

A urine pregnancy test will be performed at the trial site at the screening and visit 1 prior to enrolment, and at Visit 4, in female subjects of child-bearing potential. Test kits will be provided by LEO Pharma A/S.

10.7.5 Adverse Events

Any untoward event to a medicinal occurrence in a patient or clinical-trial subject administered a medicinal product which does not necessarily have to have a causal relationship with this treatment. An adverse event can therefore be any unfavourable and unintended sign (e.g. an abnormal laboratory finding), symptom, or disease temporally associated with the use of a medicinal product, whether or not considered related to medicinal product. (ICH Harmonized Tripartite Guideline for Good Clinical Practice, E6 (R1)).

A Serious Adverse Event (SAE) is any untoward medical occurrence that

- results in death
- is life-threatening
- requires inpatient hospitalisation or prolongation of existing hospitalisation
- results in persistent or significant disability/incapacity or
- is a congenital anomaly/birth defect

or

- other medically important conditions*)

*) Events that may not be immediately life-threatening or result in death or hospitalisation but may jeopardise the subject or may require intervention to prevent one of the other outcomes listed in the definition above. Examples of such events are allergic broncospasm, blood dyscrasias, and convulsions.

Global Pharmacovigilance, LEO Pharma A/S is responsible for the assessment of headquarter expectedness according to LEO Pharma A/S procedures. The relevant reference document for this clinical trial is Investigators Brochure LEO PEP005 Field Therapy Edition no. 1 and subsequent updates as agreed between the Head of International Clinical Development, and the Medical Director, Global Pharmacovigilance, LEO Pharma A/S.

L E O
THIS DOCUMENT CONTAINS TRADE SECRETS, OR COMMERCIAL OR FINANCIAL INFORMATION, PRIVILEGED OR CONFIDENTIAL, DELIVERED IN CONFIDENCE AND RELIANCE THAT SUCH INFORMATION WILL NOT BE COPIED OR MADE AVAILABLE TO ANY THIRD PARTY WITHOUT THE WRITTEN CONSENT OF LEO PHARMA A/S - LEO PHARMACEUTICAL PRODUCTS LTD. A/S

At all visits, the subject will be asked a non-leading question by the investigator: "How have you felt since I saw you last?" No specific symptoms will be asked for.

If there are no AEs to record, no further questions will be asked and "NO" should be stated. In case there are one or more AEs to record, then "YES" should be stated and the investigator will record the event term, intensity, duration, suspected causal relationship to the investigational product and outcome.

It is important that the investigator also observes the subject for any changes not reported by the subject, and records these changes.

Only medically qualified personnel must assess AEs.

10.7.5.1 Reporting of Adverse Events

Events reported by the subject, or observed by the (sub)investigator, that fall into any of the above definitions must be recorded on the adverse event page of the CRF from the time of informed consent. They should be described in the following manner:

The **nature** of the event will be described in precise, English medical terminology (i.e. not necessarily the exact words used by the subject). Whenever possible, a specific diagnosis should be stated (e.g. allergic contact dermatitis).

Both Adverse Events (AEs) and LSRs will be recorded.

Skin responses other than those identified in the LSR scale, should be recorded appropriately as AEs. Any changes to the dosage schedule of the study medication or any corrective therapy as a result of a LSR or AE must be recorded.

At each study visit, the selected treatment area will be assessed for LSRs which are to be reported under "Local Skin Responses" in the CRF and not reported as AEs.

Furthermore it will be recorded whether the AE started prior to or after first exposure to investigational product.

For cutaneous adverse events not considered as LSR, the location must be part of the adverse event description and may be described as exposed area or non-exposed area.

L E O

THIS DOCUMENT CONTAINS TRADE SECRETS, OR COMMERCIAL OR FINANCIAL INFORMATION, PRIVILEGED OR CONFIDENTIAL, DELIVERED IN CONFIDENCE AND RELIANCE THAT SUCH INFORMATION WILL NOT BE COPIED OR MADE AVAILABLE TO ANY THIRD PARTY WITHOUT THE WRITTEN CONSENT OF LEO PHARMA A/S - LEO PHARMACEUTICAL PRODUCTS LTD. A/S

The **intensity** of adverse events will be described in terms of mild, moderate or severe according to the investigator's clinical judgement.

- **Mild**. The adverse event does not interfere in a significant manner with the subject's normal functioning level and requires no medical intervention.
- **Moderate**. The adverse event interferes with the subject's normal functioning level and may or may not require medical intervention.
- **Severe**. The adverse event produces significant impairment of the subject's functioning or requires medical intervention.

The **duration** of the event will be reported as the start date and stop date of the event.

The **causal relation** of the event to the use of the investigational product will be described in terms of probable, possible, not related or not assessable according to the following:

Probably related

- Follows a reasonable temporal sequence from administration of the investigational product
- Could not be reasonably explained by the subject's clinical state, environmental or toxic factors or other therapies administered to the subject
- Follows a known pattern of response to the investigational product
- Disappears or decreases on cessation or reduction in dose of the investigational product
- Reappears or worsens upon re-challenge

Possibly related

- Follows a reasonable temporal sequence from administration of the investigational product
- Could also be reasonably explained by the subject's clinical state, environmental or toxic factors or other therapies administered to the subject
- Follows a known pattern of response to the investigational product.

Not related

- Does not follow a reasonable temporal sequence from administration of the investigational product

L E O
THIS DOCUMENT CONTAINS TRADE SECRETS, OR COMMERCIAL OR FINANCIAL INFORMATION, PRIVILEGED OR CONFIDENTIAL, DELIVERED IN CONFIDENCE AND RELIANCE THAT SUCH INFORMATION WILL NOT BE COPIED OR MADE AVAILABLE TO ANY THIRD PARTY WITHOUT THE WRITTEN CONSENT OF LEO PHARMA A/S - LEO PHARMACEUTICAL PRODUCTS LTD. A/S

- Is better explained by other factor like the subject's clinical state, environmental or toxic factors or other therapies administered to the subject
- Does not follow a known pattern of response to the investigational product.

Not assessable

- The adverse event cannot yet be judged otherwise because present information is insufficient or contradictory. A final assessment (i.e. probably, possibly, or not related) shall be made as more information becomes available, at the latest when the subject has completed the trial.

The **outcome** of the event will be classified and handled as follows:

• Recovered/resolved	The event has stopped. The stop date of the event must be recorded
• Recovering/resolving	The subject is clearly recovering from an event. The event is, however, not yet completely resolved. Follow-up on the event is required until final outcome is established
• Not recovered/not resolved	Event is still ongoing. Follow-up on the event is required until final outcome is established
• Recovered with sequelae	The event has reached a state where no further changes are expected and the residual symptoms are assumed to persist. An example is hemiparesis after stroke. The stop date of the event must be recorded
• Fatal	The subject has died as a consequence of the event. Date of death is recorded as stop date for the adverse event
• Unknown	Unknown to (sub)investigator, e.g. subject lost to follow-up

Once a subject has completed the clinical trial, the investigator should follow-up for outcome on all non-Serious Adverse Events classified as possibly/probably related to the investigation-

L E O
THIS DOCUMENT CONTAINS TRADE SECRETS, OR COMMERCIAL OR FINANCIAL INFORMATION, PRIVILEGED OR CONFIDENTIAL, DELIVERED IN CONFIDENCE AND RELIANCE THAT SUCH INFORMATION WILL NOT BE COPIED OR MADE AVAILABLE TO ANY THIRD PARTY WITHOUT THE WRITTEN CONSENT OF LEO PHARMA A/S - LEO PHARMACEUTICAL PRODUCTS LTD. A/S

al product or not assessable until resolution or the follow-up assessment, whichever comes first.

10.7.5.2 Other events to be reported

Pregnancy

Pregnancy which occurs during a clinical trial with an investigational product must be reported to LEO Pharma A/S within one calendar day of first knowledge using the Pregnancy Follow-up Form supplied by LEO Pharma A/S. All pregnancies must be followed-up until delivery or termination.

Please also confer with chapter 10.5, withdrawal criteria.

Overdose

Any overdose defined as any higher dose than prescribed for the individual subject must be reported on the adverse event form of the CRF book. AEs originating in the overdose must be documented on a separate line.

Aggravation of condition

Any clinically significant aggravation/exacerbation/worsening of the initially treated condition compared to baseline, judged by an overall medical assessment, must be reported as an AE.

10.7.6 Serious adverse events

10.7.6.1 Reporting of Serious Adverse Events

Any Serious Adverse Event (SAE), related or unrelated to the investigational product or any trial procedure after signature of the Informed Consent Form must be reported to LEO Pharma A/S on the (paper) **Serious Adverse Event Form – Clinical Trial** within **one calendar day of first knowledge**.

Note: Planned hospitalisation or planned prolonged hospitalisation does not fulfil the criteria for being an SAE. The elective nature of the event must be clearly documented in the subject’s medical record.

L E O
THIS DOCUMENT CONTAINS TRADE SECRETS, OR COMMERCIAL OR FINANCIAL INFORMATION, PRIVILEGED OR CONFIDENTIAL, DELIVERED IN CONFIDENCE AND RELIANCE THAT SUCH INFORMATION WILL NOT BE COPIED OR MADE AVAILABLE TO ANY THIRD PARTY WITHOUT THE WRITTEN CONSENT OF LEO PHARMA A/S - LEO PHARMACEUTICAL PRODUCTS LTD. A/S

An LSR fulfilling the serious criteria should be reported as a SAE. The event must be added to the adverse event form of the CRF book and reported to LEO on the SAE form.

SAEs must be reported on the adverse event form of the CRF book. Additionally reports must be made using the paper Serious Adverse Event Form – Clinical Trial**,** supplied by LEO Pharma A/S. Apart from the assessment of the intensity, causal relationship to the investigational product(s) and/or trial procedures, the action taken and the outcome to date, this report must contain a comprehensive narrative description of the course of the event.

The completed Serious Adverse Event Form – Clinical Trial must be faxed or scanned and emailed to the local LEO Pharma A/S affiliate.

All other relevant reports of diagnostic procedures, hospital records, autopsy reports etc. must be included, as applicable or upon request from Global Pharmacovigilance.

The IRB(s)/IEC(s), regulatory authorities and concerned (sub)investigators will be notified of SAEs according to current regulation and local requirements.

All Suspected, Unexpected Serious Adverse Reactions (SUSARs) are subject to expedited reporting to regulatory authorities. Global Pharmacovigilance will un-blind such cases prior to reporting. Investigators will remain blinded.

SAEs must be followed indefinitely until a final outcome has been established, i.e. the follow-up may continue beyond the end of the clinical trial.

10.7.7 Source Data

Source data*: All information in original records and certified copies of original records of clinical findings, observations, or other activities in a clinical trial necessary for the reconstruction and evaluation of the trial. Source data are contained in source documents (original records or certified copies).* (ICH E6, 1.51).

Source documents*: Original documents, data, and records (e.g., hospital records, clinical and office charts, laboratory notes, memoranda, subjects diaries or evaluation checklists, pharmacy dispensing records, recorded data from automated instruments, copies or transcriptions certified after verification as being accurate copies, microfiches, photographic negatives, microfilm or magnetic media, x-rays, subject files, and records kept at the pharma-*

L E O
THIS DOCUMENT CONTAINS TRADE SECRETS, OR COMMERCIAL OR FINANCIAL INFORMATION, PRIVILEGED OR CONFIDENTIAL, DELIVERED IN CONFIDENCE AND RELIANCE THAT SUCH INFORMATION WILL NOT BE COPIED OR MADE AVAILABLE TO ANY THIRD PARTY WITHOUT THE WRITTEN CONSENT OF LEO PHARMA A/S - LEO PHARMACEUTICAL PRODUCTS LTD. A/S

cy, at the laboratories and at medico-technical departments involved in the clinical trial). (ICH E6, 1.52).

Source Data Verification (SDV) is a key function in assuring the sponsor that clinical trial information is recorded and handled in a way that allows its accurate reporting, interpretation and verification. Monitors will, during the conduct of the clinical trial, perform SDV to confirm the accuracy and completeness of CRFs by verifying selected (as specified below) data recorded in the CRF against data recorded in source documents to ensure such records are consistent.

To enable SDV it is essential, that what constitutes source data/documents (see definition above) for the clinical trial data to be collected in the CRF as well as where such data can be found at the trial site is established and agreed with the (sub)investigator at each trial site and documented, prior to initiation of the clinical trial.

Source data cannot be entered directly into the CRF. Source data should as a general rule be recorded in the subject's medical record or other defined document normally used at the trial site. Source data may be entered on a worksheet only if the clinical trial requires capture of data, which are normally not part of the subject's medical record.

For this clinical trial, the all parameters collected in the CRF should be verifiable from source documents available at the trial site.

In addition to the above, the following should be added to the subject's medical record, in chronological order, i.e. when these are allocated to the subject.

- Date(s) of conducting the informed consent process including date of provision of subject information
- Date of enrolment
- Patient CRF Book Number
- The fact that the subject is participating in a clinical trial in LP0041-02 and will be exposed to PEP005 Gel, 0.05%.

10.8 EFFICACY EVALUATION

Not applicable.

L E O
THIS DOCUMENT CONTAINS TRADE SECRETS, OR COMMERCIAL OR FINANCIAL INFORMATION, PRIVILEGED OR CONFIDENTIAL, DELIVERED IN CONFIDENCE AND RELIANCE THAT SUCH INFORMATION WILL NOT BE COPIED OR MADE AVAILABLE TO ANY THIRD PARTY WITHOUT THE WRITTEN CONSENT OF LEO PHARMA A/S - LEO PHARMACEUTICAL PRODUCTS LTD. A/S

10.9 SAFETY EVALUATION

Reported adverse events and their seriousness, intensity and causality will be assessed for the safety evaluation.
Evaluation of vital signs, reasons for withdrawal and withdrawal rate as well as Local Skin Response (LSR) will also be recorded.

10.9.1 Evaluation of (Serious) Adverse Events

All serious adverse events will be reported.

10.9.2 Evaluation of Laboratory Data

The following analysis will be conducted on the 5 biopsies performed on each subject.

1. Degree of skin infiltration of leukocytes in haematoxylin & eosin-stained sections assessed on a scale from 0 to 3 in the AK biopsies from Day 1, Day 2, and Day 3
2. Degree of necrosis of the epidermis and dermis in haematoxylin & eosin-stained sections assessed as binary outcome (present/absent) in the AK biopsies from Day 1, Day 2, and Day 3
3. Degree of skin infiltration of leukocytes in haematoxylin & eosin-stained sections assessed on a scale from 0 to 3 in the normal skin biopsies from day 3 compared to baseline and to AK lesion biopsies
4. Degree of necrosis of the epidermis and dermis in haematoxylin & eosin-stained sections assessed as binary outcome (present/absent) in the normal skin biopsies from day 3 compared to baseline and to AK lesion biopsies
5. Degree of haemorrhage assessed by histology (binary outcome present/absent) in the Normal skin and AK lesion biopsies at all time points
6. Characterisation of immune cells such as T and B lymphocytes, polymorphonuclear cells, mast cells and antigen presenting cells in the Normal skin and AK lesion biopsies
7. Degree of apoptosis in the Normal skin and AK lesion biopsies
8. Degree of vascular endothelium activation in the Normal skin and AK lesion biopsies
9. Changes in mRNA and miRNA expression in the Normal skin and AK lesion biopsies
10. Expression of drug transporters in the Normal skin and AK lesion biopsies (P-gP)

10.9.3 Evaluation of Other Observations

Not applicable

L E O
THIS DOCUMENT CONTAINS TRADE SECRETS, OR COMMERCIAL OR FINANCIAL INFORMATION, PRIVILEGED OR CONFIDENTIAL, DELIVERED IN CONFIDENCE AND RELIANCE THAT SUCH INFORMATION WILL NOT BE COPIED OR MADE AVAILABLE TO ANY THIRD PARTY WITHOUT THE WRITTEN CONSENT OF LEO PHARMA A/S - LEO PHARMACEUTICAL PRODUCTS LTD. A/S

10.10 STATISTICAL ANALYSIS

General Statistical Methodology

Prior to database lock, a final detailed statistical analysis plan (SAP) will be available. Study results will be summarised into tabulations, listings and figures where appropriate.

Analyses of the histological results will be based on data from all patients that have received at least one dose of study medication and have had at least one biopsy taken from a treated area.

Safety analyses will be based on the safety population, which is defined as all patients that have received at least one dose of study medication and have at least one post-baseline safety assessment.

Statistical Hypothesis

Twenty-seven patients should be enrolled into this study to obtain an adequate precision. There is no formal statistical hypothesis to be evaluated.

The primary objective is to describe the biological effects of PEP005 in the skin. The degree of skin infiltration of leukocytes and the degree of necrosis of the epidermis and dermis will be presented in the AK biopsies at Day 1, 2 and 3 graphically and in tabular format.

The secondary objective is to compare the skin response between normal and AK treatment areas within subjects. Data will be presented graphically and as cross-tables comparing the distribution of the endpoints assessed at the normal and AK treated areas.

Adverse Events and Serious Adverse Events

The (sub)investigator’s verbatim term of each adverse event will be mapped to system organ class (SOC) and preferred terms using the current version of the Medical Dictionary for Regulatory Activities (MedDRA) dictionary.

An overview summary of the number (percentage) of patients with any treatment emergent adverse events (TEAEs), serious adverse events (SAEs), premature discontinuations from dosing or the study due to adverse events, treatment related AEs, severe AEs (maximum intensity indicated as severe on the CRF) and events of abnormal proliferation will be presented. The overall AE summary will also be presented by number of doses received.

L E O

THIS DOCUMENT CONTAINS TRADE SECRETS, OR COMMERCIAL OR FINANCIAL INFORMATION, PRIVILEGED OR CONFIDENTIAL, DELIVERED IN CONFIDENCE AND RELIANCE THAT SUCH INFORMATION WILL NOT BE COPIED OR MADE AVAILABLE TO ANY THIRD PARTY WITHOUT THE WRITTEN CONSENT OF LEO PHARMA A/S - LEO PHARMACEUTICAL PRODUCTS LTD. A/S

The number and percent of patients with AEs will be tabulated by body system and preferred term for the above summaries. Within a specific body system or preferred term, the patient is to be counted only once if more than one event is reported. Summaries will be presented by decreasing frequency.

Listings will be provided for all patients with SAEs and study discontinuations due to adverse experiences.

LSR's will be converted into MedDRA preferred terms applying the following conversion table. These adverse events will be reported separately.

LSR Term	LSR Grade	MedDRA code in Preferred Term
Erythema	1-4	Application site erythema
Flaking/Scaling	1-4	Application site exfoliation.
Crusting	1-4	Application site scab
Swelling	1-4	Application site swelling
Vesiculation /Pustulation	1	Application site blister
	2-4	Application site pustules
Erosion/ Ulceration	1-3	Application site erosion
	4	Application site ulcer

Local Skin Response
Assessment of LSRs will be presented by skin response at each study visit. Local skin response grades will be considered as ordered variables and presented using descriptive statistics. For each of the six components of LSR: erythema, flaking/scaling, crusting,

L E O
THIS DOCUMENT CONTAINS TRADE SECRETS, OR COMMERCIAL OR FINANCIAL INFORMATION, PRIVILEGED OR CONFIDENTIAL, DELIVERED IN CONFIDENCE AND RELIANCE THAT SUCH INFORMATION WILL NOT BE COPIED OR MADE AVAILABLE TO ANY THIRD PARTY WITHOUT THE WRITTEN CONSENT OF LEO PHARMA A/S - LEO PHARMACEUTICAL PRODUCTS LTD. A/S

swelling, vesiculation/pustulation and erosion/ulceration, change in grading across the 4 weeks will be tabulated and presented, irrespective of patients.
A composite score of the sum of the individual subject skin responses will also be presented. Changes in LSR grade from baseline will be presented at each scheduled visit.

Data will be monitored batch-wise in order to evaluate the feasibility of the performed assays.

10.11 TRIAL COMMITTEES

Not applicable.

10.12 QUALITY ASSURANCE/AUDIT

LEO Pharma A/S has implemented a system of quality assurance, including all elements described in this protocol. Within this system company Standard Operating Procedures (SOPs) are implemented to ensure that clinical trials are conducted in compliance with regulatory requirements and Good Clinical Practice. Quality control is applied to each stage of data handling to ensure that data are accurate, reliable and processed correctly.

Trial sites, facilities, laboratories and all data (including sources) and documentation must be available for GCP audit by LEO Pharma A/S or inspection by competent authorities.

Any aspect of the clinical trial may be subject to audit by LEO Pharma A/S and/or inspection by regulatory authorities (national or foreign) or IEC/IRB. Such audits/inspections may take place at the sponsor's site(s) or at any trial site including laboratories, pharmacies etc.

The monitor will, in case of audit, announce this in advance to the (sub)investigator and be present at the particular trial site during the audit.

The site staff should assist in all aspects of audit/inspection.

10.12.1 Trial Monitoring

LEO Pharma A/S, as sponsor of this clinical trial, is responsible to the regulatory authorities for assuring the proper conduct of the clinical trial with regard to protocol adherence and validity of the data recorded in the CRFs. The company has therefore assigned persons to monitor this trial. Their duties are to serve as the principal link between (sub)investigators and LEO Pharma A/S and advise the (sub)investigator on the collection and maintenance of accurate, complete, legible, well organised, and easily retrievable data for the clinical trial. In

L E O
THIS DOCUMENT CONTAINS TRADE SECRETS, OR COMMERCIAL OR FINANCIAL INFORMATION, PRIVILEGED OR CONFIDENTIAL, DELIVERED IN CONFIDENCE AND RELIANCE THAT SUCH INFORMATION WILL NOT BE COPIED OR MADE AVAILABLE TO ANY THIRD PARTY WITHOUT THE WRITTEN CONSENT OF LEO PHARMA A/S - LEO PHARMACEUTICAL PRODUCTS LTD. A/S

addition, they will explain to the (sub)investigators any aspect of the (conduct of the) clinical trial, including interpretation of the protocol, the purpose of collecting the specified data and reporting responsibilities.

In order to perform their role effectively, monitors and persons involved in quality assurance and inspections (see above) will need direct access to primary subject data, e.g. medical records, laboratory reports, appointment books, etc. Because this affects the subject's confidentiality, this fact is included on the Subject Information Sheet and Informed Consent Form.

This clinical trial is organised by LEO Pharma A/S and all enquiries should be made to a member of LEO Pharma A/S staff (see section 6.3, LEO Pharma A/S Personnel).

10.13 CASE REPORT FORM BOOKS AND DATA HANDLING

10.13.1 Case Report Forms (CRFs)

In this clinical trial data will be collected by means of Remote Data Capture (RDC). The (sub)investigator or staff authorised by the (sub)investigator will enter subject data into electronic CRFs designed by LEO Pharma A/S. A uniquely numbered CRF book will be used for each subject enrolled. Data recorded in the electronic CRFs will be accessible to site staff through a secure internet connection immediately after entry. The CRFs must be maintained in an up-to-date condition at all times by the (sub)investigator.

The investigator, or sub-investigator(s) authorised by the investigator, will electronically sign all sections of CRFs used. This signature information (incl. date of signature) will be kept in the audit trail and cannot be unaltered. Only medically-qualified (sub)investigators can sign data on clinical assessments/safety. Any correction(s) made by the investigator, or authorised site staff, to the CRF after original entry will be documented in the audit trail. Changes to data already approved, requires the re-signature of investigator or authorised staff. The person making the change and the date, time and reason for the change will be identified in the audit trail.

The trial monitor will check the CRFs for accuracy and completeness and perform source data verification preferably no later than six weeks after each subject visit. For archiving purposes each investigator will be supplied with a copy of the CRFs, for all subjects enrolled at the trial site, via an electronic medium at completion of the trial. Audit trail information will be

L E O
THIS DOCUMENT CONTAINS TRADE SECRETS, OR COMMERCIAL OR FINANCIAL INFORMATION, PRIVILEGED OR CONFIDENTIAL, DELIVERED IN CONFIDENCE AND RELIANCE THAT SUCH INFORMATION WILL NOT BE COPIED OR MADE AVAILABLE TO ANY THIRD PARTY WITHOUT THE WRITTEN CONSENT OF LEO PHARMA A/S - LEO PHARMACEUTICAL PRODUCTS LTD. A/S

included. CRFs will be available for inspection by authorised representatives from LEO Pharma A/S (e.g. audit by the quality assurance department), from regulatory authorities and/or IEC/IRBs.

10.13.2 Data Handling

Subject data should be entered into the electronic CRF in a timely manner by authorised site staff. Data will be entered by site staff and systematic data validation will be performed through the discrepancy management system within the data collection software. Queries for discrepant data may be generated automatically by the system upon entry or generated manually by the monitor or the study data manager. All queries, whether generated by the system or by a user, will be in an electronic format. This systematic validation will ensure that a clean and consistent database is provided prior to the statistical analysis being performed.

10.14 PROTOCOL AMENDMENTS

Neither the investigator(s) nor LEO Pharma A/S will change the Clinical Study Protocol without written agreement between LEO Pharma A/S and the International Co-ordinating Investigator. Any modification considered substantial requires approval/favourable opinion by the appropriate regulatory authority and IEC/IRB.

Protocol amendments are issued as Consolidated Clinical Study Protocols comprising all current amendments. Consolidated Clinical Study Protocols become effective when written approval has been provided by the International Co-ordinating Investigator, the Head of International Clinical Development, and the Head of Biostatistics and Data Management, LEO Pharma A/S and approval/favourable opinion from regulatory authorities and/or IEC/IRB has been obtained, as required.

Alternatively, a protocol addendum may be issued to comply with national/regional specific requirements. A protocol addendum becomes effective when written approval has been provided by the International Co-ordinating Investigator, the Head of International Clinical Development and the Head of Biostatistics and Data Management, LEO Pharma A/S and the and approval/favourable opinion from the relevant regulatory authorities and/or IEC/IRB has been obtained, as required.

L E O

THIS DOCUMENT CONTAINS TRADE SECRETS, OR COMMERCIAL OR FINANCIAL INFORMATION, PRIVILEGED OR CONFIDENTIAL, DELIVERED IN CONFIDENCE AND RELIANCE THAT SUCH INFORMATION WILL NOT BE COPIED OR MADE AVAILABLE TO ANY THIRD PARTY WITHOUT THE WRITTEN CONSENT OF LEO PHARMA A/S - LEO PHARMACEUTICAL PRODUCTS LTD. A/S

10.15 COMPLETION OF TRIAL

10.15.1 Trial Completion Procedures

The end of trial will be the date of the last subject's last visit. Investigators will be informed when subject recruitment is to cease.

Trial enrolment will be stopped at a trial site when the total requested number of subjects for the clinical trial has been obtained, irrespective of the specific site's planned inclusion number.

Upon completion of the clinical trial, LEO Pharma A/S will undertake arrangements for collection and disposal of any unused trial material that the investigator is not required to keep in his/her files.

LEO Pharma A/S may stop the clinical trial prematurely after consultation with the International Co-ordinating Investigator, e.g. if the subject recruitment is so slow that the clinical trial cannot be completed within a reasonable time frame. Such premature termination/suspension of the trial will be notified to regulatory authorities and IECs/IRBs, as required.

10.15.2 Provision for Subject Care Following Trial Completion

After the completion of the trial, the subjects will be treated at the investigator's discretion or referred to other physician(s), according to standard practice.

10.15.3 Archiving of Trial Documents

The investigator at each trial site must make arrangements to store the essential trial documents, (as defined in Essential Documents for the Conduct of a Clinical Trial (ICH E6, Guideline for Good Clinical Practice)) including the Investigator Trial File, until LEO Pharma A/S informs the investigator that the documents are no longer to be retained.

In addition, the investigator is responsible for archiving of all relevant source documents so that the trial data can be compared against source data after completion of the trial (e.g. in case of inspection from regulatory authorities).

L E O

THIS DOCUMENT CONTAINS TRADE SECRETS, OR COMMERCIAL OR FINANCIAL INFORMATION, PRIVILEGED OR CONFIDENTIAL, DELIVERED IN CONFIDENCE AND RELIANCE THAT SUCH INFORMATION WILL NOT BE COPIED OR MADE AVAILABLE TO ANY THIRD PARTY WITHOUT THE WRITTEN CONSENT OF LEO PHARMA A/S - LEO PHARMACEUTICAL PRODUCTS LTD. A/S

The investigator is required to ensure the continued storage of the documents, even if the investigator, for example, leaves the clinic/practice or retires before the end of required storage period.

At present according to ICH Guideline:

Essential documents should be retained until at least 2 years after last approval of a marketing application in an ICH region and until there are no pending or contemplated marketing applications in an ICH region or at least 2 years have elapsed since the formal discontinuation of clinical development of the investigational product. These documents should be retained for a longer period however if required by applicable regulatory requirements or by an agreement with the Sponsor. ***It is the responsibility of the Sponsor to inform the investigator/institution as to when these documents no longer need to be retained.*** (ICH E6, 4.9.5)

11 USE OF INFORMATION

This Clinical Study Protocol as well as all other information, data and results relating to this clinical trial and/or to the investigational product(s) is confidential information of LEO Pharma A/S and shall not be used by the investigator for purposes other than this clinical trial.

The investigator agrees that LEO Pharma A/S may use any and all information, data and results from this clinical trial in connection with the development of the investigational product(s), and therefore, may disclose and/or transfer information, data and/or results to other investigators, regulatory authorities and/or commercial partners.

12 PUBLICATION

Basic information of this clinical trial will be posted on the website: www.clinicaltrials.gov before the first subject enters into the clinical trial.

It is the intent that the results of this trial shall be published by Investigator and Sponsor in a joint publication following the Vancouver rules on authorships.

Prior to submitting or presenting a manuscript and/or presentation relating to the Study to a publisher, reviewer, or other outside person, the Sponsor shall have sixty (60) days to review and comment. Upon request of Sponsor the Investigator shall remove any Confidential Information of Sponsor (other than results generated by the Investigator) prior to submitting

L E O

THIS DOCUMENT CONTAINS TRADE SECRETS, OR COMMERCIAL OR FINANCIAL INFORMATION, PRIVILEGED OR CONFIDENTIAL, DELIVERED IN CONFIDENCE AND RELIANCE THAT SUCH INFORMATION WILL NOT BE COPIED OR MADE AVAILABLE TO ANY THIRD PARTY WITHOUT THE WRITTEN CONSENT OF LEO PHARMA A/S - LEO PHARMACEUTICAL PRODUCTS LTD. A/S

or presenting the manuscript and/or presentation. The Investigator shall, upon the request of Sponsor, delay the publication and/or presentation for a period up to one hundred and twenty (120) days to allow Sponsor to protect its Inventions and other intellectual property rights described in any such manuscripts and/or presentation.

In case the joint publication is still on-going and has not been made public at the time of notification of a publication and/or presentation made by the Investigator, Sponsor may also delay the publication and/or presentation made by the Investigator if the manuscript and/or presentation is deemed to harm the ongoing joint publication.
Also in case of publications and/or presentations made by the Investigator after the Sponsor publication has been published, the above mentioned notification requirements must be followed.

LEO Pharma A/S also subscribes to the Joint Position of the innovative pharmaceutical industry (35) for public disclosure of clinical trial results in a free, publicly accessible database, irregardless of outcome.

L E O
THIS DOCUMENT CONTAINS TRADE SECRETS, OR COMMERCIAL OR FINANCIAL INFORMATION, PRIVILEGED OR CONFIDENTIAL, DELIVERED IN CONFIDENCE AND RELIANCE THAT SUCH INFORMATION WILL NOT BE COPIED OR MADE AVAILABLE TO ANY THIRD PARTY WITHOUT THE WRITTEN CONSENT OF LEO PHARMA A/S - LEO PHARMACEUTICAL PRODUCTS LTD. A/S

13 REFERENCES

1. Cohen JL. Actinic keratosis treatment as a key component of preventive strategies for noncelanoma skin cancer. J Clin Aesthetic Dermatol 2010 Jun; 3(6): 39-44.

2. Burge SM, Bristol M, Millard PR, Dawber RP. Pigment changes in human skin after cryotherapy. Cryobiology 1986 Oct;23(5): 422-32.

3. Zouboulis CC. Cryosurgery in dermatology. European J Dermatol 1998 Oct-Nov;8(7):466-74.

4. Dinehart SM. Treatment of actinic keratosis. J Am Acad Dermatol 2000 Jan;42(1 Pt 2):S25-8.

5. Efudex (fluorouracil) Package Insert (FDA-approved). 2005, Valeant Pharmaceuticals North America.

6. Fluorplex (fluorouracil) Package Insert (FDA-approved). 2004, Allergan, Inc.

7. Carac Cream 0.5% (fluorouracil cream) Package Insert (FDA-approved). 2006, Dermik Laboratories, Sanofi-Aventis U.S. LLC.

8. Efudix (fluorouracil) Patient Information Leaflet (EU-approved). 2009, Meda Pharmaceuticals.

9. Solaraze Gel (diclofenac sodium 3%) Package Insert (FDA-approved). 2008, PharmaDerm.

10. Solaraze Gel (diclofenac sodium 3%) Package Leaflet (EU-approved). 2010, Almirall, S.A.

11. Aldara Cream, 5% (imiquimod) Package Insert (FDA-approved). 2009, Graceway Pharmaceuticals, LLC.

12. Zyclara Cream, 3.75% (imiquimod) Package Insert (FDA-approved). 2010, Graceway Pharmaceuticals, LLC.

L E O

THIS DOCUMENT CONTAINS TRADE SECRETS, OR COMMERCIAL OR FINANCIAL INFORMATION, PRIVILEGED OR CONFIDENTIAL, DELIVERED IN CONFIDENCE AND RELIANCE THAT SUCH INFORMATION WILL NOT BE COPIED OR MADE AVAILABLE TO ANY THIRD PARTY WITHOUT THE WRITTEN CONSENT OF LEO PHARMA A/S - LEO PHARMACEUTICAL PRODUCTS LTD. A/S

13. Aldara Cream, 5% (imiquimod) PackageLeaflet (EU-approved). 2008, 3M Health Care Limited.

14. Einspahr JG, Stratton SP, Bowden GT, et al. Chemoprevention of human skin cancer. Crit Rev Oncol Hematol 2002 Mar;41(3): 269-285.

15. Esmann S, Jemec GB. Management of actinic keratosis patients: a qualitative study. J Dermatolog Treat 2007;18(1): 53-8.

16. Thai KE, Fergin P, Freeman M, Vinciullo C, Fancis D, Spelman L, et al. A prospective study of the use of cryosurgery for the treatment of actinic keratoses. Int J Dermatol 2004 Sep;43(9): 687-92.

17. Holmes C, Foley P, Freeman M, Chong AH. Solar keratosis: epidemiology, pathogenesis, presentation, and treatment. Australas J Dermatol 2007 May;48(2): 67-76.

18. Alam M. Actinic keratoses: prevalence, pathogenesis, presentation, and prevention. Adv Stud Med 2006;6(8A): S785-90.

19. Ibrahim SF, Brown MD. Actinic keratoses: a comprehensive review. J Clin Aesthetic Dermatol 2009 Jul;2(7): 43-8.

20. Memon A, Tomenson JA, Bothwell J, Friedmann PS. Prevalence of solar damage andactinic keratosis in a Merseyside population. Br J Dermatol 2000 Jun; 142(6): 1154-9.

21. Babilas P, Landthaler M, Szeimies RM. Actinic kerotoses. Hautarzt 2003 Jun; 54(6): 551-60.

22. Fu W, Cockerell CJ. The actinic (solar) keratosis: A 21st century perspective. Arch Dermatol 2003 Jan;139(1): 66-70.

23. Ortonne JP. From actinic keratosis to squamous cell carcinoma. Br J Dermatol 2002 Apr;146 Suppl 61: 20-3.

24. Padilla RS, Sebastian S, Jiang Z, Nindl I, Larson R. Gene expression patterns of normal human skin, actinic keratosis, and squamous cell carcinoma: a spectrum of disease progression, Arch Dermatol 2010 Mar; 146(3): 288-293.

LEO

THIS DOCUMENT CONTAINS TRADE SECRETS, OR COMMERCIAL OR FINANCIAL INFORMATION, PRIVILEGED OR CONFIDENTIAL, DELIVERED IN CONFIDENCE AND RELIANCE THAT SUCH INFORMATION WILL NOT BE COPIED OR MADE AVAILABLE TO ANY THIRD PARTY WITHOUT THE WRITTEN CONSENT OF LEO PHARMA A/S - LEO PHARMACEUTICAL PRODUCTS LTD. A/S

25. Ogbourne SM, Suhrbier A, Jones B, Cozzi SJ, Boyle GM, Morris M, et al. Antitumour activity of 3-ingenyl angelate: Plasma membrane and mitochondrial disruption and necrotic cell death. Cancer Res 2004 Apr 15;64(8): 2833-9.

26. Hampson P, Chahal H, Khanim F, Hayden R, Mulder A, Assi LK, et al. PEP005, a selective small-molecule activator of protein kinase C, has potent antileukemic activity mediated via the delta isoform of PKC. Blood 2005 Aug 15;106(4): 1362-8.

27. Kedei N, Lundberg DJ, Toth A, Welburn P, Garfield SH, Blumberg PM. Characterization of the interaction of ingenol 3-angelate with protein kinase C. Cancer Res 2004 May 1;64(9): 3243-55.

28. Cozzi SJ, Parsons PG, Ogbourne SM, Pedley J, Boyle GM. Induction of senescence in diterpene ester-treated melanoma cells via protein kinase C-dependent hyperactivation of the mitogen-activated protein kinase pathway. Cancer Res 2006 Oct 15;66(20): 10083-91.

29. Hampson P, Kavanagh D, Smith E, Wang K, Lord JM, Rainger G. The anti-tumor agent, ingenol-3-angelate (PEP005), promotes the recruitment of cytotoxic neutrophils by activation of vascular endothelial cells in the PKCδ dependent manner. Cancer Immunol Immunother 2008 Aug;57(8): 1241-51.

30. Serova M, Ghoul A, Benhadji KA, Faivre S, Le Tourneau C, Cvitkovic E, et al. Effects of protein kinase C modulation by PEP005, a novel ingenol angelate, on mitogen-activated protein kinase and phosphatidylinositol 3-kinase signaling in cancer cells. Mol Cancer Ther 2008 Apr;7(4): 915-22.

31. Olsnes AM, Ersvær E, Ryningen A, Paulsen K, Hampson P, Lord JM, et al. The protein kinase C agonist PEP005 increases NF-kappaB expression, induces differentiation and increases constitutive chemokine release by primary acute myeloid leukaemia cells. Br J Haematol 2009 Jun;145(6): 761-74.

32. Challacombe JM, Suhrbier A, Parsons PG, Jones B, Hampson P, Kavanagh D, et al. Neutrophils are a key component of the antitumor efficacy of topical chemotherapy with ingenol-3-angelate. J Immunol 2006 Dec 1;177(11): 8123-32.

33. Riviere, JE. Comparative Pharmacokinetics: Principles, Techniques, and Applications. Ames, IA: Iowa State Press; 1999:36.

34. Klaassen, C. Casarett and Doull's Toxicology: The Basic Science of Poisons. New York, NY: Pergamon Press; 1991:61.

L E O

THIS DOCUMENT CONTAINS TRADE SECRETS, OR COMMERCIAL OR FINANCIAL INFORMATION, PRIVILEGED OR CONFIDENTIAL, DELIVERED IN CONFIDENCE AND RELIANCE THAT SUCH INFORMATION WILL NOT BE COPIED OR MADE AVAILABLE TO ANY THIRD PARTY WITHOUT THE WRITTEN CONSENT OF LEO PHARMA A/S - LEO PHARMACEUTICAL PRODUCTS LTD. A/S

35. Joint Position on the Disclosure of Clinical Trial Registries and Databases, available from: http://www.ifpma.org/Documents/NR10990/Revised_Joint_Industry_Position_26Nov08.pdf

THIS DOCUMENT CONTAINS TRADE SECRETS, OR COMMERCIAL OR FINANCIAL INFORMATION, PRIVILEGED OR CONFIDENTIAL, DELIVERED IN CONFIDENCE AND RELIANCE THAT SUCH INFORMATION WILL NOT BE COPIED OR MADE AVAILABLE TO ANY THIRD PARTY WITHOUT THE WRITTEN CONSENT OF LEO PHARMA A/S - LEO PHARMACEUTICAL PRODUCTS LTD. A/S

14 LIST OF APPENDICES

Appendix I: English translation of the Danish Data Protection Agency's terms and conditions for the processing of clinical trial data by medical companies

Appendix II: Declaration of Helsinki [last amended Seoul 2008]

Appendix III: Identification and Documentation of the Selected Treatment Areas

Appendix IV: Subject Safety and Study Gel Instructions

Appendix V: Local Skin Response Grading Scale

THIS DOCUMENT CONTAINS TRADE SECRETS, OR COMMERCIAL OR FINANCIAL INFORMATION, PRIVILEGED OR CONFIDENTIAL, DELIVERED IN CONFIDENCE AND RELIANCE THAT SUCH INFORMATION WILL NOT BE COPIED OR MADE AVAILABLE TO ANY THIRD PARTY WITHOUT THE WRITTEN CONSENT OF LEO PHARMA A/S - LEO PHARMACEUTICAL PRODUCTS LTD. A/S

Appendix I

English translation of the Danish Data Protection Agency's terms and conditions for the processing of clinical trial data by medical companies

LEO
THIS DOCUMENT CONTAINS TRADE SECRETS, OR COMMERCIAL OR FINANCIAL INFORMATION, PRIVILEGED OR CONFIDENTIAL, DELIVERED IN CONFIDENCE AND RELIANCE THAT SUCH INFORMATION WILL NOT BE COPIED OR MADE AVAILABLE TO ANY THIRD PARTY WITHOUT THE WRITTEN CONSENT OF LEO PHARMA A/S - LEO PHARMACEUTICAL PRODUCTS LTD. A/S

English translation of the Danish Data Protection Agency's terms and conditions for the processing of clinical trial data by medical companies*)

Listed below please find an English version of the general terms and conditions, set by the Danish Data Protection Agency, in cases involving authorisation for the processing of sensitive data by medical companies conducting continuous clinical trials of medical products. Circumstances may warrant some variation in the terms and conditions in concrete cases.

AUTHORISATION to process personal data

The Data Protection Agency hereby grants authorisation for processing of personal data for the purpose of the Company's continuous clinical trials, cf. section 50(1)(i) of the Danish Act on Processing of Personal Data. In this connection, the Data Protection Agency lays down the following terms:

General terms

Period of validity: The authorisation is valid until further notice.

1. LEO Pharma A/S - hereinafter called the "Company" is responsible for compliance with these present terms.

2. The data may be used for the sole purpose of performing clinical trials.

3. The Company shall once a year to the Data Protection Agency submit an overview of new, commenced trials as well as a corresponding overview of which trials have been completed in the past year. The overview shall contain as a minimum a title of the trial and name and address of the clinically responsible investigator.

*) Source: Letter dated 07-Mar-2003 from the Danish Data Protection Agency (Datatilsynet) to the Danish Association of Pharmaceutical Industries (LIF)

LEO

THIS DOCUMENT CONTAINS TRADE SECRETS, OR COMMERCIAL OR FINANCIAL INFORMATION, PRIVILEGED OR CONFIDENTIAL, DELIVERED IN CONFIDENCE AND RELIANCE THAT SUCH INFORMATION WILL NOT BE COPIED OR MADE AVAILABLE TO ANY THIRD PARTY WITHOUT THE WRITTEN CONSENT OF LEO PHARMA A/S - LEO PHARMACEUTICAL PRODUCTS LTD. A/S

4. Processing of personal data must be performed only by the controller or at the instance of the controller and at his responsibility. It is the responsibility of the controller that compliance of the terms is always observed when data are processed.

5. Any person processing personal data must be cognizant of these present terms.

6. The terms must be complied with also where processing is made by a data processor.

7. Facilities used for storage and processing of the data must be organized and fitted up in order to prevent unauthorized access.

8. Data processing must be organized in such a manner that data are protected against accidental or unlawful destruction, loss or impairment. Furthermore, the necessary control should be exercised to ensure that no inaccurate or misleading data are processed. Inaccurate or misleading data or data processed in contravention of the above Act or of these terms shall be rectified or erased.

9. Data must not be kept in a form that makes it possible to identify the data subject for a longer period than is necessary for the implementation of the project.

10. If results from the clinical trial are published this must be done so that it is impossible to identify individual persons.

11. It is a condition that compliance is made with related terms, if any, laid down in accordance with other legislation.

Electronic data

12. Identification data must be encrypted or replaced by a code number or the like. Alternatively, all data can be stored encrypted. Encryption keys, code keys etc. must be stored securely and separate from the personal data.

13. Access to project data can be obtained only through the use of a confidential password. A password must be replaced at least once a year and when conditions dictate it.

L E O

THIS DOCUMENT CONTAINS TRADE SECRETS, OR COMMERCIAL OR FINANCIAL INFORMATION, PRIVILEGED OR CONFIDENTIAL, DELIVERED IN CONFIDENCE AND RELIANCE THAT SUCH INFORMATION WILL NOT BE COPIED OR MADE AVAILABLE TO ANY THIRD PARTY WITHOUT THE WRITTEN CONSENT OF LEO PHARMA A/S - LEO PHARMACEUTICAL PRODUCTS LTD. A/S

14. If personal data are transferred over the Internet or other external network, the necessary security measures must be taken to ensure that the data do not come to the knowledge of any unauthorized third parties. As a minimum, the data must be encrypted during transmission. Transmission of sensitive personal data requires strong encryption. When using internal networks, it must be ensured that unauthorized persons are unable to obtain access to the data.

15. Removable storage media, safety copies of data etc. must be stored securely and under lock and so that unauthorized access is prevented.

Manual data

16. Manual clinical trial materials, including print-outs, failure lists and control lists etc., as well as other material which may directly or indirectly be linked with specific persons, must be stored securely under lock and so that unauthorized access is prevented.

Bio-bank and biological material

17. Samples with biological material and biological material in bio-banks must be stored securely under lock so that unauthorized access is prevented and in such a manner that it is ensured that the material is not lost, impaired or accidentally or illegally destroyed.

18. Biological material marked with civil registration number or name must be stored subject to special safety requirements.

19. The Company shall lay down internal guidelines for storage of biological material relative to the individual trials, and guidelines for storage of biological material in bio-banks. The guidelines shall be updated at least once a year.

Information to be provided to the data subject (trial subject)

20. Where the personal data are to be obtained from the trial subject (through interviews, questionnaires, clinical or para-clinical examination, treatment, observation etc.), detailed

L E O

THIS DOCUMENT CONTAINS TRADE SECRETS, OR COMMERCIAL OR FINANCIAL INFORMATION, PRIVILEGED OR CONFIDENTIAL, DELIVERED IN CONFIDENCE AND RELIANCE THAT SUCH INFORMATION WILL NOT BE COPIED OR MADE AVAILABLE TO ANY THIRD PARTY WITHOUT THE WRITTEN CONSENT OF LEO PHARMA A/S - LEO PHARMACEUTICAL PRODUCTS LTD. A/S

data about the project shall be distributed/forwarded to the trial subject. The trial subject must be informed of the name of the controller, the purpose of the project and of the fact that it is voluntary to participate and that consent may be withdrawn at any time. Where the data are to be disclosed to be used for other scientific or statistical purposes, the trial subject shall be advised also of the purpose of the disclosure and identity of recipients, if applicable.

The data subject should furthermore be advised that the project is notified to the Data Protection Agency in accordance with Act on Processing of Personal Data, and that the Agency has laid down specific terms to be complied with for the project for the purpose of protecting the data subject's privacy.

Disclosure of data

21. Disclosure of data identifying individuals to a third party may take place for other statistical or scientific purposes only.

22. Disclosure may be made only subject to prior approval of the Data Protection Agency. The Data Protection Agency may lay down new terms for the disclosure as well as the recipient's data processing.

    Disclosure of data may, however, take place in accordance with the below-mentioned authorisation to disclose data.

Right of access to personal data

23. The subject of the trial i.e. the data subject has no right of access to the data being processed concerning himself, cf. Section 32(4) of the Act on Processing of Personal Data. This Act does not prevent the grant of access.

Processing by a data processor

24. The Data Protection Agency's conditions shall apply also to processing by a data processor.

LEO
THIS DOCUMENT CONTAINS TRADE SECRETS, OR COMMERCIAL OR FINANCIAL INFORMATION, PRIVILEGED OR CONFIDENTIAL, DELIVERED IN CONFIDENCE AND RELIANCE THAT SUCH INFORMATION WILL NOT BE COPIED OR MADE AVAILABLE TO ANY THIRD PARTY WITHOUT THE WRITTEN CONSENT OF LEO PHARMA A/S - LEO PHARMACEUTICAL PRODUCTS LTD. A/S

25. When data are processed by a data processor, a written agreement shall be made between the controller and the data processor. The agreement shall stipulate that the data processor acts on behalf of the controller only and that the data must not be used for the data processor's own purposes. The controller shall furthermore request sufficient data from the data processor to ensure that the Data Protection Agency's terms can and will be complied with.

26. Where the data processor is established in another Member State it shall, furthermore, appear from the agreement that such other regulations on safety measures with regard to data processors that may be in force in the Member State in question, shall apply also to the data processor in question.

Erasure of data

27. Data in the individual trials shall be erased, made anonymous or destroyed no later than at the expiry of the storage period stipulated by the GCP-rules. It must not subsequently be possible to identify individuals participating in the trial.

28. Alternatively, the data may be transferred for further storage in archive in accordance with the rules of the archive legislation

29. Erasure of data from electronic media shall take place in such a manner that it is impossible to recover the data.

Transfer of data to third countries

30. Transfer of data to third countries, including for the purpose of processing by a data processor, requires the Data Protection Agency's prior approval.

    Transfer may take place in accordance with the below-mentioned transfer authorisation.

31. Transfer may, however, take place without approval of the Data Protection Agency if the data subject has given his explicit consent. The data subject can withdraw his consent.

THIS DOCUMENT CONTAINS TRADE SECRETS, OR COMMERCIAL OR FINANCIAL INFORMATION, PRIVILEGED OR CONFIDENTIAL, DELIVERED IN CONFIDENCE AND RELIANCE THAT SUCH INFORMATION WILL NOT BE COPIED OR MADE AVAILABLE TO ANY THIRD PARTY WITHOUT THE WRITTEN CONSENT OF LEO PHARMA A/S - LEO PHARMACEUTICAL PRODUCTS LTD. A/S

32. Transfer of data shall take place by courier or registered mail. In case of electronic transmission the necessary security measures shall be taken to prevent unauthorized access. As a minimum, the data must be safely encrypted during the entire transmission. Transfer of sensitive personal data requires strong encryption.

Changes of the notified data processing

33. The Data Protection Agency shall prior to implementation be notified of significant changes to the data processing (in the form of a change to an existing notification). Less significant changes may be notified to the Data Protection Agency subsequently, however not later than four (4) weeks after the implementation.

Discontinuance of notified data processing

34. The Company shall notify the Data Protection Agency immediately if the company discontinues carrying out the notified data processing.

AUTHORISATION to disclose data

In connection with its notification the Company has applied for authorisation to disclose data.

The Company has applied for authorisation to disclose data to relevant national and international health and medicines authorities in connection with an application for marketing authorization.

Furthermore, the Company has applied for authorisation to disclose data concerning adverse events to national and international health and medicines authorities according to national and international law on reporting of adverse events in clinical trials.

The Data Protection Agency hereby grants authorisation to the disclosure, cf. Section 10(3) of Act on Processing of Personal Data.

The authorisation in granted on the following terms:

L E O

THIS DOCUMENT CONTAINS TRADE SECRETS, OR COMMERCIAL OR FINANCIAL INFORMATION, PRIVILEGED OR CONFIDENTIAL, DELIVERED IN CONFIDENCE AND RELIANCE THAT SUCH INFORMATION WILL NOT BE COPIED OR MADE AVAILABLE TO ANY THIRD PARTY WITHOUT THE WRITTEN CONSENT OF LEO PHARMA A/S - LEO PHARMACEUTICAL PRODUCTS LTD. A/S

Period of validity: The authorisation is valid until further notice.

1. The relevant data may be disclosed to national and international health and medicines authorities in connection with an application for marketing authorization; to national and international health and medicines authorities according to national and international law on reporting of adverse events in clinical trials.

2. Only data required in the specific situation concerned may be disclosed.

3. The data may be disclosed to the recipient only in a form that does not identify individual persons. It must thus not be possible for the recipient on the basis of the received data alone to identify the persons related to the data.

4. The Company shall at any time be able to verify to the Data Protection Agency which transfers of data have been made.

AUTHORISATION to transfer personal data to third countries

In connection with its notification the Company has applied for authorisation to transfer personal data to third countries. The company wishes to transfer data for the purpose of data processing to be carried out by named data processors in third countries.

Furthermore, the Company wishes to transfer data to health and medicines authorities in third countries to comply with these countries' law on reporting of adverse events in clinical trials and in connection with applications for a marketing authorization.

According to Section 50(2) of Act on Processing of Personal Data, transfer of sensitive data to third countries can take place with the authorisation of the Data Protection Agency. According to section 50(5), the Data Protection Agency may lay down more detailed conditions for the carrying out of the processing operations for reasons of protection of the privacy of the data subject in question.

L E O
THIS DOCUMENT CONTAINS TRADE SECRETS, OR COMMERCIAL OR FINANCIAL INFORMATION, PRIVILEGED OR CONFIDENTIAL, DELIVERED IN CONFIDENCE AND RELIANCE THAT SUCH INFORMATION WILL NOT BE COPIED OR MADE AVAILABLE TO ANY THIRD PARTY WITHOUT THE WRITTEN CONSENT OF LEO PHARMA A/S - LEO PHARMACEUTICAL PRODUCTS LTD. A/S

The Data Protection Agency hereby grants authorisation to transfer data to third countries, cf. Section 50(2) of Act on Processing of Personal Data.

The authorisation is granted on the following terms:

Period of validity: The grant is valid until further notice.

1. Data may be transferred for processing by data processors with whom the Company has an agreement on data processing, and to health and medicines authorities in third countries in order to comply with the law of these countries on reporting of adverse events in clinical trials and in connection with an application for marketing authorization.

2. When data are transferred to and from third countries the necessary safety measurements must be taken to ensure that the data are not abused and to prevent unauthorized access. The data shall be delivered personally or sent by courier or registered post. Electronic transmission of data may take place only if the data are securely encrypted during the entire transmission. Transfer of sensitive personal data requires strong encryption.

3. Transfer of data to third countries takes place at the responsibility of the Company. The Company must therefore in each individual case assess whether the relevant transfer can take place, especially in consideration of the recipient's data safety. If it is assessed that the level of protection at the recipient's place is not adequate, transfer is not allowed.

4. The Company shall be able at any time to verify to the Data Protection Agency to which third countries data have been transferred and the purpose of this.

The following terms furthermore apply to transfer to data processors in third countries.

5. Processing of the data must take place only at the instance of the Company and at the Company's responsibility.

6. The Company shall always be in a position to notify the Data Protection Agency of the data processor's name and address.

LEO

THIS DOCUMENT CONTAINS TRADE SECRETS, OR COMMERCIAL OR FINANCIAL INFORMATION, PRIVILEGED OR CONFIDENTIAL, DELIVERED IN CONFIDENCE AND RELIANCE THAT SUCH INFORMATION WILL NOT BE COPIED OR MADE AVAILABLE TO ANY THIRD PARTY WITHOUT THE WRITTEN CONSENT OF LEO PHARMA A/S - LEO PHARMACEUTICAL PRODUCTS LTD. A/S

7. Prior to any transfer of data a written agreement shall be made with the recipient to the effect that the Data Protection Agency's conditions for processing of the data in Denmark shall be complied with when the data processor processes the data.

8. As responsible for the processing, the Company shall obtain information sufficient to ensure that the terms of the Data Protection Agency are complied with.

9. When the data are no longer to be processed by the data processor they must be erased or returned to the Company.

The terms of the Data Protection Agency are valid until further notice. The Data Protection Agency reserves the right to take up the terms for revisions at a later date, if required.

LEO
THIS DOCUMENT CONTAINS TRADE SECRETS, OR COMMERCIAL OR FINANCIAL INFORMATION, PRIVILEGED OR CONFIDENTIAL, DELIVERED IN CONFIDENCE AND RELIANCE THAT SUCH INFORMATION WILL NOT BE COPIED OR MADE AVAILABLE TO ANY THIRD PARTY WITHOUT THE WRITTEN CONSENT OF LEO PHARMA A/S - LEO PHARMACEUTICAL PRODUCTS LTD. A/S

Appendix II

Declaration of Helsinki [last amended Seoul 2008]

THIS DOCUMENT CONTAINS TRADE SECRETS, OR COMMERCIAL OR FINANCIAL INFORMATION, PRIVILEGED OR CONFIDENTIAL, DELIVERED IN CONFIDENCE AND RELIANCE THAT SUCH INFORMATION WILL NOT BE COPIED OR MADE AVAILABLE TO ANY THIRD PARTY WITHOUT THE WRITTEN CONSENT OF LEO PHARMA A/S - LEO PHARMACEUTICAL PRODUCTS LTD. A/S

Policy

WORLD MEDICAL ASSOCIATION DECLARATION OF HELSINKI
Ethical Principles for Medical Research Involving Human Subjects

Adopted by the 18th WMA General Assembly, Helsinki, Finland, June 1964, and amended by the:
29th WMA General Assembly, Tokyo, Japan, October 1975
35th WMA General Assembly, Venice, Italy, October 1983
41st WMA General Assembly, Hong Kong, September 1989
48th WMA General Assembly, Somerset West, Republic of South Africa, October 1996
52nd WMA General Assembly, Edinburgh, Scotland, October 2000
53th WMA General Assembly, Washington 2002 (Note of Clarification on paragraph 29 added)
55th WMA General Assembly, Tokyo 2004 (Note of Clarification on Paragraph 30 added)
59th WMA General Assembly, Seoul, October 2008

A. INTRODUCTION

1. The World Medical Association (WMA) has developed the Declaration of Helsinki as a statement of ethical principles for medical research involving human subjects, including research on identifiable human material and data.

   The Declaration is intended to be read as a whole and each of its constituent paragraphs should not be applied without consideration of all other relevant paragraphs.

2. Although the Declaration is addressed primarily to physicians, the WMA encourages other participants in medical research involving human subjects to adopt these principles.

3. It is the duty of the physician to promote and safeguard the health of patients, including those who are involved in medical research. The physician's knowledge and conscience are dedicated to the fulfilment of this duty.

4. The Declaration of Geneva of the WMA binds the physician with the words, "The health of my patient will be my first consideration," and the International Code of Medical Ethics declares that, "A physician shall act in the patient's best interest when providing medical care."

L E O
THIS DOCUMENT CONTAINS TRADE SECRETS, OR COMMERCIAL OR FINANCIAL INFORMATION, PRIVILEGED OR CONFIDENTIAL, DELIVERED IN CONFIDENCE AND RELIANCE THAT SUCH INFORMATION WILL NOT BE COPIED OR MADE AVAILABLE TO ANY THIRD PARTY WITHOUT THE WRITTEN CONSENT OF LEO PHARMA A/S - LEO PHARMACEUTICAL PRODUCTS LTD. A/S

5. Medical progress is based on research that ultimately must include studies involving human subjects. Populations that are underrepresented in medical research should be provided appropriate access to participation in research.

6. In medical research involving human subjects, the well-being of the individual research subject must take precedence over all other interests.

7. The primary purpose of medical research involving human subjects is to understand the causes, development and effects of diseases and improve preventive, diagnostic and therapeutic interventions (methods, procedures and treatments). Even the best current interventions must be evaluated continually through research for their safety, effectiveness, efficiency, accessibility and quality.

8. In medical practice and in medical research, most interventions involve risks and burdens.

9. Medical research is subject to ethical standards that promote respect for all human subjects and protect their health and rights. Some research populations are particularly vulnerable and need special protection. These include those who cannot give or refuse consent for themselves and those who may be vulnerable to coercion or undue influence.

10. Physicians should consider the ethical, legal and regulatory norms and standards for research involving human subjects in their own countries as well as applicable international norms and standards. No national or international ethical, legal or regulatory requirement should reduce or eliminate any of the protections for research subjects set forth in this Declaration.

B. BASIC PRINCIPLES FOR ALL MEDICAL RESEARCH

11. It is the duty of physicians who participate in medical research to protect the life, health, dignity, integrity, right to self-determination, privacy, and confidentiality of personal information of research subjects.

12. Medical research involving human subjects must conform to generally accepted scientific principles, be based on a thorough knowledge of the scientific literature, other relevant sources of information, and adequate laboratory and, as appropriate, animal experimenta-

L E O

THIS DOCUMENT CONTAINS TRADE SECRETS, OR COMMERCIAL OR FINANCIAL INFORMATION, PRIVILEGED OR CONFIDENTIAL, DELIVERED IN CONFIDENCE AND RELIANCE THAT SUCH INFORMATION WILL NOT BE COPIED OR MADE AVAILABLE TO ANY THIRD PARTY WITHOUT THE WRITTEN CONSENT OF LEO PHARMA A/S - LEO PHARMACEUTICAL PRODUCTS LTD. A/S

tion. The welfare of animals used for research must be respected.

13. Appropriate caution must be exercised in the conduct of medical research that may harm the environment.

14. The design and performance of each research study involving human subjects must be clearly described in a research protocol. The protocol should contain a statement of the ethical considerations involved and should indicate how the principles in this Declaration have been addressed. The protocol should include information regarding funding, sponsors, institutional affiliations, other potential conflicts of interest, incentives for subjects and provisions for treating and/or compensating subjects who are harmed as a consequence of participation in the research study. The protocol should describe arrangements for post-study access by study subjects to interventions identified as beneficial in the study or access to other appropriate care or benefits.

15. The research protocol must be submitted for consideration, comment, guidance and approval to a research ethics committee before the study begins. This committee must be independent of the researcher, the sponsor and any other undue influence. It must take into consideration the laws and regulations of the country or countries in which the research is to be performed as well as applicable international norms and standards but these must not be allowed to reduce or eliminate any of the protections for research subjects set forth in this Declaration. The committee must have the right to monitor ongoing studies. The researcher must provide monitoring information to the committee, especially information about any serious adverse events. No change to the protocol may be made without consideration and approval by the committee.

16. Medical research involving human subjects must be conducted only by individuals with the appropriate scientific training and qualifications. Research on patients or healthy volunteers requires the supervision of a competent and appropriately qualified physician or other health care professional. The responsibility for the protection of research subjects must always rest with the physician or other health care professional and never the research subjects, even though they have given consent.

17. Medical research involving a disadvantaged or vulnerable population or community is only justified if the research is responsive to the health needs and priorities of this population or community and if there is a reasonable likelihood that this population or commu-

LEO
THIS DOCUMENT CONTAINS TRADE SECRETS, OR COMMERCIAL OR FINANCIAL INFORMATION, PRIVILEGED OR CONFIDENTIAL, DELIVERED IN CONFIDENCE AND RELIANCE THAT SUCH INFORMATION WILL NOT BE COPIED OR MADE AVAILABLE TO ANY THIRD PARTY WITHOUT THE WRITTEN CONSENT OF LEO PHARMA A/S - LEO PHARMACEUTICAL PRODUCTS LTD. A/S

nity stands to benefit from the results of the research.

18. Every medical research study involving human subjects must be preceded by careful assessment of predictable risks and burdens to the individuals and communities involved in the research in comparison with foreseeable benefits to them and to other individuals or communities affected by the condition under investigation.

19. Every clinical trial must be registered in a publicly accessible database before recruitment of the first subject.

20. Physicians may not participate in a research study involving human subjects unless they are confident that the risks involved have been adequately assessed and can be satisfactorily managed. Physicians must immediately stop a study when the risks are found to outweigh the potential benefits or when there is conclusive proof of positive and beneficial results.

21. Medical research involving human subjects may only be conducted if the importance of the objective outweighs the inherent risks and burdens to the research subjects.

22. Participation by competent individuals as subjects in medical research must be voluntary. Although it may be appropriate to consult family members or community leaders, no competent individual may be enrolled in a research study unless he or she freely agrees.

23. Every precaution must be taken to protect the privacy of research subjects and the confidentiality of their personal information and to minimize the impact of the study on their physical, mental and social integrity.

24. In medical research involving competent human subjects, each potential subject must be adequately informed of the aims, methods, sources of funding, any possible conflicts of interest, institutional affiliations of the researcher, the anticipated benefits and potential risks of the study and the discomfort it may entail, and any other relevant aspects of the study. The potential subject must be informed of the right to refuse to participate in the study or to withdraw consent to participate at any time without reprisal. Special attention should be given to the specific information needs of individual potential subjects as well as to the methods used to deliver the information. After ensuring that the potential subject has understood the information, the physician or another appropriately qualified individu-

L E O

THIS DOCUMENT CONTAINS TRADE SECRETS, OR COMMERCIAL OR FINANCIAL INFORMATION, PRIVILEGED OR CONFIDENTIAL, DELIVERED IN CONFIDENCE AND RELIANCE THAT SUCH INFORMATION WILL NOT BE COPIED OR MADE AVAILABLE TO ANY THIRD PARTY WITHOUT THE WRITTEN CONSENT OF LEO PHARMA A/S - LEO PHARMACEUTICAL PRODUCTS LTD. A/S

al must then seek the potential subject's freely-given informed consent, preferably in writing. If the consent cannot be expressed in writing, the non-written consent must be formally documented and witnessed.

25. For medical research using identifiable human material or data, physicians must normally seek consent for the collection, analysis, storage and/or reuse. There may be situations where consent would be impossible or impractical to obtain for such research or would pose a threat to the validity of the research. In such situations the research may be done only after consideration and approval of a research ethics committee.

26. When seeking informed consent for participation in a research study the physician should be particularly cautious if the potential subject is in a dependent relationship with the physician or may consent under duress. In such situations the informed consent should be sought by an appropriately qualified individual who is completely independent of this relationship.

27. For a potential research subject who is incompetent, the physician must seek informed consent from the legally authorized representative. These individuals must not be included in a research study that has no likelihood of benefit for them unless it is intended to promote the health of the population represented by the potential subject, the research cannot instead be performed with competent persons, and the research entails only minimal risk and minimal burden.

28. When a potential research subject who is deemed incompetent is able to give assent to decisions about participation in research, the physician must seek that assent in addition to the consent of the legally authorized representative. The potential subject's dissent should be respected.

29. Research involving subjects who are physically or mentally incapable of giving consent, for example, unconscious patients, may be done only if the physical or mental condition that prevents giving informed consent is a necessary characteristic of the research population. In such circumstances the physician should seek informed consent from the legally authorized representative. If no such representative is available and if the research cannot be delayed, the study may proceed without informed consent provided that the specific reasons for involving subjects with a condition that renders them unable to give informed consent have been stated in the research protocol and the study has been approved by a

L E O
THIS DOCUMENT CONTAINS TRADE SECRETS, OR COMMERCIAL OR FINANCIAL INFORMATION, PRIVILEGED OR CONFIDENTIAL, DELIVERED IN CONFIDENCE AND RELIANCE THAT SUCH INFORMATION WILL NOT BE COPIED OR MADE AVAILABLE TO ANY THIRD PARTY WITHOUT THE WRITTEN CONSENT OF LEO PHARMA A/S - LEO PHARMACEUTICAL PRODUCTS LTD. A/S

research ethics committee. Consent to remain in the research should be obtained as soon as possible from the subject or a legally authorized representative.

30. Authors, editors and publishers all have ethical obligations with regard to the publication of the results of research. Authors have a duty to make publicly available the results of their research on human subjects and are accountable for the completeness and accuracy of their reports. They should adhere to accepted guidelines for ethical reporting. Negative and inconclusive as well as positive results should be published or otherwise made publicly available. Sources of funding, institutional affiliations and conflicts of interest should be declared in the publication. Reports of research not in accordance with the principles of this Declaration should not be accepted for publication.

C. ADDITIONAL PRINCIPLES FOR MEDICAL RESEARCH COMBINED WITH MEDICAL CARE

31. The physician may combine medical research with medical care only to the extent that the research is justified by its potential preventive, diagnostic or therapeutic value and if the physician has good reason to believe that participation in the research study will not adversely affect the health of the patients who serve as research subjects.

32. The benefits, risks, burdens and effectiveness of a new intervention must be tested against those of the best current proven intervention, except in the following circumstances:

33. The use of placebo, or no treatment, is acceptable in studies where no current proven intervention exists; or

34. Where for compelling and scientifically sound methodological reasons the use of placebo is necessary to determine the efficacy or safety of an intervention and the patients who receive placebo or no treatment will not be subject to any risk of serious or irreversible harm. Extreme care must be taken to avoid abuse of this option.

35. At the conclusion of the study, patients entered into the study are entitled to be informed about the outcome of the study and to share any benefits that result from it, for example, access to interventions identified as beneficial in the study or to other appropriate care or benefits.

L E O
THIS DOCUMENT CONTAINS TRADE SECRETS, OR COMMERCIAL OR FINANCIAL INFORMATION, PRIVILEGED OR CONFIDENTIAL, DELIVERED IN CONFIDENCE AND RELIANCE THAT SUCH INFORMATION WILL NOT BE COPIED OR MADE AVAILABLE TO ANY THIRD PARTY WITHOUT THE WRITTEN CONSENT OF LEO PHARMA A/S - LEO PHARMACEUTICAL PRODUCTS LTD. A/S

36. The physician must fully inform the patient which aspects of the care are related to the research. The refusal of a patient to participate in a study or the patient's decision to withdraw from the study must never interfere with the patient-physician relationship.

37. In the treatment of a patient, where proven interventions do not exist or have been ineffective, the physician, after seeking expert advice, with informed consent from the patient or a legally authorized representative, may use an unproven intervention if in the physician's judgement it offers hope of saving life, re-establishing health or alleviating suffering. Where possible, this intervention should be made the object of research, designed to evaluate its safety and efficacy. In all cases, new information should be recorded and, where appropriate, made publicly available.

22.10.2008

L E O

THIS DOCUMENT CONTAINS TRADE SECRETS, OR COMMERCIAL OR FINANCIAL INFORMATION, PRIVILEGED OR CONFIDENTIAL, DELIVERED IN CONFIDENCE AND RELIANCE THAT SUCH INFORMATION WILL NOT BE COPIED OR MADE AVAILABLE TO ANY THIRD PARTY WITHOUT THE WRITTEN CONSENT OF LEO PHARMA A/S - LEO PHARMACEUTICAL PRODUCTS LTD. A/S

Appendix III

Identification and Documentation of the Selected Treatment Areas

Screening Visit

All patients qualifying for this study are to have two selected treatment areas as defined below;

1. “AK Treatment Area”: A contiguous area of 25 cm^2 of skin on the upper extremity (incl. dorsum manus) that contains 2 to 5 AK lesions. Additionally there must be at least one AK lesion located within 1 to 5 cm outside of the selected AK Treatment Area. The AK Treatment Area and the AK lesion outside the area will be identified on the transparency sheet at the screening visit.

2. “Normal Skin Treatment Area”: A contiguous area of 25 cm^2 of normal skin that has no or only minimal sun-damage from the inner upper arm. The Normal Skin Treatment Area will be identified on a transparency sheet at the screening visit.

The areas can be one the same or different arms and will be referred to as the "selected treatment areas". They will be documented using a "three-point landmark technique" on two study transparencies and with colour photographs at this visit. Note: PEP005 Gel should not be applied in intertriginous areas.

Instructions for the three-point landmark technique study transparency are described below. Transparencies (pre-marked 1 cm^2 grid) and markers will be provided to the study sites.

Three-point landmark technique for the study transparencies

1. Complete footer information on the transparency using a fine-tipped indelible marker.
2. Place the transparency over the selected treatment area (one transparency for each selected treatment area).
3. Map and label at least three anatomical landmarks on the transparency which are in the vicinity of the selected treatment area. These landmarks should not change during the study (e.g. scars, moles, birthmarks, bony landmarks, etc).
4. Mark the outline of the selected treatment area (contiguous 25 cm^2) on the provided 1 cm^2 grid transparency. Ensure that the selected treatment area contains 25 unit areas, each unit area being 1 cm^2 in size.
5. Mark the 2-5 AK lesions within the selected treatment area and 1 AK lesion outside the selected treatment area on the AK Treatment Area transparency. Remember to

L E O
THIS DOCUMENT CONTAINS TRADE SECRETS, OR COMMERCIAL OR FINANCIAL INFORMATION, PRIVILEGED OR CONFIDENTIAL, DELIVERED IN CONFIDENCE AND RELIANCE THAT SUCH INFORMATION WILL NOT BE COPIED OR MADE AVAILABLE TO ANY THIRD PARTY WITHOUT THE WRITTEN CONSENT OF LEO PHARMA A/S - LEO PHARMACEUTICAL PRODUCTS LTD. A/S

align your AK Treatment Area so that an AK lesion is in a corner. This AK lesion will be biopsied on Day 2.

Day 1

After subject eligibility has been confirmed, and all baseline procedures have been conducted, perform the following steps:

1. Mark the location of the baseline biopsies on the transparencies. Note: the biopsies should be within 1-5 cm in distance from the selected treatment areas.
2. Using the transparencies, mark the location of the selected treatment areas on the skin using the fine-tipped indelible marker.
3. Retain the transparencies as part of the subject's source documents.

Day 2

Once the subject has had the Day 2 biopsy on the AK lesions within the selected AK Treatment Area, perform the following steps:

1. Mark the location of the AK biopsy on the AK Treatment Area transparency. The treatment area for Day 2 will now exclude the biopsy site (by approximately 1 cm). Mark the change in the treatment area on the AK Treatment Area transparency only (no need to mark the skin).
2. Retain the transparencies as part of the subject's source documents.

Day 3

Once the subject has had the Day 3 biopsies within the selected treatment areas, perform the following steps:

1. Mark the location of the biopsies on the transparencies.
2. Retain the transparencies as part of the subject's source documents.

Days 8 and 29

Use the transparencies and photographs to locate the selected treatment area at each follow-up evaluation. When re-aligning the transparencies, use the documented anatomical markers to duplicate transparency placement.

THIS DOCUMENT CONTAINS TRADE SECRETS, OR COMMERCIAL OR FINANCIAL INFORMATION, PRIVILEGED OR CONFIDENTIAL, DELIVERED IN CONFIDENCE AND RELIANCE THAT SUCH INFORMATION WILL NOT BE COPIED OR MADE AVAILABLE TO ANY THIRD PARTY WITHOUT THE WRITTEN CONSENT OF LEO PHARMA A/S - LEO PHARMACEUTICAL PRODUCTS LTD. A/S

Appendix IV

Subject Safety and Study Gel Instructions

L E O

THIS DOCUMENT CONTAINS TRADE SECRETS, OR COMMERCIAL OR FINANCIAL INFORMATION, PRIVILEGED OR CONFIDENTIAL, DELIVERED IN CONFIDENCE AND RELIANCE THAT SUCH INFORMATION WILL NOT BE COPIED OR MADE AVAILABLE TO ANY THIRD PARTY WITHOUT THE WRITTEN CONSENT OF LEO PHARMA A/S - LEO PHARMACEUTICAL PRODUCTS LTD. A/S

LP0041-02

Patient Safety and Study Medication Instructions

Caution – Skin Irritant and Severe Eye Irritant: Avoid contact with eyes. If contact with your eyes should occur (for example, you rub your eyes after touching the treatment area), rinse your eyes thoroughly with plenty of water and proceed immediately to the nearest Emergency Room or Urgent Care Centre for treatment. Be sure to report the incident to your study doctor immediately. Assessment by an ophthalmologist should be arranged as soon as possible.

PEP005 topical study gel may be irritating to your skin. Representations of responses commonly seen with PEP005 Gel treatment are tabled below. Your response may be different to what is shown here. If you are concerned at all about your skin while on the study please contact your study doctor.

Expected Skin Responses to PEP005 Treatment

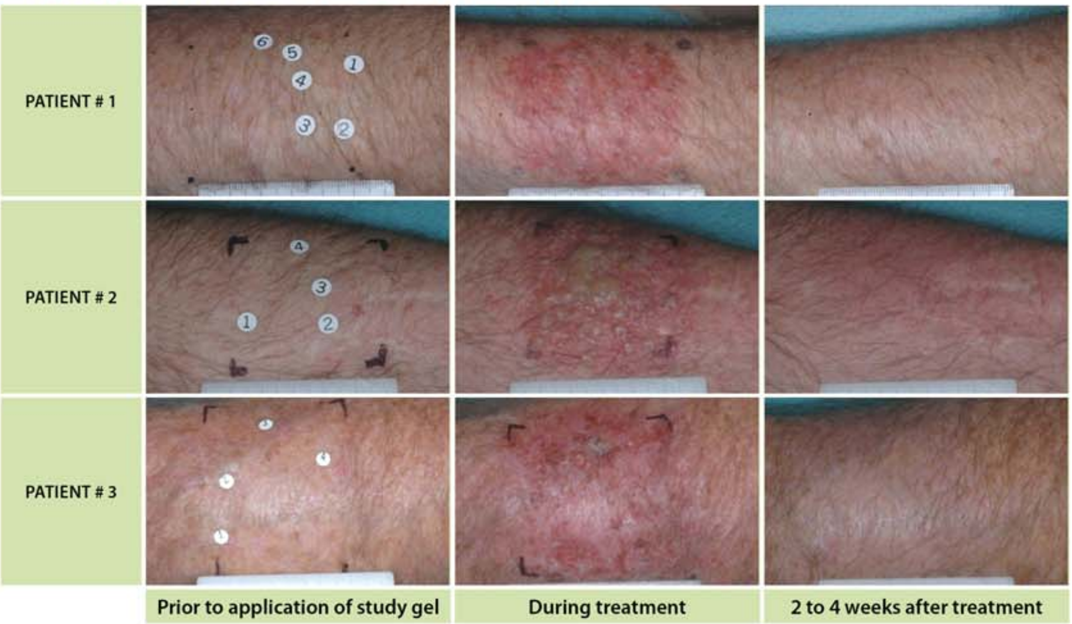

Patient Instructions

Study medication will be applied to the selected treatment area identified by your study doctor for two consecutive days.

Following **each** application of the study medication, the treatment area will be dried for 15 minutes prior to leaving the clinic and the instructions listed below should be followed.

Within 6 hours post treatment, **do not**;

- Touch the treatment area
- Wash the treatment area
- Engage in sports or other activities that may cause excessive sweating.

After the 6 hour wait period, you may wash gently using a mild, non-abrasive, non medicated soap/cleanser if required.

Within 24 hours post treatment, **do not**;

- Cover the treatment areas with tight clothing e.g. tight fitting t-shirt.

Within 15 days, **do not apply to the treatment area**;

- Moisturizers, sunscreen, or other over-the-counter topical products (including topical steroids).

Confirm the use of any topical products on the treatment areas with your study doctor first to make sure that they are allowed for this study.

For the duration of the study;

- Avoid excessive sun exposure (e.g., sunbathing, tanning booths)
- Wear protective loose clothing over the treated area to limit exposure.

If severe irritation occurs or persists, let your study doctor know immediately.

Do not allow non study participants and/or pets to come into contact with the treatment area for a period of 6 hours post application of the study medication. If any persons, patient or other, touch the treatment area wash hands/affected area with soap immediately. If it is touched by a pet, wash the pet immediately, as appropriate.

LP0041-02 Version 1.0

Contact your study doctor with any questions or concerns you may have about the study or reactions you may have to the study gel.

LEO
THIS DOCUMENT CONTAINS TRADE SECRETS, OR COMMERCIAL OR FINANCIAL INFORMATION, PRIVILEGED OR CONFIDENTIAL, DELIVERED IN CONFIDENCE AND RELIANCE THAT SUCH INFORMATION WILL NOT BE COPIED OR MADE AVAILABLE TO ANY THIRD PARTY WITHOUT THE WRITTEN CONSENT OF LEO PHARMA A/S - LEO PHARMACEUTICAL PRODUCTS LTD. A/S

Appendix V

Local Skin Response Grading Scale

L E O
THIS DOCUMENT CONTAINS TRADE SECRETS, OR COMMERCIAL OR FINANCIAL INFORMATION, PRIVILEGED OR CONFIDENTIAL, DELIVERED IN CONFIDENCE AND RELIANCE THAT SUCH INFORMATION WILL NOT BE COPIED OR MADE AVAILABLE TO ANY THIRD PARTY WITHOUT THE WRITTEN CONSENT OF LEO PHARMA A/S - LEO PHARMACEUTICAL PRODUCTS LTD. A/S

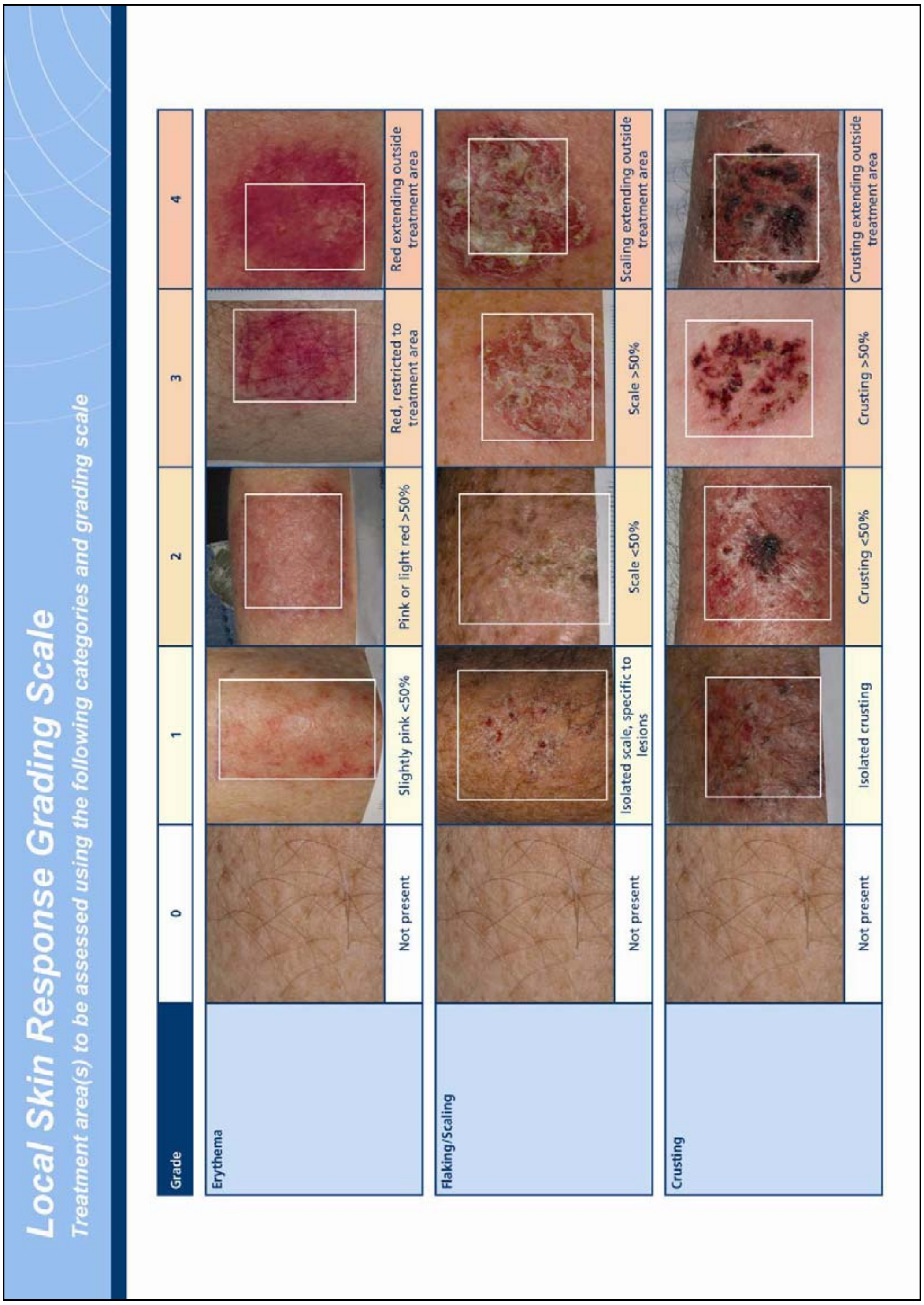

L E O
THIS DOCUMENT CONTAINS TRADE SECRETS, OR COMMERCIAL OR FINANCIAL INFORMATION, PRIVILEGED OR CONFIDENTIAL, DELIVERED IN CONFIDENCE AND RELIANCE THAT SUCH INFORMATION WILL NOT BE COPIED OR MADE AVAILABLE TO ANY THIRD PARTY WITHOUT THE WRITTEN CONSENT OF LEO PHARMA A/S - LEO PHARMACEUTICAL PRODUCTS LTD. A/S

Local Skin Response Continued…

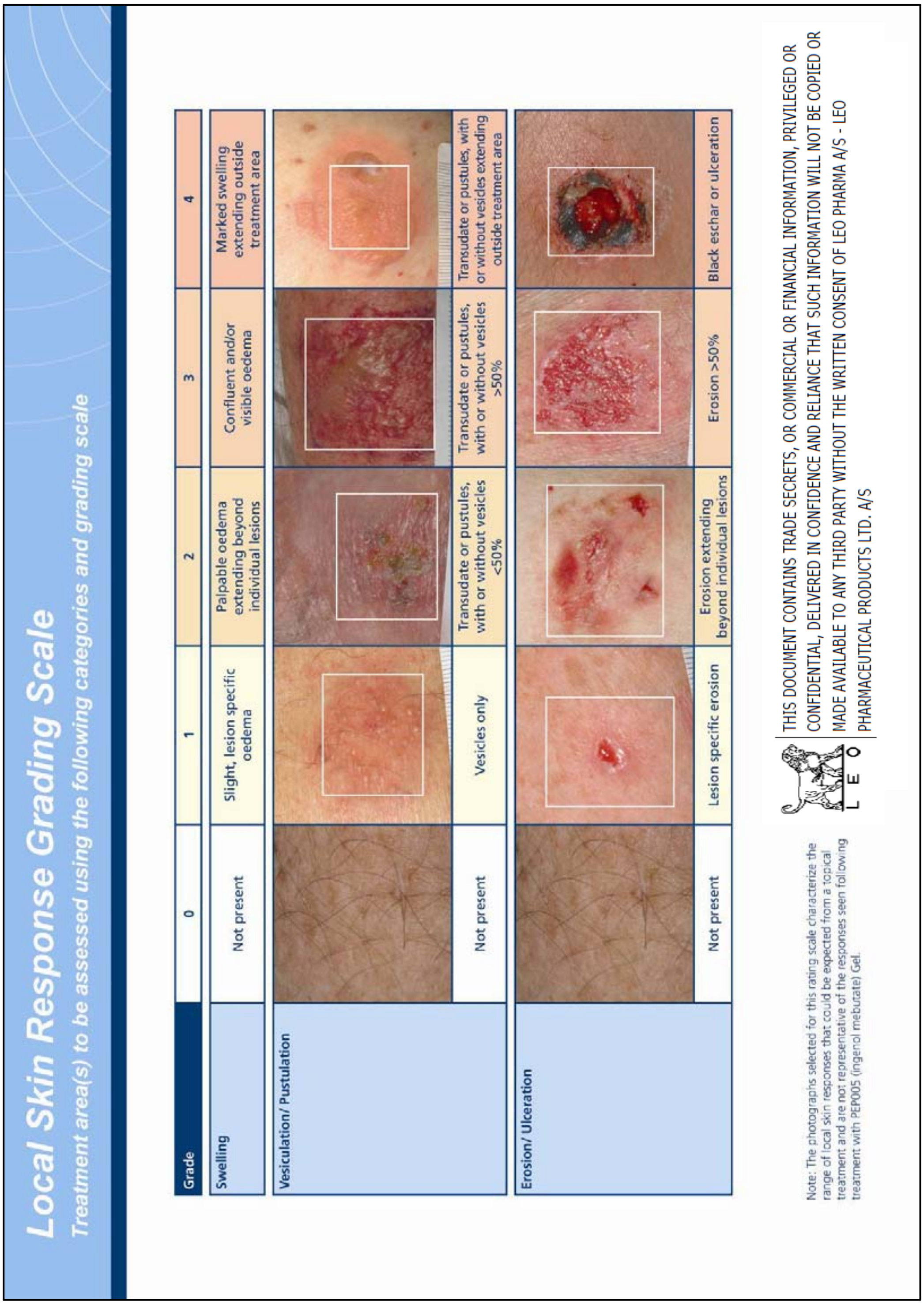

THIS DOCUMENT CONTAINS TRADE SECRETS, OR COMMERCIAL OR FINANCIAL INFORMATION, PRIVILEGED OR CONFIDENTIAL, DELIVERED IN CONFIDENCE AND RELIANCE THAT SUCH INFORMATION WILL NOT BE COPIED OR MADE AVAILABLE TO ANY THIRD PARTY WITHOUT THE WRITTEN CONSENT OF LEO PHARMA A/S - LEO PHARMACEUTICAL PRODUCTS LTD. A/S
