# Supplementary material for: Tumor-Preferential Induction of Immune Responses and Epidermal Cell Death in Actinic Keratoses by Ingenol Mebutate
Source: PLoS One. 2016 Sep 9;11(9):e0160096. doi: 10.1371/journal.pone.0160096 (PMC5017628; doi:10.1371/journal.pone.0160096)
Supplement: S1 Table — The analyses were carried out on logarithmically transformed data (and transformed back again, so the interpretations of the coefficients are multiplying factors of the original scale rather than differences on the log scale). Day 0, actinic keratosis (AK) versus uninvolved-skin (US): two-sided analysis of variance with the factors patient and type (AK/NS). AK or US, day 2 versus day 0: two-sided analysis of variance with factors patient and day. Day 2, AK versus NS: two-sided analysis of variance with factors patient type (AK/NS). (PDF) [file pone.0160096.s007.pdf]

CD1A_EPIDERMIS						
Day	Type	Analysis	Fold Change difference	Lower Confidence Level	Upper Confidence Level	p-value
0		AK vs NS	1.53	0.86	2.72	0.1382
.	AK	Day 2 vs Day 0	0.66	0.41	1.06	0.0844
.	NS	Day 2 vs Day 0	1.37	1.00	1.88	0.0470
2		AK vs NS	0.67	0.43	1.05	0.0805

CD1A DERMIS						
Day	Type	Analysis	Fold Change Difference	Lower Confidence Level	Upper Confidence Level	p-value
0		AK vs NS	3.94	1.87	8.30	0.0009
.	AK	Day 2 vs Day 0	0.89	0.49	1.63	0.7026
.	NS	Day 2 vs Day 0	1.20	0.72	2.01	0.4727
2		AK vs NS	2.63	1.59	4.36	0.0006

CD20_DERMIS						
Day	Type	Analysis	Fold Change Difference	Lower Confidence Level	Upper Confidence Level	p-value
0		AK vs NS	18.58	4.10	84.22	0.0005
.	AK	Day 2 vs Day 0	7.71	1.90	31.27	0.0060
.	NS	Day 2 vs Day 0	20.67	7.16	59.67	<.0001
2		AK vs NS	5.43	1.68	17.62	0.0067

CD4_EPIDERMIS						
Day	Type	Analysis	Fold Change difference	Lower Confidence Level	Upper Confidence Level	p-value
0		AK vs NS	5.12	2.17	12.04	0.0006
.	AK	Day 2 vs Day 0	6.91	2.46	19.42	0.0007
.	NS	Day 2 vs Day 0	28.23	14.40	55.35	<.0001
2		AK vs NS	1.42	0.60	3.40	0.4093

CD4_DERMIS						
Day	Type	Analysis	Fold Change Difference	Lower Confidence Level	Upper Confidence Level	p-value
0		AK vs NS	3.27	1.77	6.04	0.0006
.	AK	Day 2 vs Day 0	3.09	1.71	5.58	0.0006
.	NS	Day 2 vs Day 0	4.53	2.84	7.23	<.0001
2		AK vs NS	2.37	1.65	3.41	<.0001
2		AK vs NS	2.16	1.31	3.57	0.0042

CD8_DERMIS						
Day	Type	Analysis	Fold Change difference	Lower Confidence Level	Upper Confidence Level	p-value
0		AK vs NS	4.27	2.15	8.45	0.0002
.	AK	Day 2 vs Day 0	2.77	1.51	5.05	0.0019
.	NS	Day 2 vs Day 0	4.15	2.39	7.21	<.0001
2		AK vs NS	2.14	1.26	3.61	0.0065
**ICAM1_NUMBER**						
Day	Type	Analysis	Fold Change difference	Lower Confidence Level	Upper Confidence Level	p-value
0		AK vs NS	3.05	1.70	5.47	0.0006
.	AK	Day 2 vs Day 0	1.48	0.84	2.60	0.1646
.	NS	Day 2 vs Day 0	2.20	1.32	3.66	0.0037
2		AK vs NS	2.04	1.17	3.54	0.0139
**MPO EPIDERMIS**						
Day	Type	Analysis	Fold Change Difference	Lower Confidence Level	Upper Confidence Level	p-value
0		AK vs NS	18.12	4.07	80.60	0.0005
.	AK	Day 2 vs Day 0	120.7	22.17	657.2	<.0001
.	NS	Day 2 vs Day 0	4221	1047	17017	<.0001
2		AK vs NS	0.67	0.10	4.44	0.6608
**MPO DERMIS**						
Day	Type	Analysis	Fold Change Difference	Lower Confidence Level	Upper Confidence Level	p-value
0		AK vs NS	4.38	1.84	10.42	0.0017
.	AK	Day 2 vs Day 0	30.77	11.17	84.77	<.0001
.	NS	Day 2 vs Day 0	118.8	51.29	275.3	<.0001
2		AK vs NS	1.06	0.33	3.42	0.9138
