# Supplementary material for: Tumor-Preferential Induction of Immune Responses and Epidermal Cell Death in Actinic Keratoses by Ingenol Mebutate
Source: PLoS One. 2016 Sep 9;11(9):e0160096. doi: 10.1371/journal.pone.0160096 (PMC5017628; doi:10.1371/journal.pone.0160096)
Supplement: S2 Table — Untreated actinic keratosis (AK) lesions (AK0), AK-lesions treated with ingenol mebutate gel (IngMeb) for 1 (AK1) or 2 days (AK2), respectively, as well as uninvolved-skin without treatment (US0) or after 2 days of treatment with IngMeb (US2) were analyzed. The expression levels were normalized to minimally variable mRNAs GUSB and B2M. Dark green indicates a decrease in expression level of >2 ΔΔCt values comparing the median value (quantitative polymerase chain reaction [Q-PCR] data). Light green indicates a decrease in expression level of >1 ΔΔCt value comparing the median (qPCR data). Grey indicates differences in the median Ct values <1 ΔΔCt value. Dark red indicates an increase in expression level of >2 ΔΔCt values comparing the median (qPCR data). Orange indicates an increase in expression level of >1 ΔΔCt value comparing the median (qPCR data). Significance was tested with non-parametric Wilcoxon matched pair test. *P<0.05; **P < 0.01; ***P < 0.001. (PDF) [file pone.0160096.s008.pdf]

Genes of interest	Group of genes	AK0 compared to US0	US2 compared to US0	AK2 compared to AK0
CDSN	Epidermis-specific		↓*	↓*
DSC1	Epidermis-specific		↓***	↓***
FLG2	Epidermis-specific	↓**	↓***	↓***
KRT2	Epidermis-specific		↓***	↓***
KRT9	Epidermis-specific	↑***	↓*	↓***
TGM3	Epidermis-specific		↑NS	
MMP1	Fibroblast	↑NS	↑***	↑***
CCL18	Chemokine	↑NS	↑ ***	↑**
IL8	Chemokine	↑NS	↑***	↑***
CD4	Immune cell		↑NS	
CD207	Immune cell	↓*	↓NS	↓NS
CD34	Adhesion proteins	↓*	↓ ***	↓***
ICAM1	Adhesion proteins		↑***	↑**
SELL	Adhesion proteins	↑**	↑***	↑***
