# Supplementary material for: Tumor-Preferential Induction of Immune Responses and Epidermal Cell Death in Actinic Keratoses by Ingenol Mebutate
Source: PLoS One. 2016 Sep 9;11(9):e0160096. doi: 10.1371/journal.pone.0160096 (PMC5017628; doi:10.1371/journal.pone.0160096)
Supplement: S3 Table — The analyses were performed with untreated actinic keratosis (AK) lesions (AK0), AK-lesions treated with ingenol mebutate gel (IngMeb) for 1 (AK1) or 2 days (AK2), respectively, as well as with uninvolved-skin without treatment (US0) or after 2 days of treatment with IngMeb (US2). One minimally variable miRNA, namely miR-99b, was used for normalization. The median expression change (DCt) for each pair-wise comparison (AK0 versus US0, US2 versus US0, AK1 versus AK2, and AK2 versus AK0) is shown as well as its significance level (*P < 0.05; **P < 0.01; ***P < 0.001). Significance was tested with the non-parametric Wilcoxon matched pair test. The color coding indicates log2-fold up- (red >2 DCt, orange >1 DCt) or down- (dark green <-2 DCt) regulation. Grey shading indicates less than 2-fold (|DCt|<1) regulation. (PDF) [file pone.0160096.s009.pdf]

Genes of interest	Group of Genes and Gene information	AK0 compared to US0	US2 compared to US0	AK2 compared to AK0
miR-150	*Inflammation, innate immunity* *[Zidar et al., 2010]*	↑ NS (0.3)	↑*** (2.4)	↑** (2.2)
miR-31	*Inflammation*, negatively regulate FOXP3 *[Divekar et al., 2011]*	↑*** (7.2)	↑*** (5.3)	NS (0.3)
miR-31*	-	↑* (7.7)	↑ NS (1.2)	↑ NS (1.1)
miR-211	SCC oncogene *[Chu et al., 2013]*	↑** (1.4)	↓** (-0.7)	↓* (-0.9)
miR-21	*Chronic inflammation* *[Zibert et al., 2010]*	↑** (1.1)	↑* (1.6)	↑* (0.7)
miR-142-3p	*Chronic inflammation* *[Makino et al., 2012]*	↓ NS (-0.7)	↑** (2.4)	↑** (1.7)
miR-10a	Reduced levels critical for Treg function *[Kelada et al., 2013]*	↓ ** (-2.0)	↓ NS (-0.4)	↓ NS (-0.2)
miR-205	Epidermal differentiation *[Nissan et al., 2011]*	↓NS (-0.0)	↓*** (-0.6)	↓* (-0.5)
miR-203	Epidermal differentiation *[Yi et al., 2008]*	↓NS (-0.3)	↓ * (-0.4)	↓* (-0.5)
miR-193b	Negatively regulate wound healing *[Noh et al., 2011; Dayem et al., 2003)]*	↓** (-0.6)	↓ NS (-0.2)	↓ *** (-0.6)
miR-200a	-	↑ NS (0.3)	↓ NS (-2.2)	↑ NS (0.0)
